# Exercise training mitigates age-related cognitive decline by attenuating TMAO-induced inflammation

**DOI:** 10.1038/s41598-026-36354-z

**Published:** 2026-01-20

**Authors:** Rong Zhang, Lingfeng Li, Xiaoshuang Xi, Ziman Zhu, Tengteng Dai, Weijun Gong

**Affiliations:** 1 Beijing Rehabilitation Hospital, Capital Medical University, Beijing, 100144 China; 2 Department of Pain and Rehabilitation, Xinqiao Hospital, Army Medical University, Chongqing, 400035 China; 3 Beijing Rehabilitation Medical Academy, Capital Medical University, Beijing, 100144 China; 4 Department of Neurological Rehabilitation, Beijing Rehabilitation Hospital, Capital Medical University, Beijing, 100144 China

**Keywords:** Exercise training, TMAO, Inflammation, Age-related cognitive decline, Gut-brain axis, Pyroptosis, Cell biology, Diseases, Neuroscience

## Abstract

The metabolites produced by the gut microbiota play a role in age-related cognitive decline through the gut-brain axis. Within this axis, trimethylamine N-oxide (TMAO) permeates the intestinal epithelial barrier and enters systemic circulation, triggering inflammation in the central nervous system and ultimately leading to cognitive decline. However, it remains unclear whether exercise training’s specific mechanism for delaying age-related cognitive decline is associated with TMAO regulation and inhibition of neuroinflammation. The aging rat model was established by intraperitoneal injection of D-galactose in SD rats, while simultaneous exercise training and TMAO interventions were conducted. The effects of exercise on cognitive function were evaluated using the new object recognition (NOR) test, the Morris water maze (MWM) test, and the radial arm maze (RAM) test. Additionally, the expression levels of TMAO and NLRP3 inflammasome-related proteins in aging rats were measured using enzyme-linked immunosorbent assays (ELISA) and Western blotting (WB), respectively. A D-galactose-induced senescence model was established in HT22 cells. Following TMAO/DMB intervention, SPiDER-β-galactosidase (SPiDER-β-gal)-positive cells and NLRP3 inflammasome-related proteins were analyzed. To validate the regulatory role of TXNIP in TMAO-induced senescence-inflammation phenotypes, knockdown/overexpression experiments were conducted. Trx1-C32S mutant cells were utilized to verify that TMAO enhances the disulfide bond binding affinity between TXNIP and Trx1. Exercise training effectively delayed the cognitive dysfunction induced by D-galactose in aging rats, as evidenced by a 22.6% increase in the discrimination index in the NOR test, an 11.2% prolongation of time in the target quadrant and a 50% enhancement in the number of platform crossings in the MWM test, and a 41.8% improvement in working memory in the RAM test. This neuroprotective effect is potentially mediated through the inhibition of the intestinal metabolite TMAO (with plasma TMAO levels reduced by 40.3%) and subsequent modulation of the TXNIP-NLRP3-Caspase-1-GSDMD inflammatory pathway. The cellular experiments revealed that TMAO/DMB intervention modulates cellular senescence-inflammation phenotypes, with TXNIP acting as a positive regulator of the NLRP3 pathway. TMAO enhances TXNIP-mediated inhibition of the redox system by promoting disulfide bond formation at Trx1-C32, providing cellular-level evidence for the underlying mechanism.

**Supplementary Information:**

The online version contains supplementary material available at 10.1038/s41598-026-36354-z.

## Introduction

Cognitive decline serves as the hallmark manifestation of age-related degenerative changes in brain structure. It is primarily characterized by the deterioration of higher-order intelligent processing capabilities and a generalized slowing of information processing and perceptual speed, particularly in domains such as learning and memory^1,2^. These changes present considerable challenges for clinical prevention and treatment. Currently available pharmacological interventions—such as cholinesterase inhibitors and N-methyl-D-aspartate (NMDA) receptor antagonists—offer only symptomatic relief and do not halt disease progression. Although exercise training has been demonstrated to positively influence the delay of cognitive decline in older adults, its underlying regulatory mechanisms remain incompletely understood^3,4^. Thus, a deeper investigation into the molecular pathways through which exercise ameliorates age-related cognitive impairment is of considerable scientific and clinical importance.

Emerging evidence suggested that the gut microbiota plays a crucial role in modulating brain function and behavior, while its metabolites might contribute to age-related cognitive decline^5,6^. Could exercise potentially modulate age-related cognitive decline by regulating gut microbiota and its metabolites to suppress inflammation? However, the available evidence was limited.

Studies have demonstrated the beneficial effects of aerobic exercise on intestinal integrity and modulation of intestinal microbiota composition. For instance, a 1-week swimming training regimen consisting of 30 min/d reduced intestinal permeability in mice, while a planned wheel running program for 6 weeks increased the abundance of bifidobacteria^7^. Bifidobacterium has been shown to inhibit endotoxin production within the gut microbiota, thereby preserving intestinal integrity and regulating the activation of nuclear factor-kB (NF-kB) pathway to mitigate cognitive decline^8^. Apart from influencing intestinal permeability, exercise also exerts its regulatory effects on the immune system as a key pathway underlying its impact on gut microbiota. Research has revealed that exercise enhances crucial antioxidant enzymes (catalase and glutathione peroxidase), anti-inflammatory cytokines, and anti-apoptotic proteins in intestinal lymphocytes, while reducing pro-inflammatory cytokines and pro-apoptotic proteins^9^. Furthermore, metabolite profiling studies have indicated alterations in plasma levels following exercise training, such thatmoderate to high-intensity physical activity is associated with decreased trimethylamine N-oxide (TMAO) levels^10^. Male volunteer mice subjected to exercise exhibited lower TMAO concentrations in plasma compared to sedentary obese mice, which was accompanied by attenuated myocardial inflammation and fibrosis^11^.

TMAO is an intestinal microbial metabolite, and its precursor is trimethylamine (TMA). Some microbial populations in the intestine can produce TMA lyase, which metabolizes the complex containing TMA group into TMA, and then enters the liver through portal vein circulation and is oxidized by flavone monooxygenases (FMOs) into TMAO. TMAO plays an important role in aging-related cognitive dysfunction.Studies have shown that TMAO is a potential risk factor for neurodegenerative diseases.In the aging-related cognitive dysfunction disease model, the increase of plasma TMAO can be observed, which increases synaptic damage, reduces the expression level of synaptic plasticity-related proteins, and induces and aggravates the related cognitive dysfunction of model rats^12^.

NOD-like receptor protein 3 (NLRP3) is an important member of the inflammasome family, and NLRP3 has an important effect on the aging of the body. GSDMD is a member of the gasdermin family D (gasdermin D), a key executive protein of pyroptosis, and a direct substrate protein of cysteine aspartic acid protein hydrolase-1 (Caspase-1)-induced pyroptosis.In the process of aging, the damage products and metabolic waste produced by various organs activate NLRP3 and regulate the activation of Caspase-1, interleukin-18 (IL-18) and interleukin-1β (IL-1β) inflammatory factors, and trigger downstream inflammatory signaling pathways. IL-18 and IL-1β are key mediators between inflammation and aging, and play an important role in aging-related cognitive decline.Caspase-1 activated by NLRP3 cleaves GSDMD, forming pores on the cell membrane, resulting in the release of IL-18 and IL-1β from the cell, recruitment of inflammatory cells, and subsequent cell damage, which also triggers cell death through an inflammatory mechanism known as pyroptosis^13^.

Currently, substantial evidence indicates that cell pyroptosis is associated with cognitive impairment. Reports suggest that inhibiting the activation of the NLRP3 inflammasome can ameliorate microglia-mediated neuroinflammation and memory deficits in 3 × Tg AD (Alzheimer’s disease) mice^14^. Furthermore, the NLRP3 inflammasome is intricately linked to Aβ pathology; activation of this inflammasome in AD model microglia induces pyroptosis, exacerbating Aβ deposition in vivo^15^. Conversely, inhibition of the NLRP3 inflammasome significantly reduces Aβ accumulation, mitigates neuroinflammation in APP/PS1 mice, enhances hippocampal neuronal activity, and improves cognitive function^16^. Additional studies have demonstrated a close relationship between tau phosphorylation and regulation of the NLRP3 inflammasome. The absence of the NLRP3 inflammasome diminishes hyperphosphorylation of tau protein, thereby alleviating neurological symptoms associated with AD^17^. Moreover, inflammatory cytokines IL-18 and IL-1β released during pyroptosis can accelerate tau phosphorylation and aggregation by activating tau phosphokinase activity^18,19^.

TMAO has been shown to play an important role in activating NLRP3 inflammasome.In animal experiments, TMAO was injected into the carotid artery, and the levels of NLRP3 and IL-1β in the intima increased^20.^ Research by the team from Fudan University showed that TMAO can induce NLRP3 expression by enhancing nuclear localization of NF-kB, and activate NLRP3 through mitochondrial oxidative stress (ROS)^21^. In the process of oxidative stress, the upstream protein of NLRP3, thioredoxin-interacting protein (TXNIP), plays an important role. The inflammasome can be activated through the TXNIP pathway and regulate the maturation and secretion of inflammatory factors such as IL-18 and IL-1β, aggravating neuroinflammation and closely related to the decline in cognitive ability in the elderly^22^.

TXNIP functions as an endogenous inhibitor of Trx (thioredoxin) and serves a critical role in maintaining cellular redox balance^23,24^. Trx1 executes its antioxidant function primarily through a cysteine thiol-disulfide exchange mechanism^25^. Studies demonstrate that TXNIP binds to and inhibits Trx1, and both proteins participate in redox-dependent signaling pathways, including NLRP3 inflammasome activation^26,27^. Research indicates that in an AMD mouse model, Nrf2 negatively regulates the NLRP3 inflammasome through the Trx1/TXNIP complex^28^.

Inflammation is directly associated with cognitive decline in the elderly. For instance, several studies have indicated that inflammatory factors such as IL-18 and IL-1β in serum can serve as predictors of cognitive impairment in aged rats. Within primary neuron cultures derived from the human central nervous system, active Caspase-1 triggers the maturation of IL-18 and IL-1β, which are linked to gliosis and memory disorders. Cognitive decline can be delayed or reversed through Caspase-1 ablation or inhibitor treatment^29,30^. Furthermore, Wang Zhi et al. demonstrated a significant increase in NLRP3 expression within the hippocampus of aged mice compared to young mice^29^. Wang Ting et al. observed a notable enhancement in NLRP3 and Caspase-1 expression within the hippocampus of aged rats^31^. These findings highlight the crucial role played by the NLRP3-Caspase1-GSDMD pathway in age-related cognitive decline.

## Materials and methods

### Animal experiments

#### Animals

6-month-old male Sprague–Dawley rats were bred under specific pathogen-free (SPF) conditions and obtained from Beijing Sibeifu Biotechnology Co., Ltd. A total of fifty SD rats weighing 380–470 g were purchased, with ten rats allocated to each of the five groups. The animals were housed at the Animal Platform Center of the Basic Laboratory in Beijing Rehabilitation Hospital, affiliated with Capital Medical University (license number SCXK [Beijing] 2019–0010). Following a quarantine period, the rats were maintained under standard laboratory conditions with an indoor temperature range of 20–24 °C, relative humidity of 50–60%, and a 12-h light/dark cycle. After one week of acclimatization, random assignment using a computer-generated random number method resulted in five experimental groups consisting of ten rats per group: control group (Con), D-galactose-induced aging model group (D-gal), D-gal + exercise intervention group (D-gal + exe), D-gal + trimethylamine N-oxide treatment group (D-gal + TMAO), and D-gal + TMAO combined with exercise intervention group (D-gal + TMAO + exe). All animal procedures were performed in accordance with the protocols approved by the Animal Experiment Ethics Committee of Beijing Rehabilitation Hospital, which is affiliated with Capital Medical University (Ethics Approval No.: 2022bkky-148). This study was reported in compliance with the ARRIVE guidelines.

#### Drug administrations and physical exercise intervention

The Control group rats were intraperitoneally injected with saline at a dose of 2 mL/kg, while the other groups received intraperitoneal injections of D-galactose at a dose of 200 mg/(kg·d) to induce the aging animal model^31,32,33^. Additionally, the D-gal + TMAO group and the D-gal + TMAO + exe group were also administered intraperitoneal injections of TMAO at a dose of 200 mg/(kg·d). Moreover, the D-gal + exe group and the D-gal + TMAO + exe group underwent progressive-intensity treadmill training for 12 weeks.The exercise regimen consisted of two sessions per day, lasting 10 min each, with an intensity of 6 r/min, conducted six days a week for the initial four weeks. Subsequently, the speed was increased to 7 r/min for weeks 5–8 and further elevated to 8 r/min for weeks 9–12. This exercise protocol was adhered to until the completion of the twelfth week. After the Morris Water Maze (MWM) test, the rats were anesthetized with 2% sodium pentobarbital (Merck, Cat. No.Y0002194) at a dose of 30 mg/kg via intraperitoneal injection, and the anesthesia process was conducted out of the sight of other rats. Anesthesia was considered effective when the rats showed no response to gentle pinching of their paws with forceps. Subsequently, the rats were euthanized via an overdose of the same sodium pentobarbital (consistent with the anesthetic reagent) and their hippocampal tissues were extracted for subsequent experimental detection.

#### Behavioral tests

At the conclusion of all interventions, all rats underwent a series of behavioral assessments, which included the new object recognition test (NOR)^34^ the MWM,^35^ and the radial arm maze (RAM)^36^. These three experiments were designed to evaluate the spatial learning and memory capabilities of the rats, encompassing both working memory and reference memory. Through these behavioral assessments, we aimed to achieve a comprehensive understanding of the cognitive abilities of mice in spatial navigation tasks. For detailed experimental procedures, please refer to Supplementary Data 2.

#### UHPLC-MS/MS (ultra-high performance liquid chromatography-tandem mass spectrometry)

The plasma sample was thawed in an ice water bath and vortexed for 30 s. A volume of 10 μL of the test sample was transferred into a 1.5 mL Eppendorf tube. Subsequently, 40 μL of a 0.1% aqueous solution of formic acid was added, followed by the addition of 200 μL of a 0.1% acetonitrile extract containing formicacid. The sample was vortexed for another 30 s and then subjected to ultrasound treatment in an ice water bath for 15 min. Afterward, the sample was incubated at − 40 °C for 1on hour before being centrifuged at 4 °C and 12,000 rpm (13,800 ×*g*) with a radius of 8.6 cm for 15 min. The supernatant (100 μL) was collected into an LC injection vial for UHPLC-MS/MS analysis. The target compounds were separated using an Agilent 1290 Infinity II series ultra-performance liquid chromatograph equipped with a Waters ACQUITY UPLC BEH Amide column (100 × 2.1 mm, particle size 1.7 μm). The mobile phase consisted of a 0.1% aqueous solution of formic acid (A-phase) and 0.1% formic acid in acetonitrile (B-phase). The concentration of the target metabolite TMAO in sample CM (metabolite concentration, nmol/L) was determined by multiplying the amount of the target metabolite in sample CF with the final volume of the sample VF (volume, μL) and then dividing it by the volume of sample Vs (volume, μL).

#### Enzyme-linked immunosorbent assay (ELISA)

According to the manufacturer’s instructions, the specialized ELISA kit was employed for quantifying IL-1β and IL-18 levels in plasma. Thaw the plasma and subsequently centrifuge it before adding it to the designated well of the ELISA plate. Introduce both the standard and test samples, along with the biotinylated detection antibody, into separate wells of the ELISA plate. Following an incubation period, allow for interaction between the substance under examination within each sample and both capture and detection antibodies. After thorough washing to eliminate any unbound substances, introduce horseradish peroxidase (HRP)-labeled streptavidin into each well. Subsequent washing is followed by the addition of a chromogenic substrate (TMB), ensuring protection from light during chromogenic development. Terminate the reaction by introducing the termination solution and measure absorbance at the 450 nm wavelength accordingly. Calculate concentrations of IL-1β and IL-18 based on an established standard curve.

#### Reverse transcription quantitative polymerase chain reaction (RT-qPCR)

The hippocampus tissue was subjected to the Trizol extraction method for total RNA isolation, and the concentration of total RNA in the sample was determined. A mixture containing 1.0 μg of total RNA, reverse transcription primer T18 (50 μM), and nuclease-free H_2_O was incubated at 70 °C for 5 min, followed by cooling on ice. Subsequently, 4 μL of 5 × buffer, 1 μL of dNTP (10 mM), 0.5 μL RNase Inhibitor (MBI), and 1.0 μl M-MLV were added to the mixture along with nuclease-free H_2_O to a final volume of 20 μL. Then, an additional addition of 5×All-In-One RT MasterMix (4 μL) and nuclease-free H_2_O (6 μL) was made. Reverse transcription was performed using a thermal cycler set at temperatures of 42 °C for 60 min, followed by incubation at 70 °C for 15 min and cooling at 4 °C. When the temperature dropped below 50 °C, the samples were retrieved from the instrument. Real-time PCR Super Mix, double-distilled water, the designed primers, and extracted RNA samples were combined to form a reaction system with a volume of 10 μL. The amplification conditions included pre-denaturation at 95 °C for 20 s, followed by denaturation at 95 °C for 10 s, annealing at 60 °C for 30 s,and extension at 72 °C for 10 s, repeated for a total of 40 cycles. The relative expression level of TXNIP was calculated using the formula 2^−∆∆ct^.

**Table:** 

Target gene	Primer sequence	Product size
GAPDH	F: GAGCCAAAAGGGTCATCATCT	231bp
	R: AGGGGCCATCCACAGTCTTC	
TXNIP	F: TGGACGATGTGGACGACTCTCA	113bp
	R: GTTGTTGTTAAGGACGCACGGATC	
NLRP3	F: ATTACCCGCCCGAGAAAGG	40bp
	R: GACACACCTAGAAACGACGCT	

#### Western blot analysis

The hippocampal tissue was treated with lysis solution and incubated on ice for 20 min, followed by centrifugation at low temperature for 10 min to collect the supernatant. The sample was mixed with 5×Loading Buffer in a ratio of 4:1 and denatured at 95 °C for 10 min. Protein concentration was determined using the BCA kit, where A solution and B solution were mixed in a ratio of 50:1. The protein sample was diluted tenfold, incubated at 37 °C for 30 min, and the OD value was measured at a wavelength of 562 nm using an enzyme-linked immunosorbent assay instrument to calculate the protein concentration. SDS–polyacrylamide gel electrophoresis was performed by loading the protein samples from left to right in separate lanes. The concentrated gel ran at a voltage of 80 V while the separated gel ran at a voltage of 120 V. Transfer onto PVDF film pretreated with methanol occurred under constant flow (300 mA) and subsequent washing with TBST three times. The PVDF film was blocked with skimmed milk powder at room temperature for two hours. Primary antibody incubation took place overnight at 4 °C followed by washing three times with 1×TBST. The secondary antibody labeling occurred at room temperature for one hour, followed by another round of washing with TBST three times. Chemiluminescence detection was performed using ECL luminescence solution. Finally, the band intensity was quantitatively analyzed using Image J software.

### Cell validation

#### Construction and grouping of senescence model in HT22 cells

HT22 cells were purchased from Wuhan Procell Life Science & Technology Co., Ltd. These cells were seeded into 96-well culture plates, and a senescence model was established by treating the HT22 cells with 100 mM D-galactose for 72 h. Additionally, groups supplemented with TMAO (0.5 mM for 72 h) or DMB (0.5 mM for 72 h) were synchronized with the D-galactose treatment. The HT22 cells served as the blank control group (NS group). Lentiviral transfection was utilized to establish TXNIP overexpression (TXNIP-OE) and TXNIP knockdown (TXNIP-KD) groups in HT22 cells, which were treated with 0.5 mM for 72 h (TXNIP-OE-TMAO, TXNIP-KD-TMAO), alongside corresponding negative control groups (TXNIP-OE-NS, TXNIP-KD-NS). HT22 cell groups assigned to the mutation plasmid experiment underwent senescence model construction, Trx1 mutant plasmid transfection, and corresponding TMAO/DMB supplementation. The Trx1 mutant plasmid-transfected groups, without TMAO treatment, were divided into Trx1-MT-NS group (no TMAO) and Trx1-MT-TMAO group (with TMAO). The DMB-Trx1-MT group received initial DMB treatment followed by Trx1 mutant plasmid transfection, while the control group (DMB-BC) received only DMB treatment.

#### Construction of Trx1 mutant plasmids

The Trx1 vector and target gene sequence information were obtained from the gene bank. The following reagents were sequentially added: 1 μL vector DNA (1 μg/μL), 10 × buffer (4 μL), DdH₂O (32 μL), restriction enzyme B (1.5 μL) and restriction enzyme E (1.5 μL). The mixture underwent gentle pipetting for mixing, followed by reaction in a 37 °C water bath for 1–2 h. Following restriction digestion, agarose gel electrophoresis was conducted to recover the target fragment. Primers containing the C32S mutation were designed, and PCR amplification was performed using: 2 × PCR Buffer (25 μL), 1 μL dNTPs mix (10 mM each), 2 μL forward primer (10 μM), 2 μL reverse primer (10 μM), 1 μL template DNA (200 ng/μL), ddH₂O (18 μL), and phanta Super-Fidelity DNA Polymerase (1 μL). The PCR procedure encompassed pre-denaturation, denaturation, annealing, extension, final extension, and storage. The target fragment underwent ligation with the vector in an HB infusion™ One-Step Cloning Kit. Following transformation of competent DH5α cells, the bacterial solution was plated and cultured for 12–16 h. Single colonies underwent colony verification, and positive clones with correct verification proceeded to C32S sequencing. Correctly sequenced clones were used for plasmid extraction and subsequent cell transfection.

#### SPiDER-β-galactosidase (SPiDER-β-gal) staining

Preparation of stock solutions: SPiDER-βGal DMSO and Bafilomycin A1 DMSO stock solutions were prepared by adding 7 μl DMSO to SPiDER-βGal and 24 μl DMSO to Bafilomycin A1, followed by pipetting for dissolution. The stock solutions were stored at − 20 °C. Preparation of working solutions: The Bafilomycin A1 working solution was prepared through 1,000-fold dilution of the stock solution with medium. The SPiDER-βGal working solution was prepared by combining the SPiDER-βGal DMSO stock solution and Bafilomycin A1 DMSO stock solution, followed by 1,000-fold dilution with medium or HBSS. Staining procedure: Cells were seeded in 35-mm culture dishes and cultured overnight at 37 °C with 5% CO₂. After medium removal and washing with 2 ml medium, 1 ml Bafilomycin A1 working solution was added for 1 h incubation. Subsequently, 1 ml SPiDER-βGal working solution was added for 30 min incubation. Following supernatant removal and two washes with 2 mL medium, cells were examined under a fluorescence microscope or analyzed by flow cytometry.

#### Co-immunoprecipitation (Co-IP) technique

Cell lysis: Following treatment, cells from each group were harvested, the medium was aspirated, and cells were washed twice with PBS. After adding lysis buffer, cells were incubated on ice for 30 min. The cell lysate was transferred to a 1.5 mL centrifuge tube, centrifuged at 4 °C, 12,000 rpm for 15 min, and the supernatant was collected. Immunoprecipitation: The extracted non-denatured total protein (1 mg) was incubated with 3 μL of TXNIP IP antibody overnight at 4 °C on a shaker. The remaining sample was reserved for subsequent Western blot analysis. The overnight-incubated mixture was combined with Protein A/G magnetic beads and co-incubated overnight at 4 °C. Non-specifically bound proteins were eliminated through multiple cycles of centrifugation, supernatant aspiration, washing buffer resuspension, and incubation.Protein elution: The TXNIP-Trx1 precipitate complex bound to beads was mixed with 2×SDS-PAGE Loading Buffer and homogenized by pipetting. The sample underwent heat denaturation in a water bath to dissociate proteins from the beads, followed by immediate centrifugation at 14,000 × *g* for 1 min. The resulting supernatant, containing the immunoprecipitated sample, was stored at -80 ℃ for Western blot analysis.

### Data processing

For molecular biology experiments, three technical replicates were conducted per mouse, and the resulting data were averaged. The mean values derived from three mice per group (n = 3/group) were then used for intergroup statistical comparisons. In in vitro experiments, each assay was performed in triplicate wells to calculate an average count, and the entire experiment was independently repeated three times to ensure three biological replicates. For pathological experiments, one tissue section was collected from each of three mice per group and observed via immunofluorescence laser confocal microscopy. Three random fields of view were selected from each section for quantitative analysis of detection indicators, and the data were averaged. The mean values from the three mice per group (n = 3/group) were utilized for intergroup statistical comparisons.

### Statistical analysis

Statistical analyses were performed using Prism 8 software (for macOS). PASS software was employed to conduct power analysis, ensuring an appropriate sample size; a power value > 0.9 was deemed indicative of sufficient sample size in the experimental design. PASS software were used to conduct Power calculations to evaluate an appropriate sample size. The parameters were set as follows in the software: Alternative Hypothesis: Two-Sided; Alpha: 0.05; Sample size (Group allocation): Enter N1 and N2 individually; Effect size (Input Type): Means.

All data are presented as mean ± standard deviation (SD). The choice between parametric and nonparametric tests was determined by variance homogeneity. For intergroup comparisons: Student’s t-test was used for two-group comparisons, while one-way analysis of variance (ANOVA) was applied for multiple-group comparisons, followed by Sidak’s or Tukey’s multiple comparison test as appropriate. A p-value < 0.05 was considered statistically significant.

## Results

### Effecs of exercise training on plasma TMAO in aging rats

**Fig. 1:**
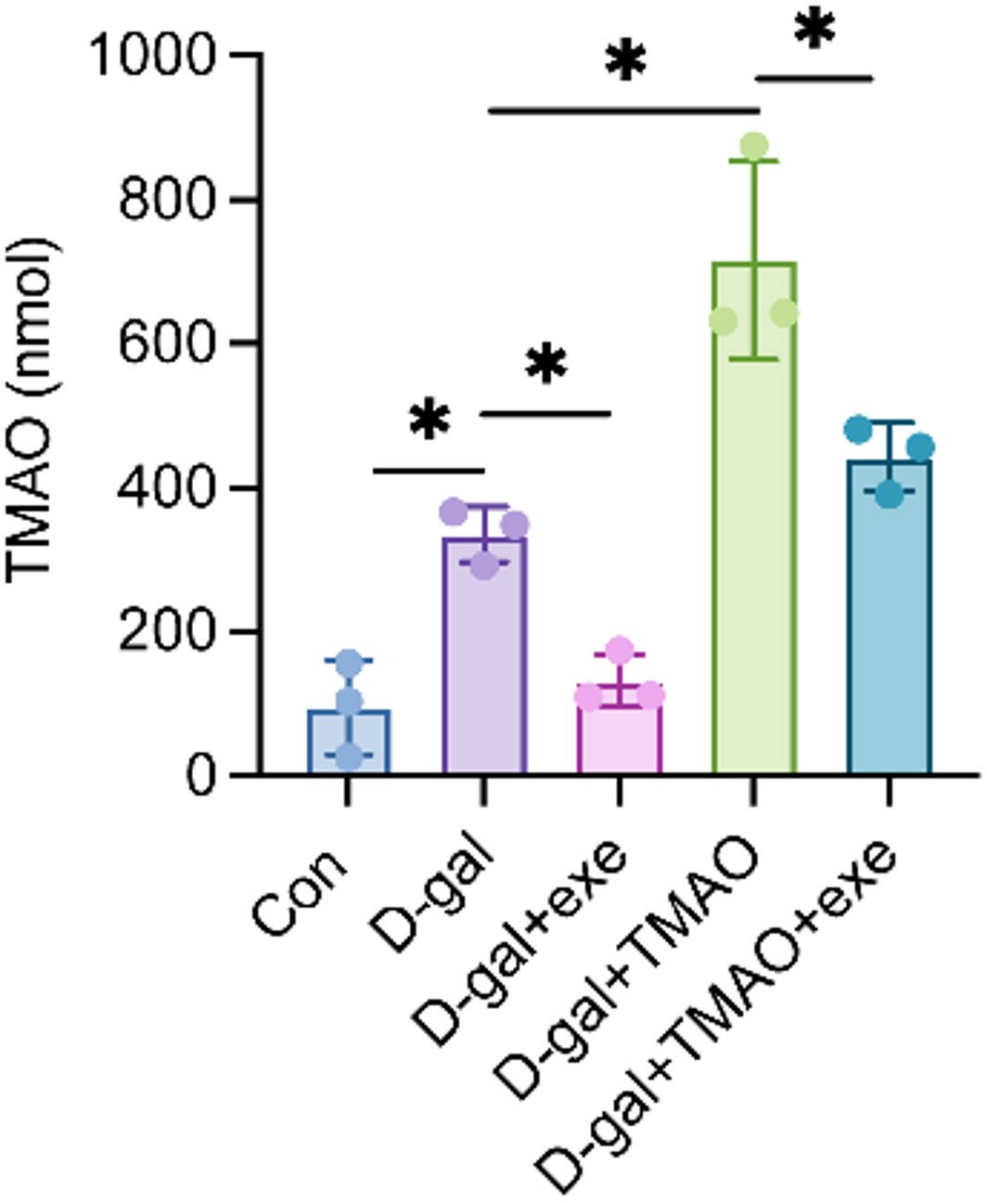
The plasma levels of TMAO were assessed in each experimental group of rats. The data were analyzed using one‐way analysis of variance and all data are expressed as the mean ± standard deviation. *P < *0.05* represents a statistically significant difference between the two groups.

The effect of exercise training on plasma TMAO levels in aging rats was evaluated using UHPLC-MS/MS analysis. Our results demonstrated that acute aging induced by D-galactose significantly increased plasma TMAO levels, as shown in Fig. 1, with the D-gal group showing markedly higher concentrations compared to the control group. In contrast, exercise training led to a reduction in plasma TMAO levels in aging rats, as indicated by the lower levels observed in the D-gal + exe group compared to the D-gal group. Additionally, TMAO intervention resulted in elevated plasma TMAO levels in aging rats, with the D-gal + TMAO group exhibiting higher concentrations than the D-gal group. Notably, exercise training mitigated the impact of TMAO intervention on plasma TMAO levels in aging rats, as evidenced by the lower levels found in the D-gal + TMAO + exe group compared to the D-gal + TMAO group.

### Effects of exercise training on spatial learning and memory ability of aging rats

**Fig. 2:**
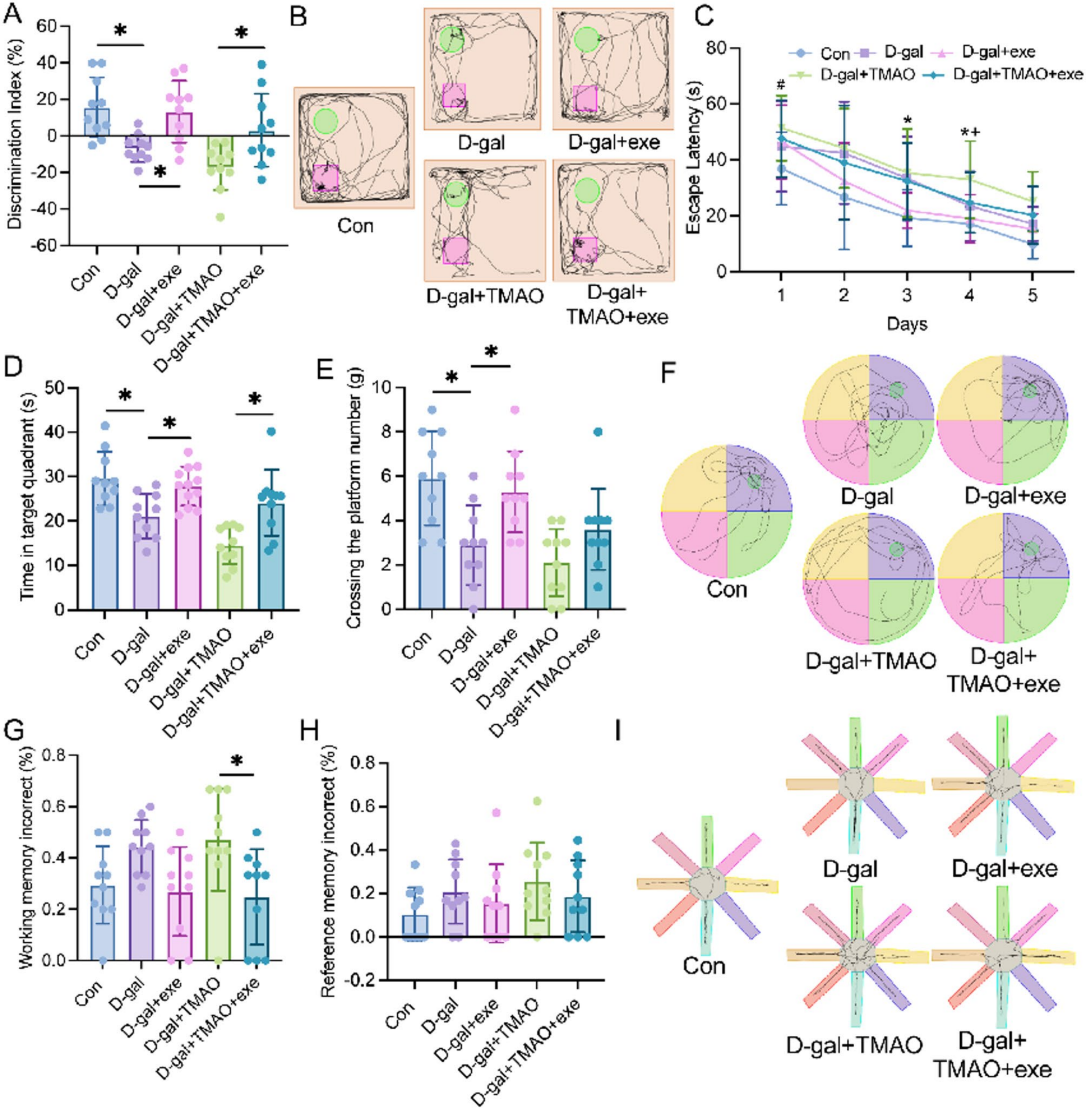
The cognitive performance of rats in each experimental group. (**A**) The discrimination index in the novel object recognition (NOR) test (n = 10). (**B**) The representative exploration trajectories of novel and familiar objects in the experiment of NOR test. (**C**) The escape latency in the morris water maze (MWM) test (n = 10). (**D**) The time spent in the target quadrant in the MWM test (n = 10). (**E**) The crossing the platform number in the MWM test (n = 10). (**F**) The representative swimming strategies of each group in the MWM test. (**G**) The working memory incorrect in the radial arm maze (RAM) test (n = 10). (**H**) The reference memory incorrect in the RAM test (n = 10). (**I**) The representative food acquisition strategies in the RAM test. The data were analyzed using one‐way (ABDEGH) or two-way (C) analysis of variance and all data are expressed as the mean ± standard deviation. *P < *0.05* represents a statistically significant difference between the two groups.

The effects of exercise training on spatial learning and memory abilities in aging rats were assessed using the Novel Object Recognition (NOR), Morris Water Maze (MWM), and Radial Arm Maze (RAM) tests (Fig. 2A–H). Results from the NOR test indicated that the D-gal group exhibited a significant increase in the discrimination index compared to the control group. Furthermore, the D-gal + exe group demonstrated a notable improvement in the discrimination index relative to the D-gal group, while the D-gal + TMAO group showed a decreasing trend in the discrimination index, although this was not statistically significant. In contrast, the D-gal + TMAO + exe group displayed a significantly higher discrimination index compared to the D-gal + TMAO group (Fig. 2A).

Figure 2B presents the typical trajectories of each experimental animal across the different groups during testing. The MWM test results revealed that the D-gal group had a longer escape latency on the first day compared to the control group. On the third day, the D-gal + exe group exhibited a shorter escape latency compared to the D-gal group, while the D-gal + tmao group showed an extended escape latency on the fourth day. Notably, the D-gal + tmao + exe group had a reduced escape latency on the fourth day compared to the D-gal + tmao group (Fig. 2C).

Additionally, the D-gal group spent significantly less time in the target quadrant and crossed the platform fewer times compared to the control group. The D-gal + exe group, however, demonstrated a significant increase in both time spent in the target quadrant and the number of platform crossings compared to the D-gal group. Although the D-gal + TMAO group exhibited a decreasing trend in both time spent in the target quadrant and the number of platform crossings, these differences were not statistically significant. In contrast, the D-gal + TMAO + exe group showed a significant increase in time spent in the target quadrant compared to the D-gal + TMAO group, although no significant difference was observed in the number of platform crossings (Fig. 2D, E). Figure 2F illustrates the representative swimming strategies employed by each group during the MWM experiment.

Results from the RAM experiment indicated that the D-gal + TMAO + exe group had a significant reduction in working memory errors compared to the D-gal + TMAO group (Fig. 2G). No significant differences were found in working memory errors or reference memory errors between the control group and the D-gal group, the D-gal group and the D-gal + exe group, or between the D-gal group and the D-gal + TMAO group (Figs. 2G, H). The representative food acquisition strategies utilized by each experimental group in the RAM are depicted in Fig. 2I.

### Effects of exercise training on the gene expression of TXNIP and NLRP3 in hippocampal tissue in aging rats

**Fig. 3:**
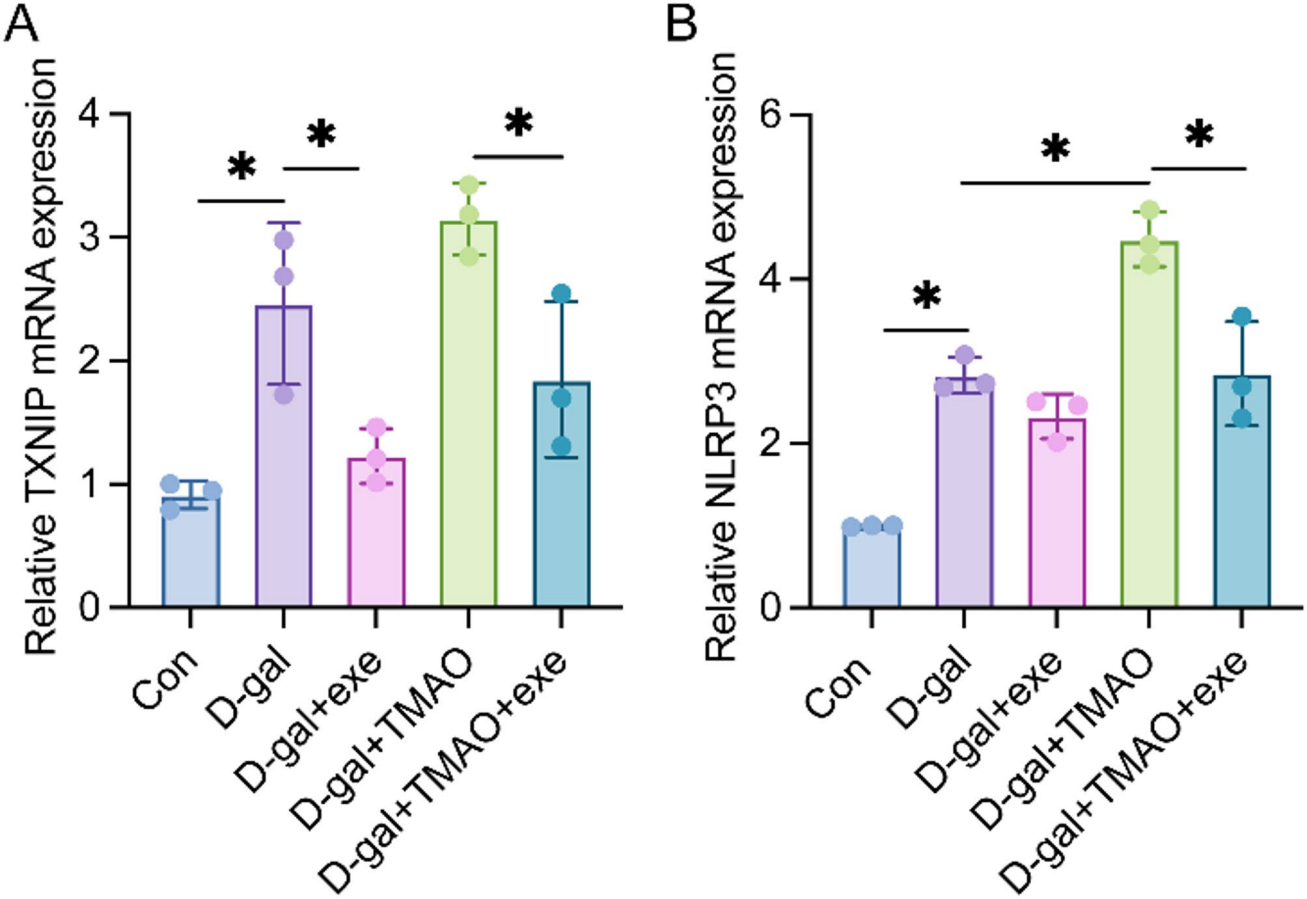
(**A**) TXNIP mRNA levels in hippocampus using qPCR (n = 3). (**B**) NLRP3 mRNA levels in hippocampus using qPCR (n = 3). The data were analyzed using one‐way analysis of variance and all data are expressed as the mean ± standard deviation. *P < *0.05* represents a statistically significant difference between the two groups.

As illustrated in Fig. 3, the levels of TXNIP and NLRP3 were significantly elevated in the hippocampal tissue of rats in the D-gal group compared to those in the control group. In contrast, the expression of TXNIP and NLRP3 was significantly reduced in the hippocampal tissue of rats in the D-gal + exe group compared to the D-gal group. Moreover, TXNIP and NLRP3 expression was markedly decreased in the hippocampal tissue of rats in the D-gal + TMAO + exe group compared to that observed in the D-gal + TMAO group.

### Effects of exercise training on protein expressions of TXNIP, NLRP3, pro-caspase-1, caspase-1 and GSDMD in hippocampal tissue in aging rats

**Fig. 4:**
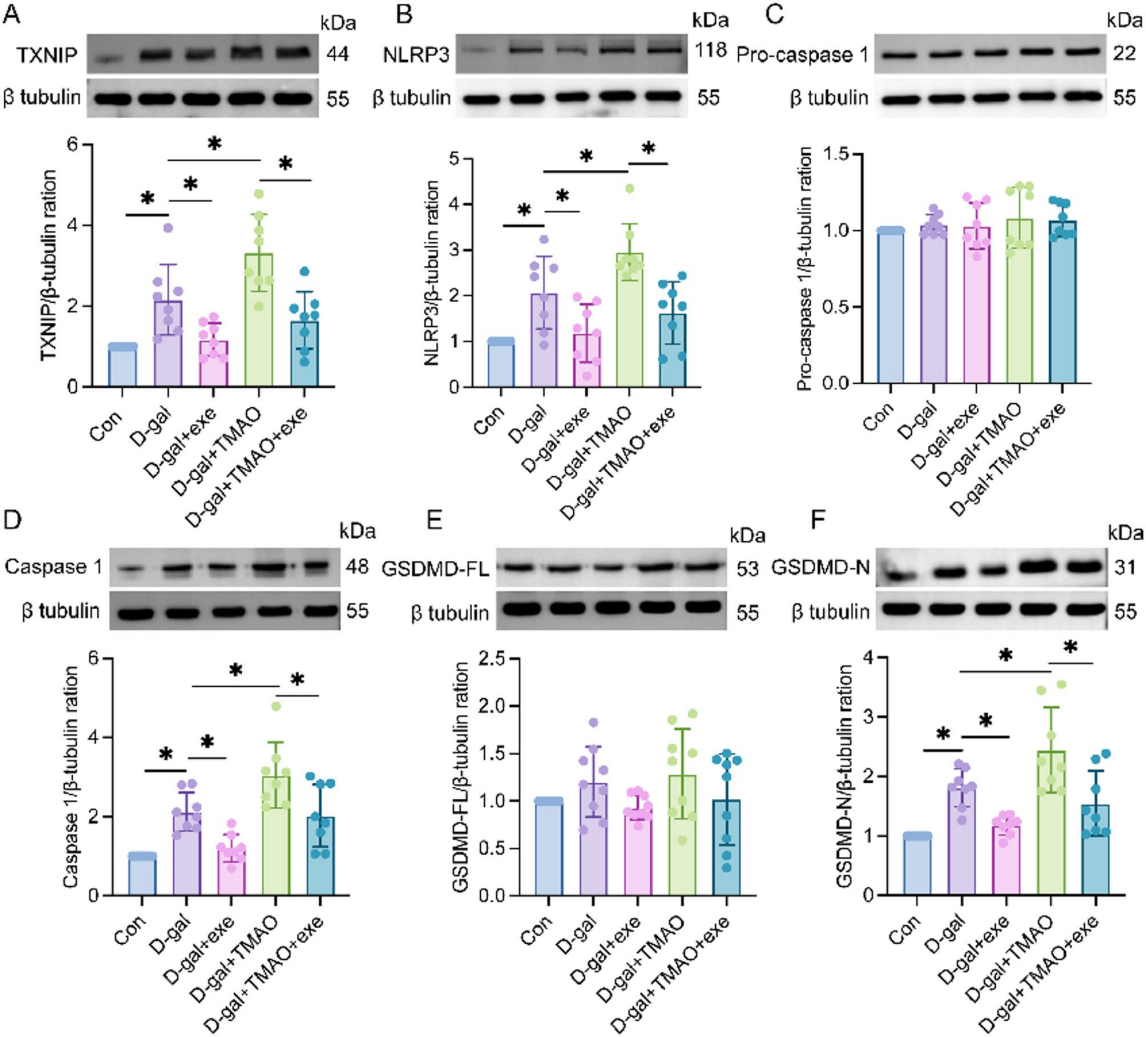
TXNIP, NLRP3, pro-caspase-1, caspase-1 and GSDMD levels in the hippocampus of rats. (**A**) Expression of TXNIP proteins in hippocampus (n = 8). The blots are representative of other replicates in each group. (**B**) Expression of NLRP3 proteins in hippocampus (n = 8). The blots are representative of other replicates in each group. (**C**) Expression of pro-caspase-1 proteins in hippocampus (n = 8). The blots are representative of other replicates in each group. (**D**) Expression of caspase-1 proteins in hippocampus (n = 8). The blots are representative of other replicates in each group. (**E**) Expression of GSDMD-FL proteins in hippocampus (n = 8). The blots are representative of other replicates in each group. (**F**) Expression of GSDMD-N proteins in hippocampus (n = 8). The data were analyzed using one‐way analysis of variance and all data are expressed as the mean ± standard deviation. *P < *0.05* represents a statistically significant difference between the two groups. The blots are representative of other replicates in those groups.

Western blot analysis revealed that the levels of TXNIP, NLRP3, caspase-1, and GSDMD-N in the hippocampus were significantly elevated in the D-gal group compared to the control group. Conversely, the levels of these proteins were significantly reduced in the D-gal + exe group compared to the D-gal group, as well as in the D-gal + TMAO + exe group compared to the D-gal + TMAO group. No significant differences were observed in the levels of pro-caspase-1 and GSDMD-FL among the control group and the other experimental groups (D-gal, D-gal + exe, D-gal + TMAO, and D-gal + TMAO + exe) (Fig. 4A–F).

### Effects of exercise training on the expression of IL-1β and IL-18 in hippocampal tissue in aging rats

**Fig. 5:**
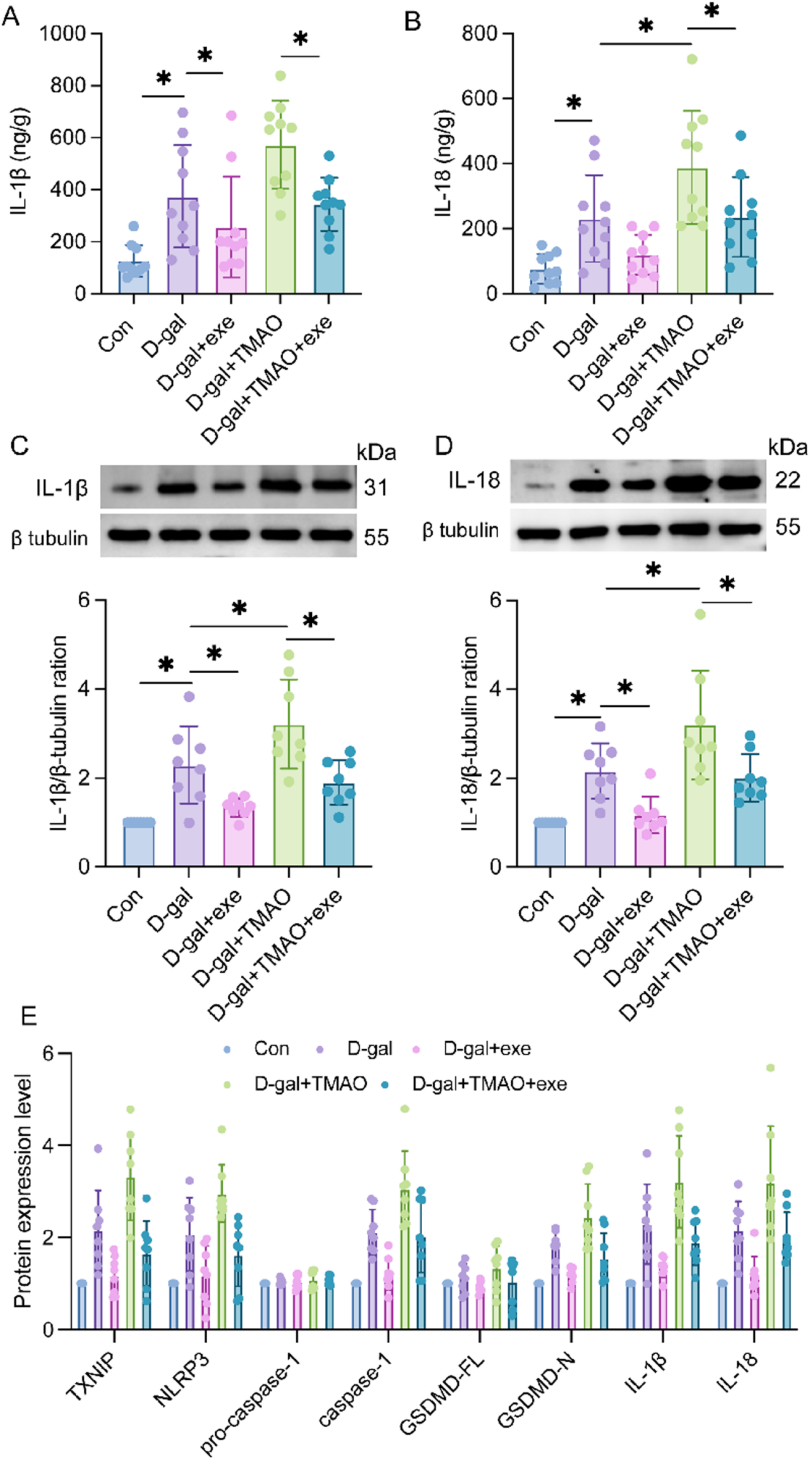
IL-1β and IL-18 levels in the plasma and hippocampus of rats. (**A**) IL-1β levels in the plasma (n = 10). (**B**) IL-18 levels in the plasma (n = 10). (**C**) Expression of IL-1β proteins in hippocampus (n = 10). The blots are representative of other replicates in each group. (**D**) Expression of IL-18 proteins in hippocampus (n = 10). (**E**) Outcomes of the two-way ANOVA, F_interaction_ (28, 284) = 3.588, P < 0.0001. The data were analyzed using one‐way analysis of variance and all data are expressed as the mean ± standard deviation. *P < *0.05* represents a statistically significant difference between the two groups. The blots are representative of other replicates in those groups.

The plasma ELISA results indicated that the levels of IL-1β and IL-18 were significantly elevated in the D-gal group compared to the control group. However, no significant differences in IL-1β and IL-18 levels were observed between the D-gal group and the D-gal + exe group. In contrast, the D-gal + TMAO group exhibited a significant increase in both IL-1β and IL-18 levels. Notably, exercise administration resulted in a significant reduction in IL-1β and IL-18 levels in the plasma when compared to the D-gal + TMAO group (Fig. 5A, B).

Western blot analysis demonstrated a significant increase in IL-1β and IL-18 levels in the hippocampal tissue of the D-gal group compared to the control group. Conversely, the D-gal + exe group showed a significant decrease in IL-1β and IL-18 levels in the hippocampal tissue compared to the D-gal group, while the D-gal + TAMO group exhibited an increase in IL-1β and IL-18 levels. Furthermore, when compared to the D-gal + TMAO group, the D-gal + TMAO + exe group displayed a significant decrease in IL-1β and IL-18 levels in the hippocampal tissue (Fig. 5C–E).

### TMAO exacerbates D-galactose-induced cellular senescence and inflammatory response via the TXNIP/NLRP3 pathway

**Fig. 6:**
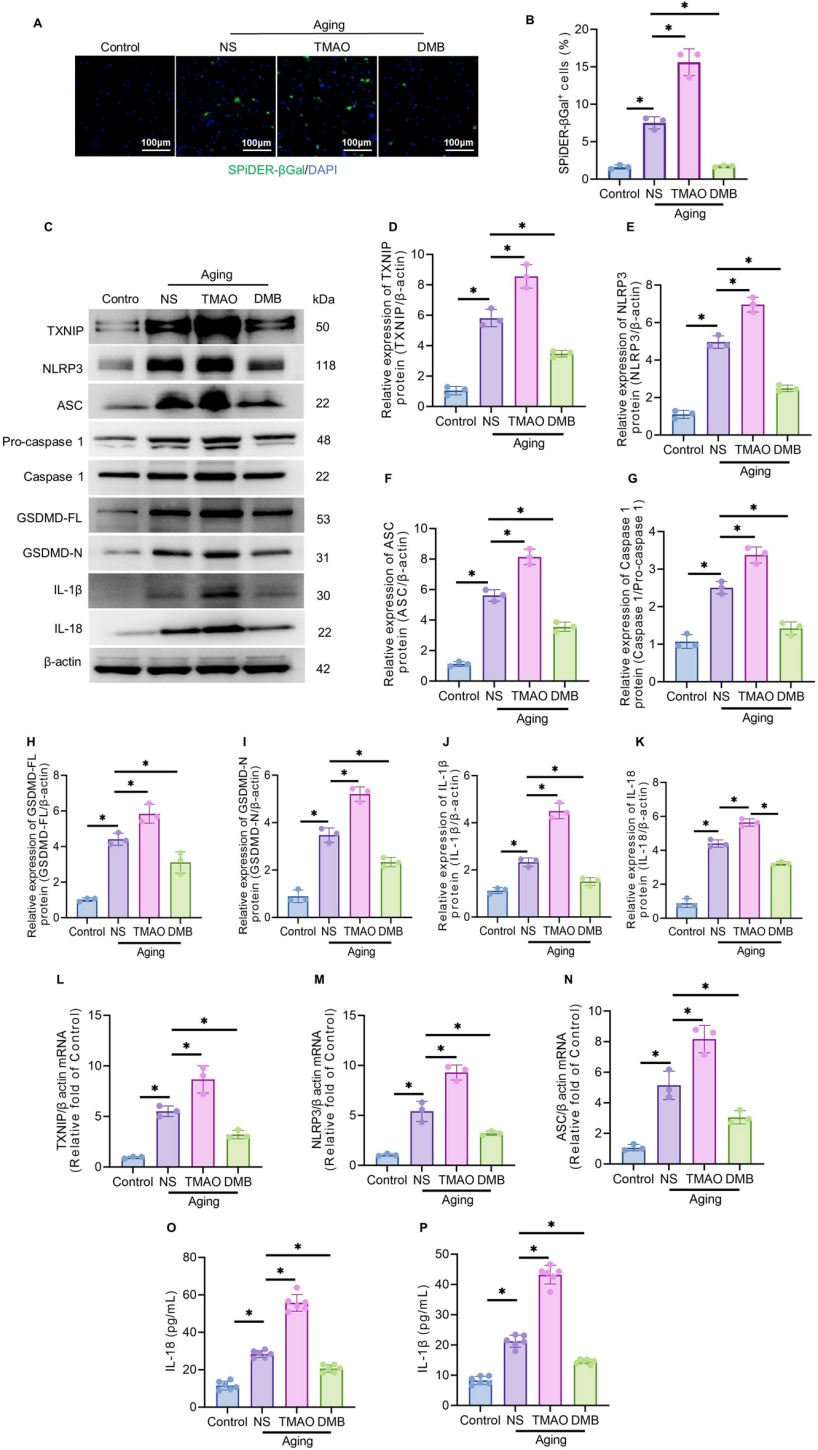
TMAO exacerbates D-galactose-induced cellular senescence via the TXNIP/NLRP3 pathway. (**A**) Representative fluorescence images of SPiDER-β-Gal + staining in senescent cells treated with TMAO/DMB. SPiDER-β-Gal + cells are stained green, and DAPI is stained blue. Scale bar = 100 μm. (**B**) Quantitative analysis of SPiDER-β-Gal + levels in each group shown in panel A. (**C**) Expression levels of TXNIP, NLRP3, ASC, Pro-caspase1, caspase-1, GSDMD-FL, GSDMD-N, IL-1β, and IL-18 in senescent cells treated with TMAO/DMB, as determined by Western blotting. (**D**–**K**) Quantitative analysis of protein levels of TXNIP, NLRP3, ASC, Pro-caspase1, caspase-1, GSDMD-FL, GSDMD-N, IL-1β, and IL-18 in each group shown in panel C. (**L**–**N**) mRNA levels of TXNIP, ASC, and NLRP3 in senescent cells treated with TMAO/DMB, as determined by RT-PCR. (**O**–**P**) The expression levels of IL-18 and IL-β were determined by ELISA. The data were analyzed using one‐way analysis of variance and all data are expressed as the mean ± standard deviation. *P < *0.05* represents a statistically significant difference between the two groups. The blots are representative of other replicates in those groups.

SA-β-gal, a widely recognized marker of cellular senescence, exhibits elevated expression in senescent cells. Cellular senescence was evaluated using SPiDER-β-Gal fluorescence staining (Fig. 6A). The percentage of SPiDER-β-Gal + cells showed a significant increase in the senescent (NS) group compared to the control group. TMAO treatment further elevated the percentage of SPiDER-β-Gal + cells in senescent cells relative to the NS group, while DMB, a TMAO inhibitor, significantly reduced this percentage (Fig. 6B).

To elucidate the mechanism through which TMAO exacerbates D-galactose-induced cellular senescence, the activity of the TXNIP/NLRP3 inflammatory pathway was evaluated using quantitative real-time polymerase chain reaction (qRT-PCR) and Western blotting. The NS group demonstrated increased mRNA levels of TXNIP, ASC, and NLRP3 compared to the control group, along with significantly elevated protein levels of TXNIP, NLRP3, ASC, Caspase1, GSDMD-FL, GSDMD-N, IL-18, and IL-1β. TMAO treatment further enhanced these mRNA and protein levels in senescent cells compared to the NS group. Conversely, DMB treatment significantly decreased these mRNA and protein levels compared to the TMAO group. These findings indicate that TMAO exacerbates D-galactose-induced cellular senescence through the TXNIP/NLRP3 pathway (Figs. 6C–N).

The expression of inflammatory factors in the D-galactose-induced cellular senescence model was assessed using enzyme-linked immunosorbent assay (ELISA). The levels of IL-18 and IL-1β were elevated in senescent cells (NS group) compared to the Control group. Senescent cells treated with TMAO exhibited significantly higher levels of IL-18 and IL-1β compared to the NS group. Treatment with the inhibitor DMB resulted in decreased levels of IL-18 and IL-1β compared to the TMAO group (Figs. 6O–P).

### Intervention of TXNIP positively regulates cellular senescence and inflammation exacerbated by TMAO

**Fig. 7:**
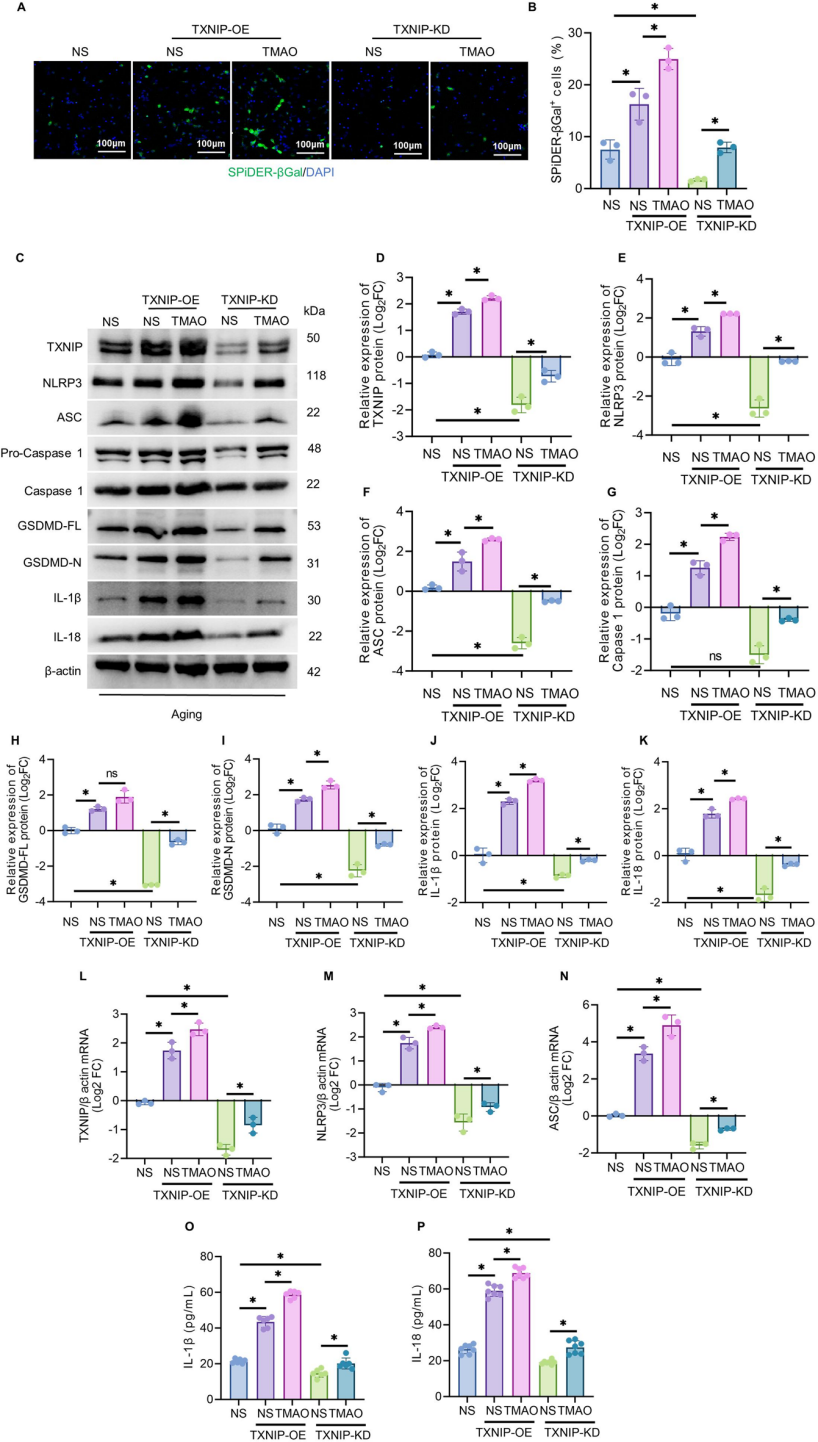
TXNIP intervention positively regulates cellular senescence and inflammation exacerbated by TMAO. (**A**) Representative fluorescence images of SPiDER-β-Gal + in HT22 cells from each group after TXNIP overexpression/knockdown and TMAO treatment. SPiDER-β-Gal + is stained green, DAPI is stained blue, scale bar = 100 μm. (**B**) Quantitative analysis of SPiDER-β-Gal + levels in each group shown in A. (**C**) Western blot of TXNIP, NLRP3, ASC, Pro-caspase1, caspase-1, GSDMD-FL, GSDMD-N, IL-1β and IL-18 proteins in HT22 cells from each group after TXNIP overexpression/knockdown and TMAO treatment. (**D**–**K**) Quantitative analysis of TXNIP, NLRP3, ASC, Pro-caspase1, caspase-1, GSDMD-FL, GSDMD-N, IL-1β and IL-18 protein levels in each group shown in C. (**L**–**N**) mRNA levels of TXNIP, ASC and NLRP3 in HT22 cells from each group determined by RT-PCR. (**O**–**P**) Expression levels of IL-18 and IL-β in HT22 cells from each group determined by ELISA. The data were analyzed using one‐way analysis of variance and all data are expressed as the mean ± standard deviation. *P < 0.05 represents a statistically significant difference between the two groups. The blots are representative of other replicates in those groups.

To investigate whether TXNIP intervention exacerbates cellular senescence and its regulatory mechanism, SPiDER-β-Gal fluorescence staining was conducted. As illustrated in Figs. 7A, B, compared to normal cells (NS group), the percentage of SPiDER-β-Gal + cells increased significantly following TXNIP overexpression, while decreasing significantly after TXNIP knockdown. In TXNIP-overexpressed cells treated with TMAO (TXNIP-OE-TMAO), the percentage of SPiDER-β-Gal + cells was notably higher than in the control group (TXNIP-OE-NS). Conversely, in TXNIP-knockdown cells treated with TMAO (TXNIP-KD-TMAO), the percentage of SPiDER-β-Gal + cells was markedly lower than in the control group (TXNIP-KD-NS).

To confirm the regulatory role of TXNIP in TMAO-exacerbated cellular senescence, the expression levels of mRNA and protein in the TXNIP/NLRP3-ASC-caspase-1 pathway were assessed through PCR and WB. As demonstrated in Fig. 7C–N, compared to normal cells (NS group), TXNIP overexpression elevated the mRNA levels of TXNIP, ASC, and NLRP3, and significantly increased the protein levels of TXNIP, NLRP3, ASC, Caspase1, GSDMD-FL, GSDMD-N, IL-18, and IL-1β. Conversely, TXNIP knockdown significantly reduced these mRNA and protein levels. In TXNIP-OE-TMAO cells, the aforementioned mRNA and protein levels were significantly higher than in TXNIP-OE-NS cells. Notably, in TXNIP-KD-TMAO cells, these levels were also significantly elevated compared to TXNIP-KD-NS cells.

The study further demonstrated that TXNIP intervention not only exacerbated cellular senescence but also enhanced the inflammatory response. ELISA results (Fig. 7O, P) indicated that compared to the NS group, IL-18 and IL-1β levels increased following TXNIP overexpression and decreased significantly after TXNIP knockdown. TMAO treatment significantly elevated IL-18 and IL-1β levels in both TXNIP-overexpressed and knockdown cells.

### In the TMAO-induced senescence model, the binding affinity between TXNIP and Trx1 at C32S is dependent on the amino acid residue at position 32 of Trx1.

**Fig. 8:**
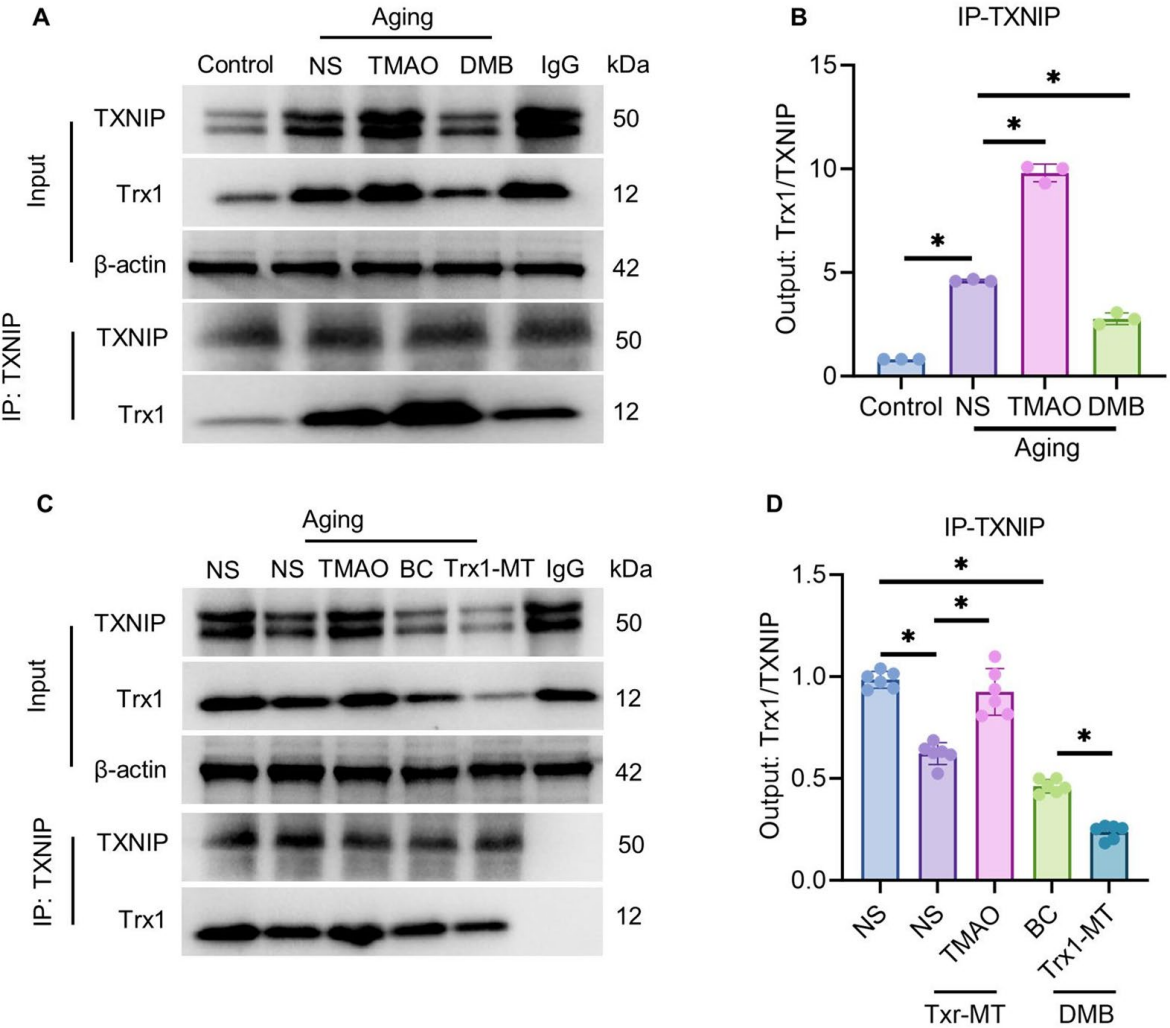
TMAO enhances the binding affinity between TXNIP and Trx1. (**A**) The most representative immunoblotting images of TXNIP and Trx1 protein binding in senescent cells treated with TMAO/DMB detected by Co-IP assay. Input serves as the loading control for total cellular lysate proteins, and IgG serves as the negative control for non-specific binding. (**B**) Quantitative analysis of the binding ability of TXNIP and Trx1 proteins shown in A. (**C**) The most representative immunoblotting images of TXNIP and Trx1 protein binding in senescent cells with Trx1 mutants and TMAO/DMB treatment detected by Co-IP assay. (**D**) Quantitative analysis of the binding ability of TXNIP and Trx1 proteins shown in C. The data were analyzed using one‐way analysis of variance and all data are expressed as the mean ± standard deviation. *P < 0.05 represents a statistically significant difference between the two groups. The blots are representative of other replicates in those groups.

Previous studies have demonstrated that TXNIP binds to Trx1[37]. To examine the effect of TMAO on the binding ability between TXNIP and Trx1 in the senescence model, the expression levels of TXNIP-Trx1 binding were assessed using co-immunoprecipitation (Co-IP) assay. As shown in Fig. 8A, B, compared to the Control group, the binding affinity between TXNIP and Trx1 increased significantly in the NS group (senescent HT22 cells). Compared to the NS group, TMAO treatment further enhanced the TXNIP-Trx1 binding affinity in the senescence model, while treatment with the TMAO inhibitor DMB significantly decreased this binding.

To investigate the relationship between TXNIP-Trx1 binding capacity and Trx1 in the TMAO-induced senescence model, we performed a mutation at the 32nd amino acid site of Trx1 and analyzed alterations in TXNIP-Trx1 binding. As illustrated in Fig. 8C, D, the binding affinity between TXNIP and Trx1 showed significant reduction in the Trx1-MT-NS group (Trx1 mutant + NS) compared to the senescence model control group (NS group). Despite the reduced TXNIP-Trx1 binding due to Trx1 mutation, TMAO intervention continued to promote their interaction. Treatment with the inhibitor DMB alone (DMB-BC group) diminished TXNIP-Trx1 binding relative to the senescence model control group (NS group). Additional treatment with the Trx1 mutant following DMB inhibition further reduced TXNIP-Trx1 binding, showing a significant decrease compared to the DMB-BC group.

## Discussion

Previous studies have firmly established the pivotal role of the gut microbiota in the pathogenesis of various diseases^38–40^ The intestinal microenvironment is dynamically regulated by multiple factors, including dietary patterns, hepatic and renal function, and age-related physiological changes^41^. Among these, aging induces profound structural and functional remodeling of the gut microbiota, characterized by the enrichment of Ruminococcaceae family bacteria and increased abundance of opportunistic pathogens such as Klebsiella, Streptococcus, Enterobacter, and Escherichia coli^42,43^. Concurrently, aging is associated with elevated levels of gut microbiota-derived metabolites, including isothiamine, TMAO, and lipopolysaccharide (LPS)^43–45^ which exert pleiotropic effects on host health.

Within the gut-brain axis regulatory network, TMAO has emerged as a key mediator that has garnered substantial research attention. Clinical evidence demonstrates that peripherally circulating TMAO can cross the blood–brain barrier (BBB) to enter the central nervous system (CNS)—a finding further corroborated by its detection in human cerebrospinal fluid (CSF)^46–49^. Notably, TMAO levels increase with advancing age and exhibit a positive correlation with the severity of cognitive impairment^50,51^. Mechanistically, TMAO not only impairs BBB integrity by downregulating tight junction proteins^49^ but also activates multiple pro-inflammatory signaling pathways, including PERK phosphorylation, the NLRP3 inflammasome, and the MAPK/NF-κB axis. These events collectively promote the secretion of pro-inflammatory cytokines such as TNF-α, IL-1β, and IL-18, thereby triggering a robust neuroinflammatory cascade^52–55^.

In our study, we observed that TMAO intervention exacerbated the inflammatory response (Fig. 4), ultimately leading to aggravated cognitive impairment in rats (Fig. 2). Therefore, we hypothesize that the intestinal metabolite TMAO may contribute to age-related cognitive decline possibly by exacerbating neuroinflammation.

Our study revealed an increase in the expression of the NLRP3 inflammasome through the detection of NLRP3 inflammasome-related proteins in hippocampal tissues via Western blot analysis. Previous research has indicated a heightened susceptibility to diverse diseases upon classical activation of the NLRP3 inflammasome. The NLRP3 inflammasome is activated in the intestine of mice with various diseases and plays a crucial role in regulating intestinal homeostasis, including ulcerative colitis^56^, depressive disorder^57^, diabetes^58^, and cardiovascular disease^59^, among others. In central nervous system disorders, activation of the NLRP3 inflammasome has been demonstrated to disrupt the integrity of the intestinal mucosal barrier, trigger inflammatory responses both locally and systemically, subsequently leading to neuroinflammation within the central nervous system and ultimately resulting in cognitive impairment^60–62^. Our study findings confirm that both aging and TMAO intervention are associated with an increased expression of the NLRP3 inflammasome. This process involves a series of consecutive activation steps mediated by inflammatory cytokines IL-1β and IL-18, ultimately leading to exacerbated cognitive impairment in rats.

Exercise, as a non-pharmacological intervention, not only aids in muscle development and physical function enhancement but also contributes to the improvement of sleep quality, appetite regulation, mood elevation, and the promotion of learning and memory. Our study has demonstrated that aging is associated with an increase in intestinal metabolite TMAO levels, which further exacerbates inflammation within the central nervous system and contributes to cognitive decline pathology. Exercise exerts a neuroprotective effect by reducing the production of detrimental metabolites. Previous research has indicated alterations in plasma levels of intestinal microbiota metabolites following exercise training. For instance, moderate to high-intensity physical activity has been linked to decreased TMAO levels^10^. Male volunteers who engaged in exercise exhibited lower plasma TMAO concentrations compared to sedentary obese mice; this was accompanied by inhibition of myocardial inflammation and fibrosis^11^. Obese adults displayed significantly reduced plasma TMAO levels after 12 weeks of exercise intervention when compared to baseline measurements^63^. Studies have also revealed that exercise can downregulate protein expression of IL-1β and IL-18 through inhibition of NF-κB and NLRP3-mediated inflammatory signaling pathways^64^. In an experiment involving rats with post-stroke cognitive impairment undergoing exercise training, beneficial bacteria content increased while protein expression of the NLRP3 inflammasome decreased within brain tissue; subsequently leading to improved cognitive impairment after stroke^65^. Our experimental findings indicate that exercise training not only delays the age-related decline in cognitive function but also inhibits the production of the intestinal metabolite TMAO.

In conclusion, we assessed the D-galactose-induced aging model in rats through NOR, MWM, and RAM experiments. Our study findings suggest that exercise training can effectively delay cognitive decline in rats. Additionally, it reduces the levels of IL-1β and IL-18 in both the hippocampus and plasma following exercise. Furthermore, exercise training appears to reverse the neuroinflammatory response that is exacerbated by cognitive decline and TMAO. These results suggest that exercise training may inhibit the TXNIP-NLRP3-Caspase-1-GSDMD signaling pathway.

Cellular-level investigations confirmed that TXNIP overexpression in HT22 cells substantially increased SPiDER-β-gal-positive cell proportion, initiated NLRP3 inflammasome assembly, and TMAO treatment intensified senescence and inflammatory responses. Conversely, TXNIP knockdown demonstrated opposite effects (Fig. 7). Notably, TXNIP exhibited dual regulatory functions: under normal physiological conditions, its dynamic binding to Trx1 maintained redox homeostasis^66^. This study revealed that TMAO stimulation induced abnormally enhanced TXNIP-Trx1 binding, simultaneously activating senescence and inflammatory signals (Fig. 8A, B). Modification of disulfide bond formation at the critical cysteine residue Cys32 between TXNIP and Trx1 reduced TXNIP’s negative regulation of Trx1 redox activity. However, TMAO treatment enhanced TXNIP-Trx1 binding (Fig. 8C, D). Research indicates that this high-affinity binding state significantly inhibits Trx1 disulfide reductase activity, resulting in decreased cellular antioxidant defense, persistent oxidative damage, and enhanced inflammatory responses^67^. This metabolite-oxidative stress-inflammation cascade presents a novel molecular framework for explaining aging-related cognitive decline.

This study presents several limitations. D-galactose-induced aging primarily operates through mechanisms such as oxidative stress and advanced glycation end product (AGE) formation, which differs from the complex multi-factorial and multi-pathway alterations inherent in natural aging. For instance, natural aging involves diverse mechanisms including telomere shortening and epigenetic modifications, whereas the D-galactose model predominantly focuses on oxidative damage and metabolic dysregulation, failing to fully recapitulate the comprehensive characteristics of natural aging. The investigation primarily focused on detecting changes in the target intestinal metabolite TMAO in senescence model rats, while comprehensive analysis of gut microbiota changes remained insufficient. Future research should employ metagenomic sequencing techniques to examine compositional and functional changes in gut microbiota of senescence model rats, and thoroughly investigate metabolic characteristics of individual microbial taxa and their neural effects. Furthermore, this study did not address functional changes in the intestinal mucosal barrier or blood–brain barrier. Transmission electron microscopy observation of these barriers’ ultrastructure could provide direct imaging evidence. Among numerous studies, this research has not elucidated the specific signaling pathways involved in TMAO-induced TXNIP-Trx1 disulfide bond formation, necessitating further validation through kinase inhibitor experiments in cellular models.

## Conclusion

This study demonstrates that exercise training confers neuroprotective effects by mitigating the exacerbated neuroinflammation in the hippocampal region of D-galactose-induced aging rats. This is achieved through the suppression of the gut metabolite TMAO and the subsequent downregulation of the TXNIP-NLRP3-Caspase-1-GSDMD inflammatory pathway. Consequently, exercise effectively delays the progression of cognitive decline.

## Supplementary Information

Below is the link to the electronic supplementary material.

Supplementary Material 1

Supplementary Material 2

Supplementary Material 3

Supplementary Material 4

## Data Availability

The data used and analyzed during the current study are available from the corresponding author upon reasonable request.

## Abbreviations

TMAO: Trimethylamine N-oxide
TMA: Trimethylamine
NOR: New object recognition
MWM: Morris water maze
RAM: Radial arm maze
WB: Western blotting
SPiDER-β-gal: SPiDER-β-galactosidase
NMDA: N-methyl-D-aspartate
NF-kB: Nuclear factor-kB
FMOs: Flavone monooxygenases
NLRP3: NOD-like receptor protein 3
GSDMD: Gasdermin family D
IL-18: Interleukin-18
IL-1β: Interleukin-1β
AD: Alzheimer’s disease
ROS: Oxidative stress
TXNIP: Thioredoxin-interacting protein
Trx: Thioredoxin
SPF: Specific pathogen-free
UHPLC-MS/MS: Ultra-high performance liquid chromatography-tandem mass spectrometry
ELISA: Enzyme-linked immunosorbent assay
RT-qPCR: Reverse transcription quantitative polymerase chain reaction
Co-IP: Co-immunoprecipitation
LPS: Lipopolysaccharide
BBB: Blood-brain barrier
CNS: Central nervous system
CSF: Cerebrospinal fluid
