# Supplementary material for: Exercise training mitigates age-related cognitive decline by attenuating TMAO-induced inflammation
Source: Sci Rep. 2026 Jan 20;16:5838. doi: 10.1038/s41598-026-36354-z (PMC12894758; doi:10.1038/s41598-026-36354-z)
Supplement: Supplementary file 1 — Supplementary Material 1 [file 41598_2026_36354_MOESM1_ESM.docx]

**Behavioral Tests**

**The new object recognition test (NOR)**

There are three period: familiarization, training testing. Adaptation stage: No objects were introduced into the box, and rats were placed inside to freely explore the environment for 5 minutes while maintaining a quiet setting, allowing them to acclimate to their surroundings. The adaptation period lasted for 3 days. Familiarization stage: Two identical objects, denoted as A and B, were positioned at opposite ends of one side arm within the identification box. Ensuring a distance of 15cm between each object and the side wall enabled unrestricted movement for the rats along this pathway. Subsequently, the rats were placed in the box under quiet conditions and allowed to freely explore for 5 minutes before being returned to their cages. Test stage: One of the objects was replaced with a novel object (object C), while maintaining its original position alongside object A. Following an hour of rest, rats were reintroduced into the same location and given another opportunity to explore freely for 5 minutes. During this phase, video recording equipment was utilized to document each rat's exploration time spent on both new object C and familiar object A. Exploration behavior encompassed sniffing or touching objects within a proximity of 2 cm; climbing atop an object did not qualify as exploratory behavior. Throughout these three stages, prior to testing each rat, strict disinfection using 75% alcohol was performed on the box. Discrimination index = (time spent exploring object C- time spent exploring object A) / (time spent exploring object A + time spent exploring object C).

**Morris water maze test (MWM)**

The MWM experiment consists of two main components: a navigation test and a spatial exploration test. The hardware equipment used in the water maze experiment is a circular pool with dimensions of 150 cm in diameter, 50 cm in height, and 30 cm in depth. The pool is divided into four quadrants, each marked with recognizable symbols on the inner wall. A circular survival platform with an adjustable height of 12 cm is fixed within the first quadrant. To ensure that rats can climb onto the platform, water is filled above it by at least 2 cm while maintaining a temperature of 24 ± 2 °C. Black ink is added to dye the water black, creating a distinct color contrast between the platform and rats for visibility purposes. The pool contains four quadrants and eight designated entry positions labeled as numbers from one to eight (1-8). Each mouse undergoes four daily experiments randomly assigned to positions two, three, seven, or eight (the order may vary). When placing rats into the pool, they are gently introduced facing its wall to prevent choking on water. Rats are allowed one minute for free exploration, if they fail to find the platform within this time frame, they are guided towards it and remain there for fifteen seconds. This experimental procedure lasts for five days while recording escape latency. On the sixth day, the platform was removed while keeping all other external conditions unchanged. Subsequently, the rats were positioned at position 5 (the furthest location from the target platform that had not been used during the navigation experiment) and allowed to freely explore in water for a duration of 1 minute. The time spent in the target quadrant and the times crossing the targeted quadrant were recorded. The collected data underwent processing and statistical analysis using an animal video tracking analysis system.

**Radial arm maze (RAM)**

The fundamental working principle of the RAM experiment lies in the animal's ability to effectively discern the arm where food is placed by utilizing location information provided by room markers, thereby facilitating the investigation of spatial working memory and reference memory in rats. Adaptation to the training environment involves a 12-hour fasting period after weighing. Food is positioned at the extremity of each arm within the RAM, ensuring that it lacks taste as much as possible and adheres to specifications, allowing rats to consume it in one mouthful. During testing, rats are situated on the central platform of the maze with all eight side doors opened, enabling them to freely explore without human interference and minimizing external factors during their exploration process. Training sessions are conducted twice daily for 5 minutes each over a span of 3 days. Prior to each session, rats are placed on the central platform with all eight arm doors open, granting them unrestricted movement throughout the maze without any intervention or constraints. raining and testing stage: Four arms (arms 1, 3, 5, and 7) were randomly selected to place one food item at the end of each arm for animals to choose from. Throughout the experiment, the feeding locations in arms 1, 3, 5, and 7 remained unchanged. Prior to testing, rats underwent a fasting period of twelve hours with closed arms while being placed on the central platform area. After thirty seconds, the arms were opened, and rats were allowed free access to feed in the maze. The experiment concluded when all four food items were consumed within five minutes; if not consumed within this time frame, a five-minute exploration period was permitted before ending the experiment. Training sessions occurred twice daily for five minutes each over seven days, with testing conducted on the final day. Data processing and statistical analysis were performed using an animal video tracking analysis system. Correct choices of arms containing food and successful arrival at food locations were recorded as correct choices (scored as '1'). Recorded indicators included: working memory errors, instances where animals repeatedly entered previously visited arms that had already been fed during training; the frequency of working memory errors was calculated by dividing total working memory errors by the total number of arm entries. Reference memory errors, instances where animals entered arms without any food present; the frequency of reference memory errors was calculated by dividing total reference memory errors by the total number of arm entries.
