# Supplementary material for: Exercise training mitigates age-related cognitive decline by attenuating TMAO-induced inflammation
Source: Sci Rep. 2026 Jan 20;16:5838. doi: 10.1038/s41598-026-36354-z (PMC12894758; doi:10.1038/s41598-026-36354-z)

Fig. 4

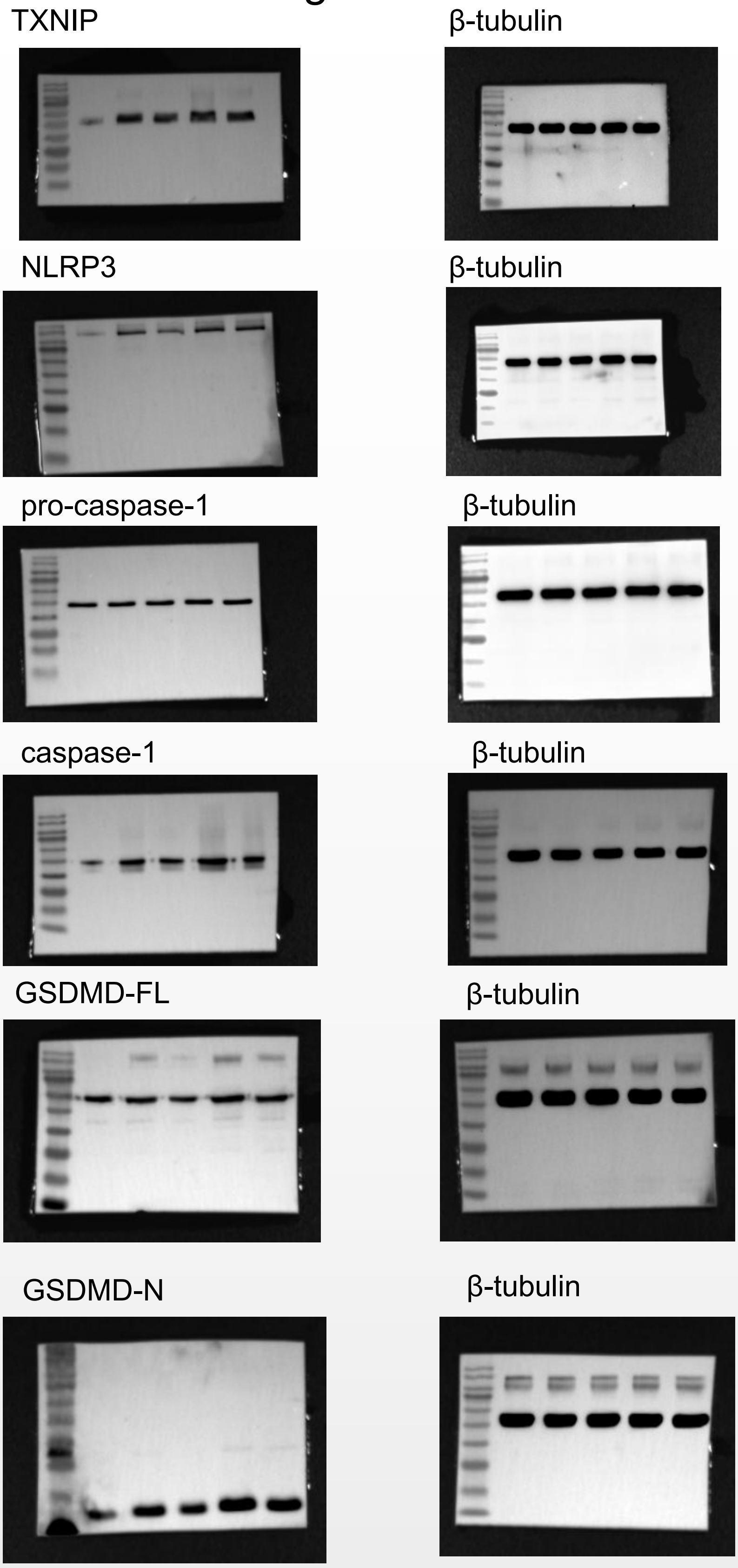

TXNIP
β-tubulin
NLRP3
β-tubulin
pro-caspase-1
β-tubulin
caspase-1
β-tubulin
GSDMD-FL
β-tubulin
GSDMD-N
β-tubulin

Fig. 5

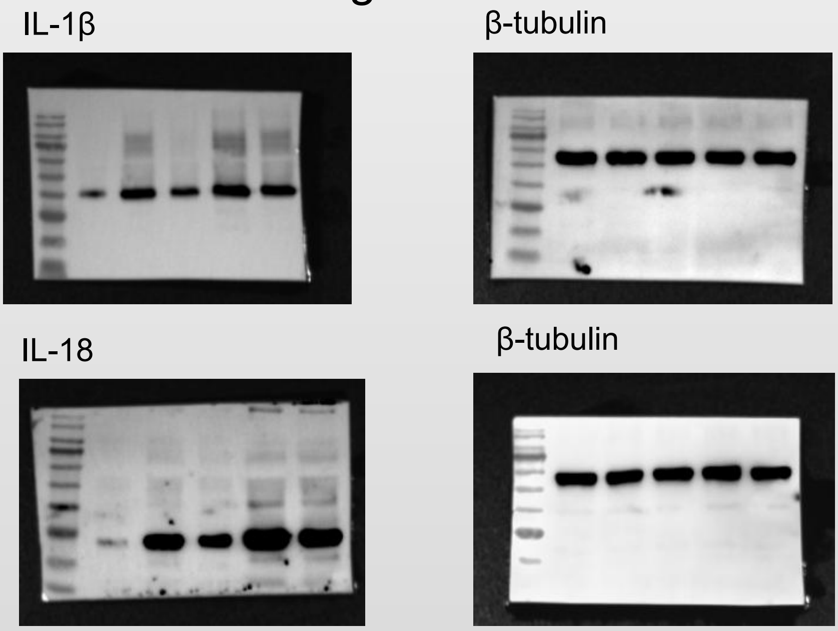

IL-1β
β-tubulin
IL-18
β-tubulin

Fig. 6

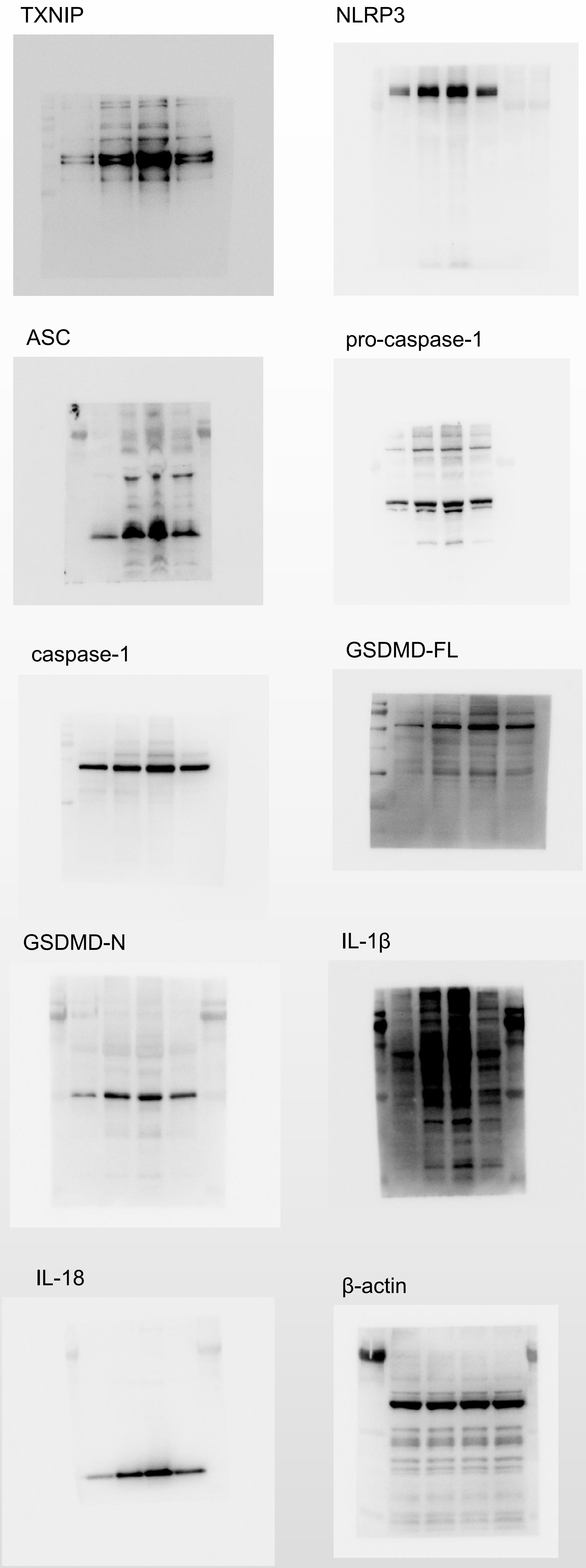
TXNIP
NLRP3
ASC
pro-caspase-1
caspase-1
GSDMD-FL
GSDMD-N
IL-1β
IL-18
β-actin

Fig. 7

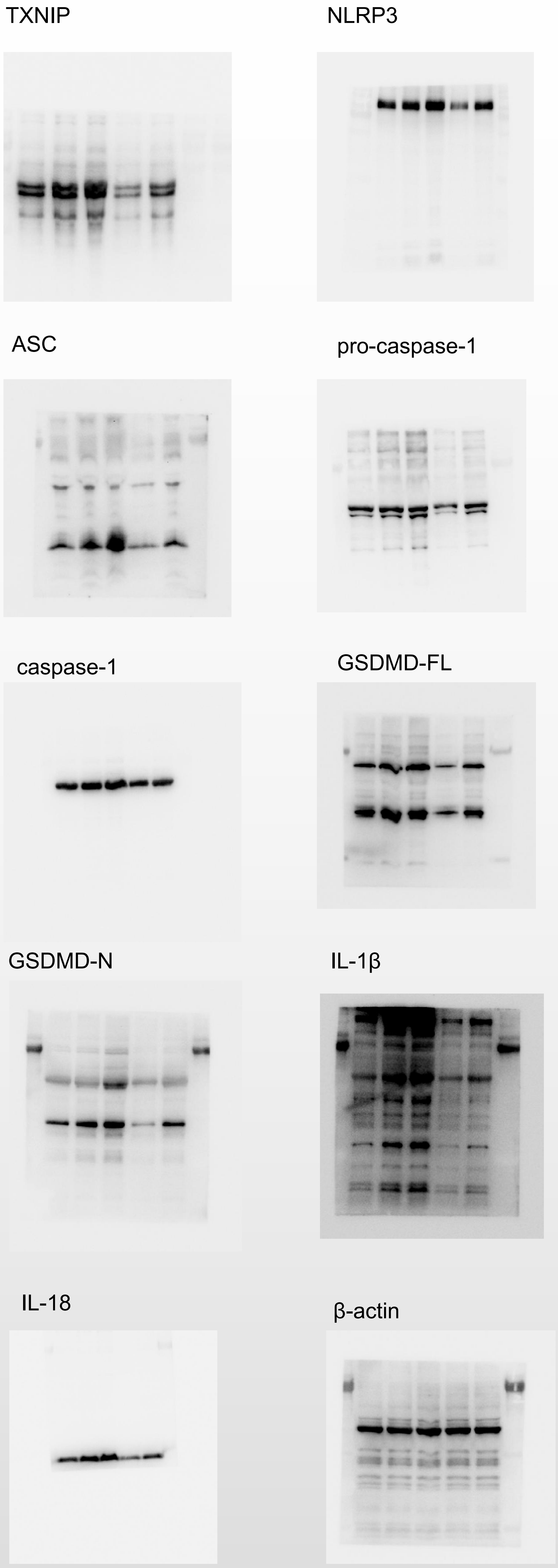
TXNIP
NLRP3
ASC
pro-caspase-1
caspase-1
GSDMD-FL
GSDMD-N
IL-1β
IL-18
β-actin

Fig. 8

Fig. 8A Trx1-1

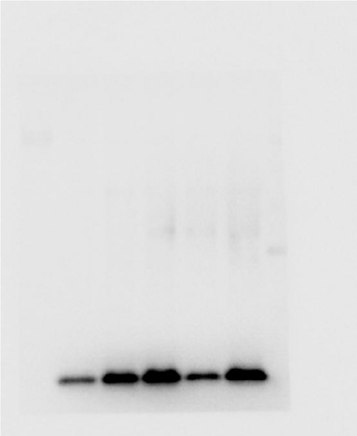

Fig. 8A Trx1-2

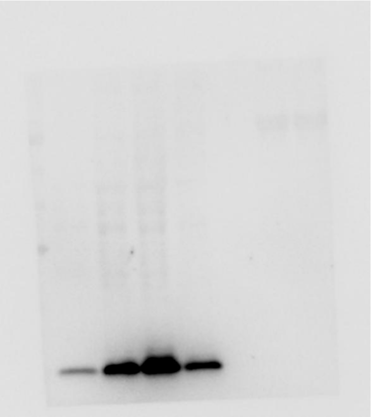

Fig. 8A TXNIP-1

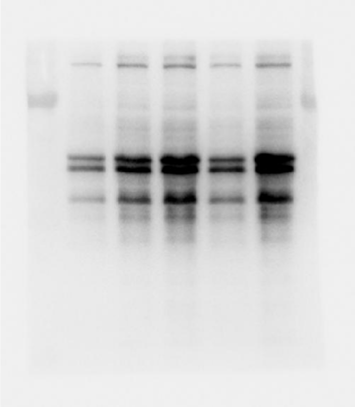

Fig. 8A TXNIP-2

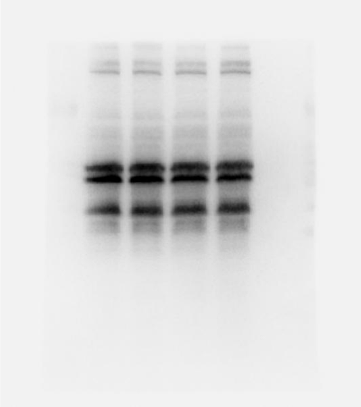

Fig. 8A β-actin

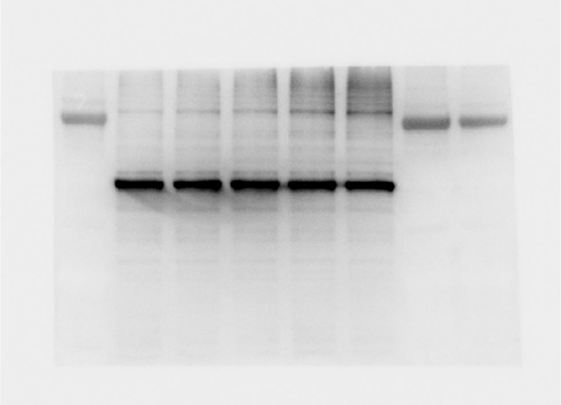

Fig. 8C Trx1-1

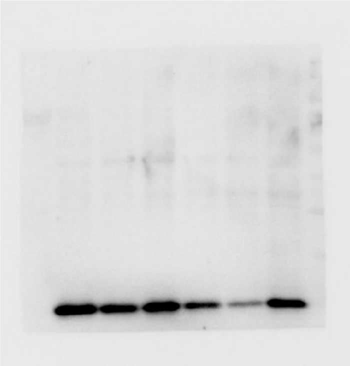

Fig. 8C Trx1-2

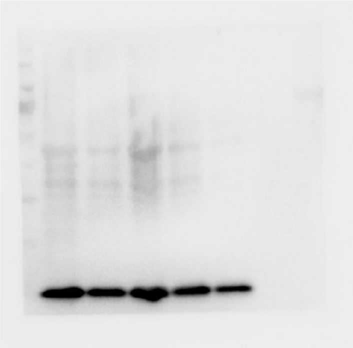

Fig. 8C TXNIP-1

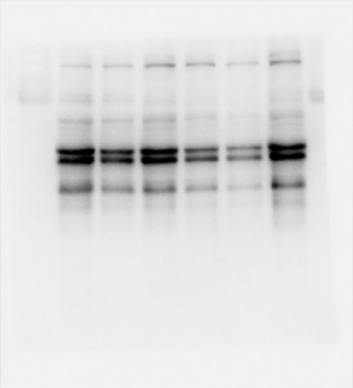

Fig. 8C TXNIP-2

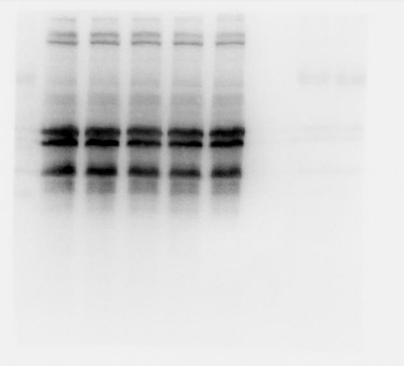

Fig. 8C β-actin

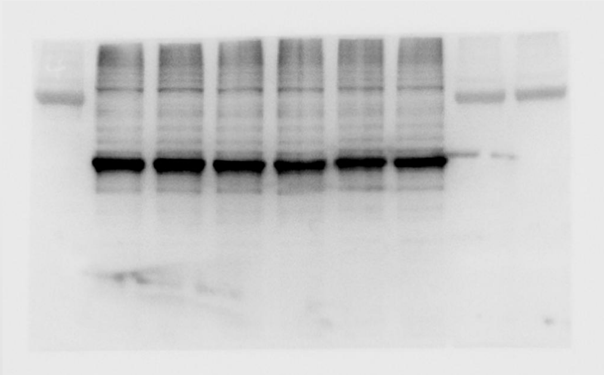

Supplement: Supplementary file 2 — Supplementary Material 2 [file 41598_2026_36354_MOESM2_ESM.pdf]
