# Supplementary material for: Exercise training mitigates age-related cognitive decline by attenuating TMAO-induced inflammation
Source: Sci Rep. 2026 Jan 20;16:5838. doi: 10.1038/s41598-026-36354-z (PMC12894758; doi:10.1038/s41598-026-36354-z)
Supplement: Supplementary file 3 — Supplementary Material 3 [file 41598_2026_36354_MOESM3_ESM.pdf]

Term	Definition
pyroptosis	A type of inflammasome-mediated programmed inflammatory cell death that is distinct from apoptosis and necrosis, characterized by lytic cell death accompanied by the release of large amounts of pro-inflammatory cytokines.
Aβ pathology	The pathological feature formed by the abnormal deposition of amyloid-beta (Aβ) is one of the core pathological hallmarks of Alzheimer's disease (AD).
AD	A common neurodegenerative disease characterized by amyloid-beta (Aβ) deposition, excessive tau phosphorylation, and progressive cognitive decline.
APP/PS1 mice	Transgenic mice overexpressing amyloid precursor protein (APP) and presenilin 1 (PS1), which are commonly used as animal models to simulate the pathological progression of Alzheimer's disease (AD).
Caspase-1	A key downstream protease activated by inflammasomes, which can cleave GSDMD to induce pyroptosis and promote the maturation and release of pro-inflammatory cytokines such as IL-1β and IL-18.
FMOs	A family of enzymes mainly expressed in the liver, responsible for oxidizing gut-derived trimethylamine (TMA) to trimethylamine N-oxide (TMAO).
GSDMD	A key executor protein of pyroptosis. After being cleaved by Caspase-1, it forms membrane pores, leading to cell swelling, rupture, and the release of pro-inflammatory cytokines.
IL-1β	An important pro-inflammatory cytokine produced under inflammasome mediation, which participates in neuroinflammatory responses and exacerbates age-related cognitive decline.
IL-18	A pro-inflammatory cytokine that acts synergistically with IL-1β to mediate inflammatory responses and pyroptosis, and promotes cognitive impairment in neurodegenerative diseases.
Microglia	The resident immune cells of the central nervous system (CNS). Upon activation, they can release pro-inflammatory cytokines, and neuroinflammation mediated by their excessive activation is an important mechanism of cognitive decline.
NF-κB	A key inflammatory transcription factor that can regulate the expression of inflammation-related genes such as NLRP3, and plays a central role in TMAO-induced inflammatory responses.
NLRP3	An important member of the inflammasome family, which

	can be activated by oxidative stress and metabolites (e.g., TMAO) to initiate the Caspase-1-GSDMD signaling pathway.
Inflammasome	An intracellular multiprotein complex (e.g., NLRP3) that can sense damage-associated molecular patterns (DAMPs) or pathogen-associated molecular patterns (PAMPs), and activate Caspase-1 to trigger inflammation and pyroptosis.
Nrf2	A key transcription factor for cellular antioxidant stress, which can negatively regulate NLRP3 inflammasome activation by modulating the Trx1/TXNIP complex.
TMA	An intermediate product produced by gut microbiota metabolizing substrates rich in trimethylamine groups. It enters the liver via the portal vein and is oxidized to TMAO.
TMAO	A toxic metabolite derived from gut microbiota metabolism, which can activate the NLRP3 inflammasome to induce pyroptosis and neuroinflammation, and is a risk factor for age-related cognitive decline.
Trx1	An important intracellular antioxidant protein that maintains redox balance through a cysteine thiol-disulfide exchange mechanism. It can be inhibited by TXNIP and participates in the regulation of the NLRP3 inflammasome.
TXNIP	An endogenous inhibitor of Trx1, which can promote the maturation of inflammatory cytokines by activating the NLRP3 inflammasome pathway and plays a key role in oxidative stress and cognitive decline.
Tau phosphorylation	Abnormal excessive phosphorylation of tau protein leads to its aggregation into neurofibrillary tangles (NFTs), which is a core pathological change in neurodegenerative diseases such as AD and is closely associated with cognitive decline.
3×Tg AD mice	Transgenic mice co-expressing mutant APP, PS1, and tau genes, which can simulate the core pathological features (Aβ deposition, tau aggregation) and cognitive impairment of AD.
