# Supplementary figures and images for: Exercise training mitigates age-related cognitive decline by attenuating TMAO-induced inflammation
Source: Sci Rep. 2026 Jan 20;16:5838. doi: 10.1038/s41598-026-36354-z (PMC12894758; doi:10.1038/s41598-026-36354-z)

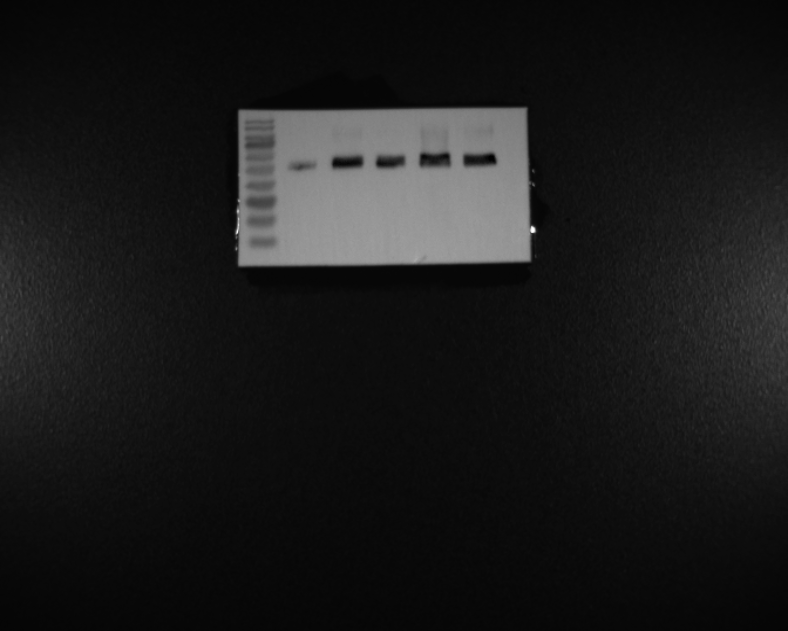

Supplement: Supplementary file 4 — Supplementary Material 4 [file 41598_2026_36354_MOESM4_ESM.zip › Full uncropped Gels and Blots image(s)/Fig.4/Fig.4A-TXNIP.png]

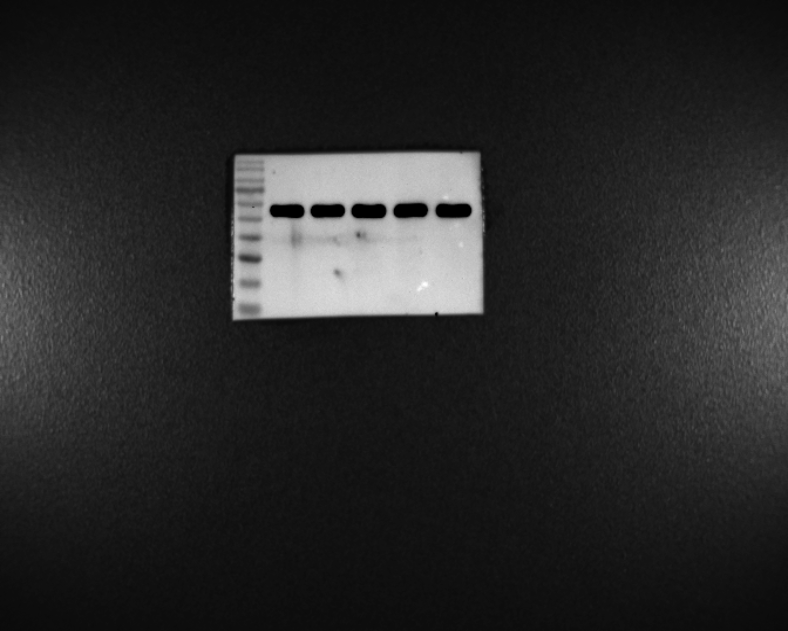

Supplement: Supplementary file 4 — Supplementary Material 4 [file 41598_2026_36354_MOESM4_ESM.zip › Full uncropped Gels and Blots image(s)/Fig.4/Fig.4A-β-tubulin.png]

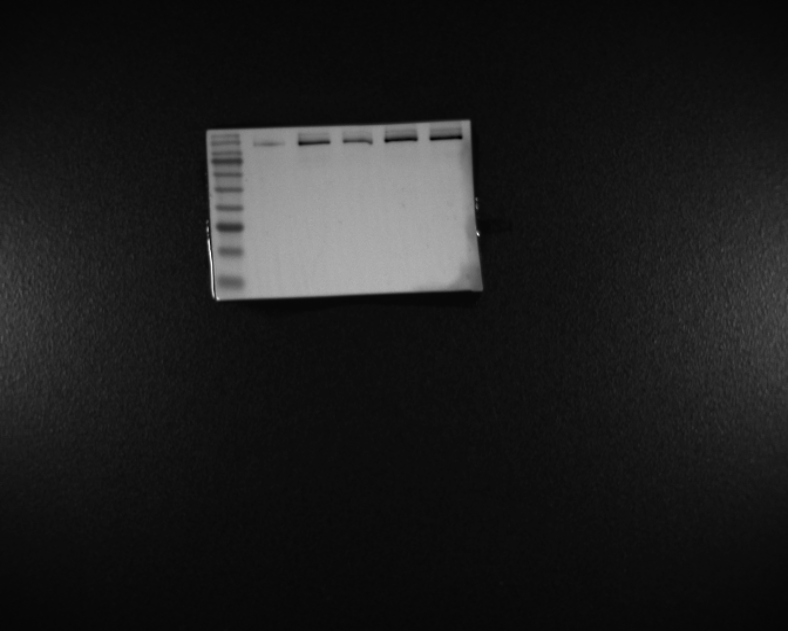

Supplement: Supplementary file 4 — Supplementary Material 4 [file 41598_2026_36354_MOESM4_ESM.zip › Full uncropped Gels and Blots image(s)/Fig.4/Fig.4B-NLRP3.png]

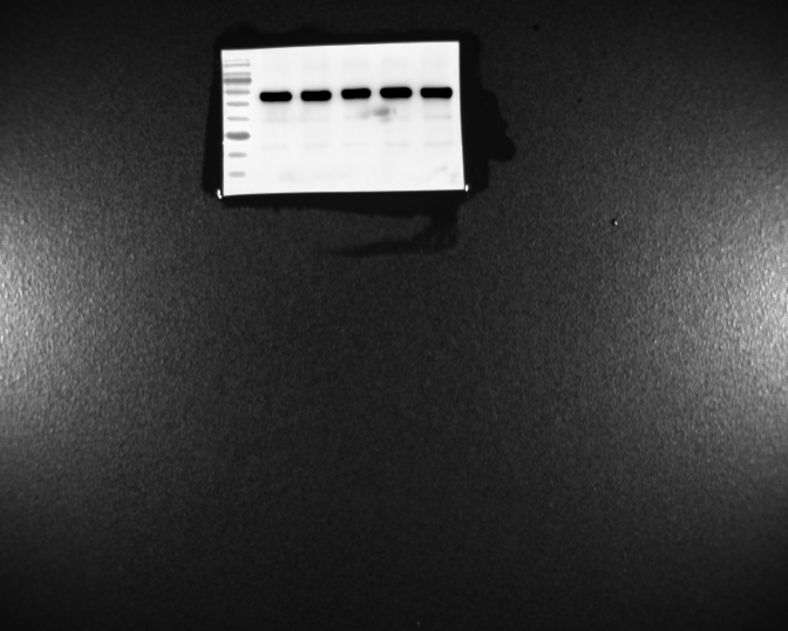

Supplement: Supplementary file 4 — Supplementary Material 4 [file 41598_2026_36354_MOESM4_ESM.zip › Full uncropped Gels and Blots image(s)/Fig.4/Fig.4B-β-tubulin.png]

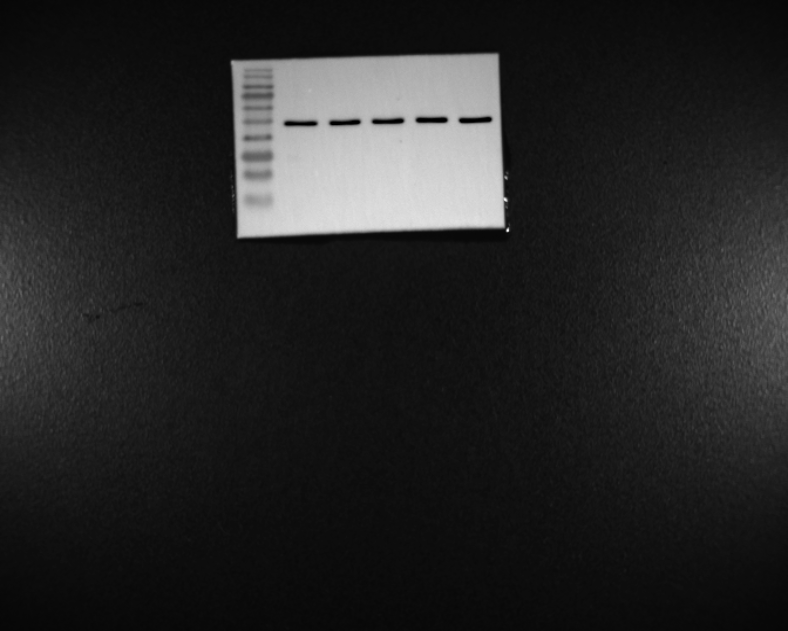

Supplement: Supplementary file 4 — Supplementary Material 4 [file 41598_2026_36354_MOESM4_ESM.zip › Full uncropped Gels and Blots image(s)/Fig.4/Fig.4C-pro-caspase-1.png]

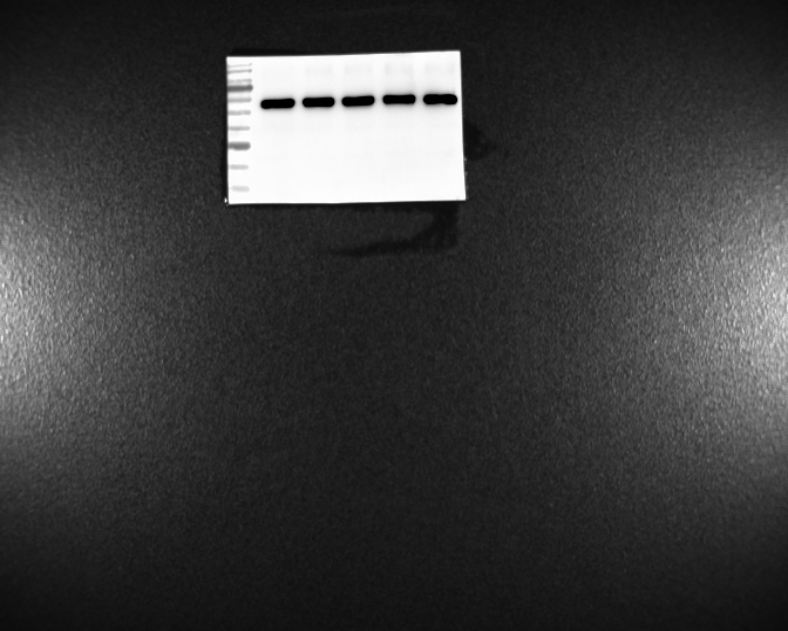

Supplement: Supplementary file 4 — Supplementary Material 4 [file 41598_2026_36354_MOESM4_ESM.zip › Full uncropped Gels and Blots image(s)/Fig.4/Fig.4C-β-tubulin.png]

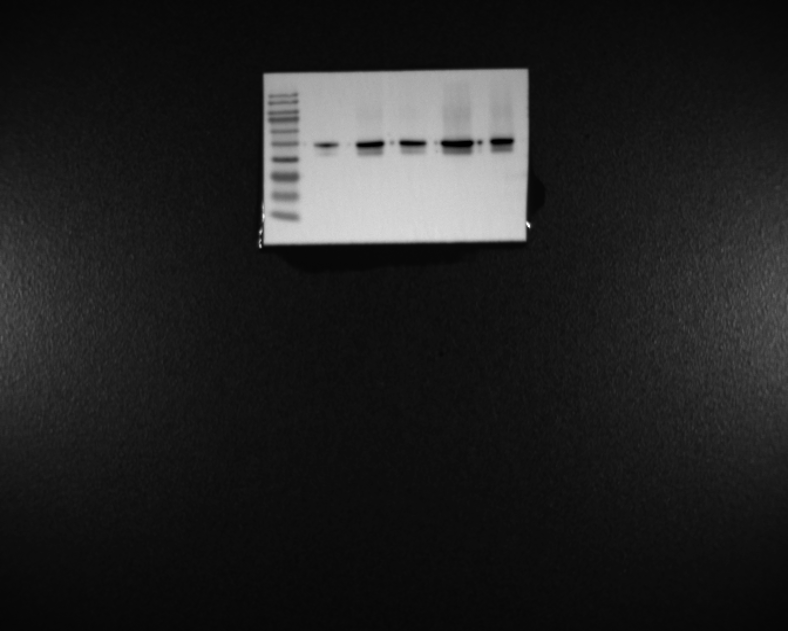

Supplement: Supplementary file 4 — Supplementary Material 4 [file 41598_2026_36354_MOESM4_ESM.zip › Full uncropped Gels and Blots image(s)/Fig.4/Fig.4D-caspase-1.png]

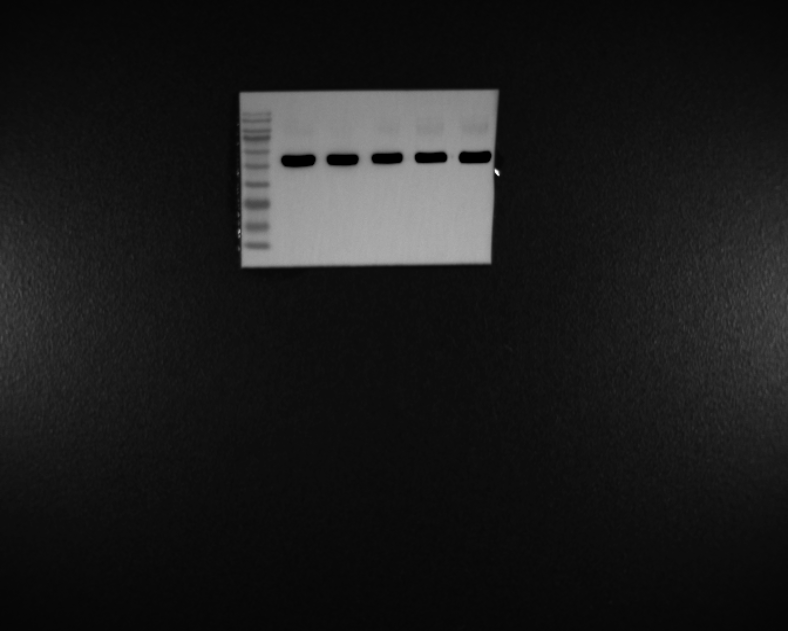

Supplement: Supplementary file 4 — Supplementary Material 4 [file 41598_2026_36354_MOESM4_ESM.zip › Full uncropped Gels and Blots image(s)/Fig.4/Fig.4D-β-tubulin.png]

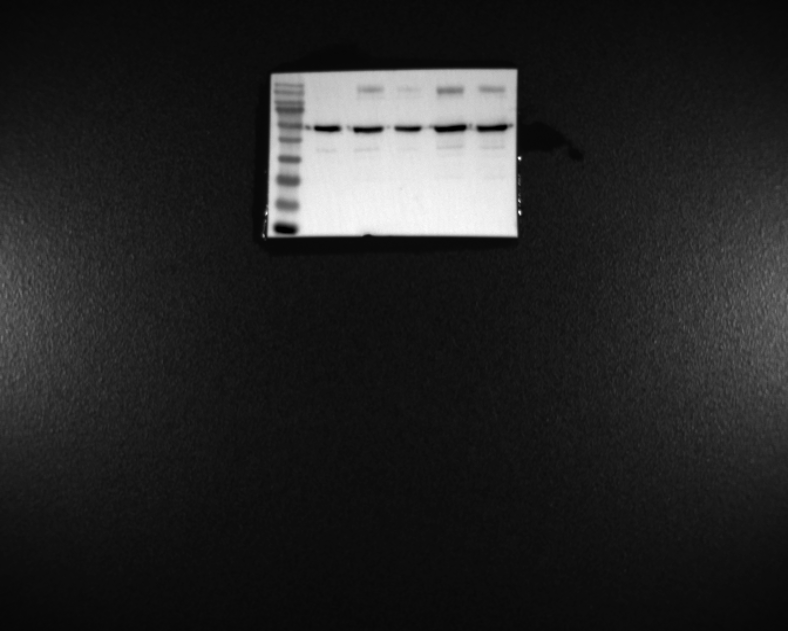

Supplement: Supplementary file 4 — Supplementary Material 4 [file 41598_2026_36354_MOESM4_ESM.zip › Full uncropped Gels and Blots image(s)/Fig.4/Fig.4E-GSDMD-FL.png]

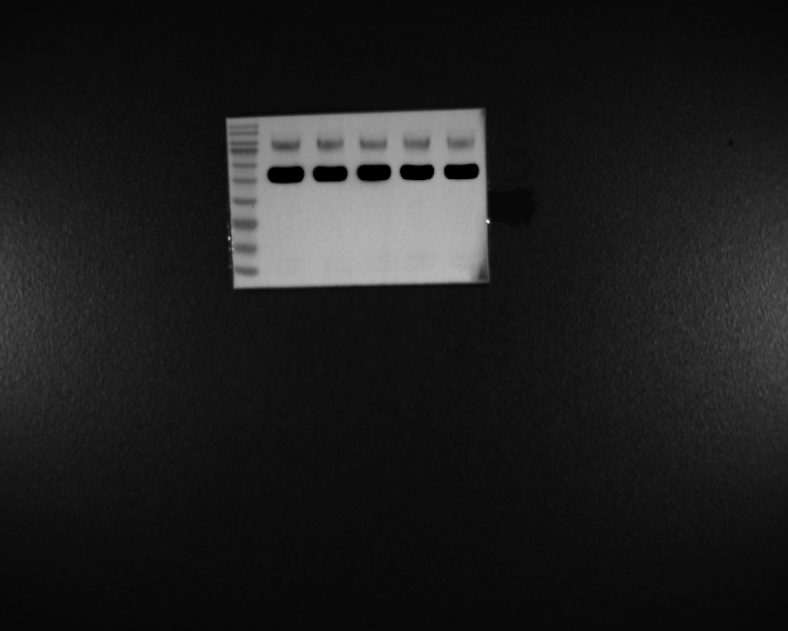

Supplement: Supplementary file 4 — Supplementary Material 4 [file 41598_2026_36354_MOESM4_ESM.zip › Full uncropped Gels and Blots image(s)/Fig.4/Fig.4E-β-tubulin.png]

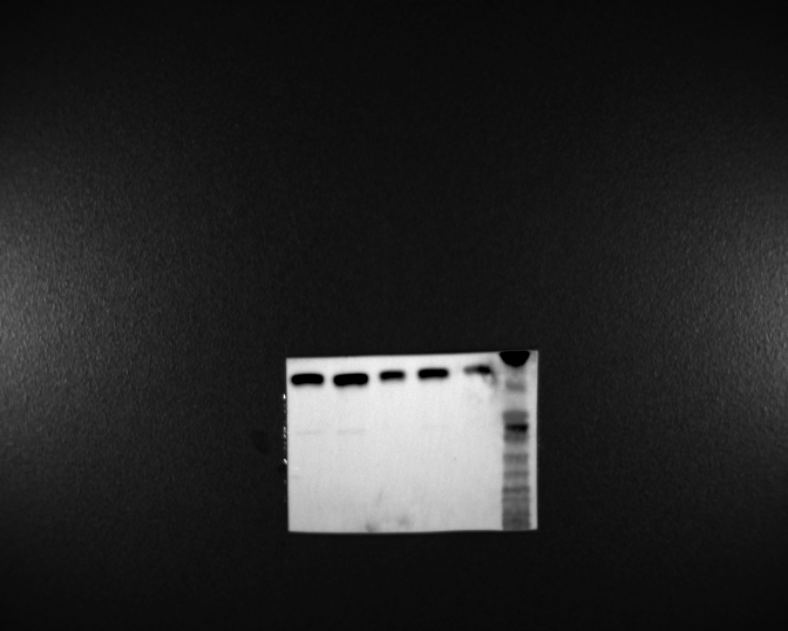

Supplement: Supplementary file 4 — Supplementary Material 4 [file 41598_2026_36354_MOESM4_ESM.zip › Full uncropped Gels and Blots image(s)/Fig.4/Fig.4F-GSDMD-N.png]

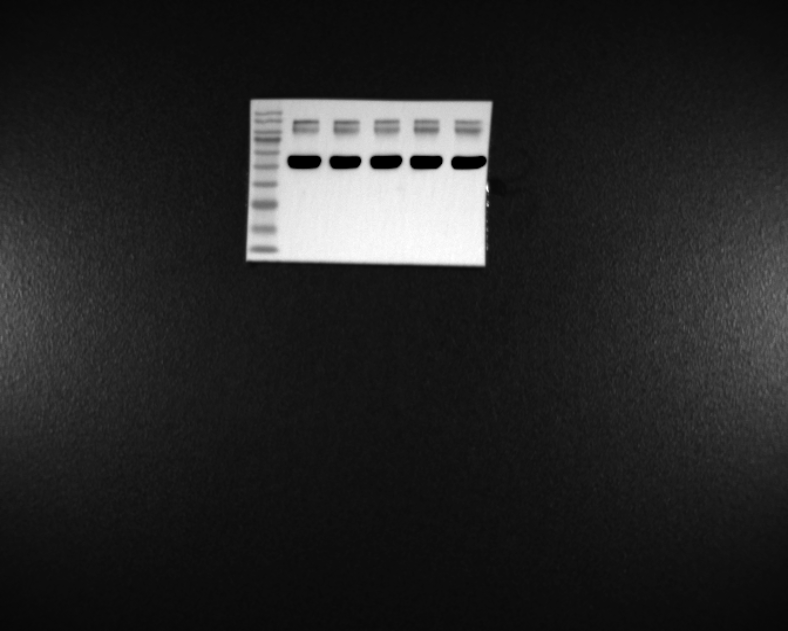

Supplement: Supplementary file 4 — Supplementary Material 4 [file 41598_2026_36354_MOESM4_ESM.zip › Full uncropped Gels and Blots image(s)/Fig.4/Fig.4F-β-tubulin.png]

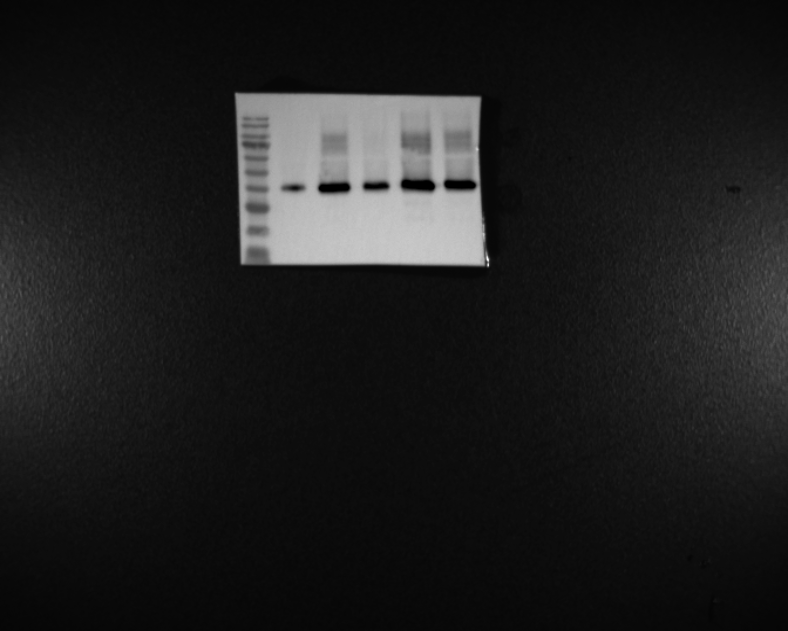

Supplement: Supplementary file 4 — Supplementary Material 4 [file 41598_2026_36354_MOESM4_ESM.zip › Full uncropped Gels and Blots image(s)/Fig.5/Fig.5C-IL-1β.png]

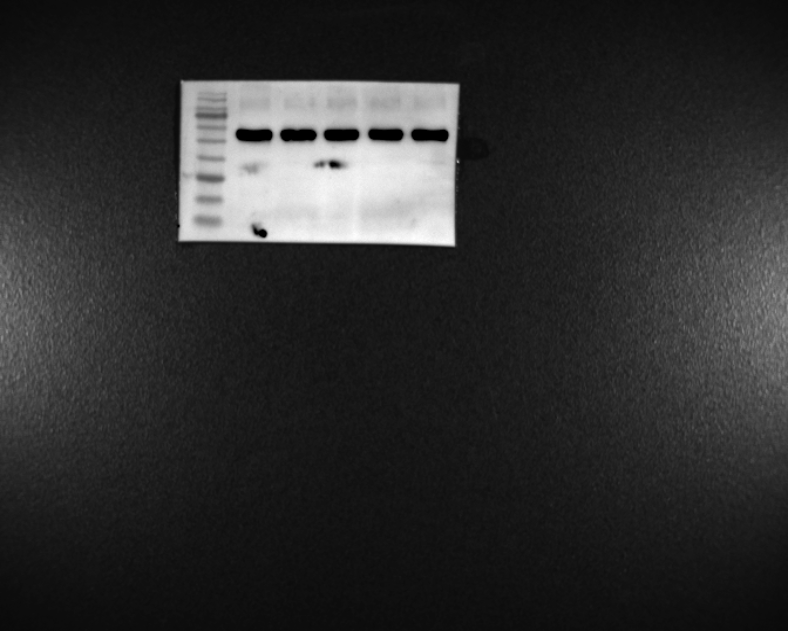

Supplement: Supplementary file 4 — Supplementary Material 4 [file 41598_2026_36354_MOESM4_ESM.zip › Full uncropped Gels and Blots image(s)/Fig.5/Fig.5C-β-tubulin.png]

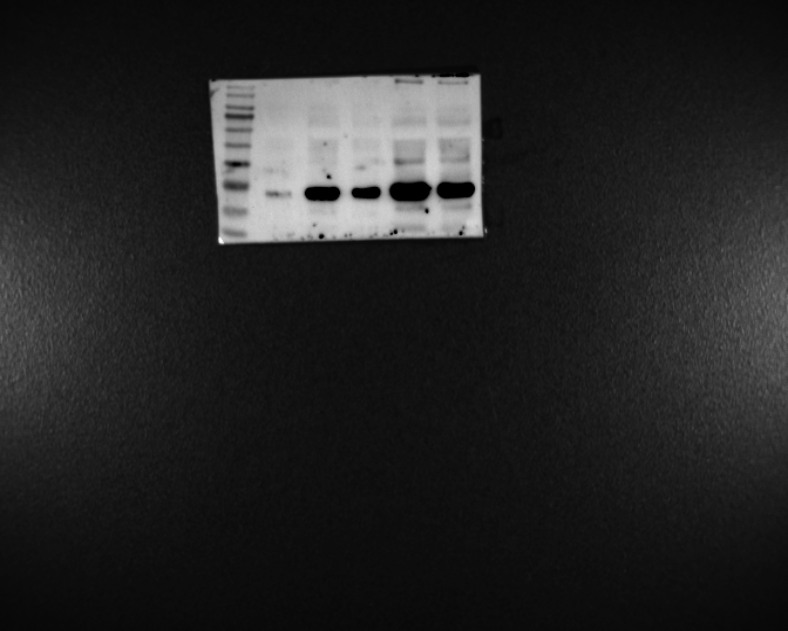

Supplement: Supplementary file 4 — Supplementary Material 4 [file 41598_2026_36354_MOESM4_ESM.zip › Full uncropped Gels and Blots image(s)/Fig.5/Fig.5D-IL-18.png]

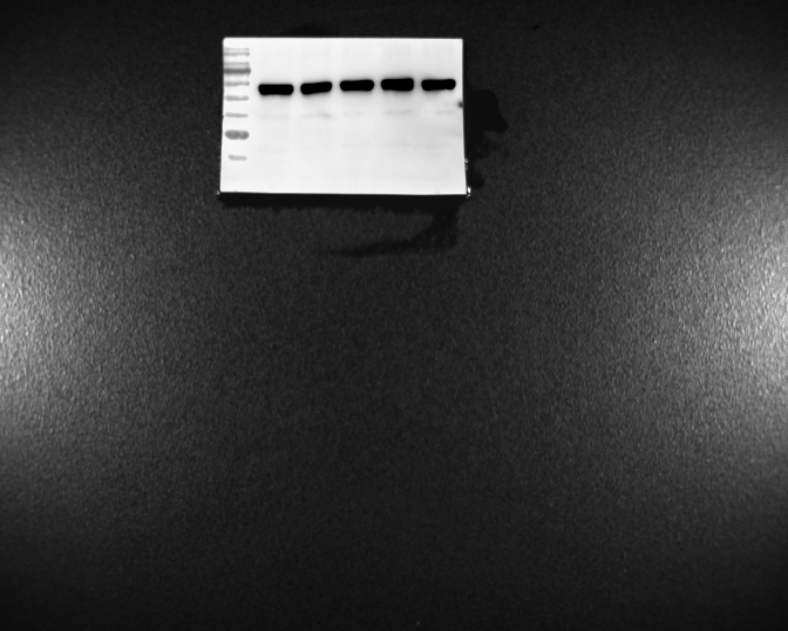

Supplement: Supplementary file 4 — Supplementary Material 4 [file 41598_2026_36354_MOESM4_ESM.zip › Full uncropped Gels and Blots image(s)/Fig.5/Fig.5D-β-tubulin.png]

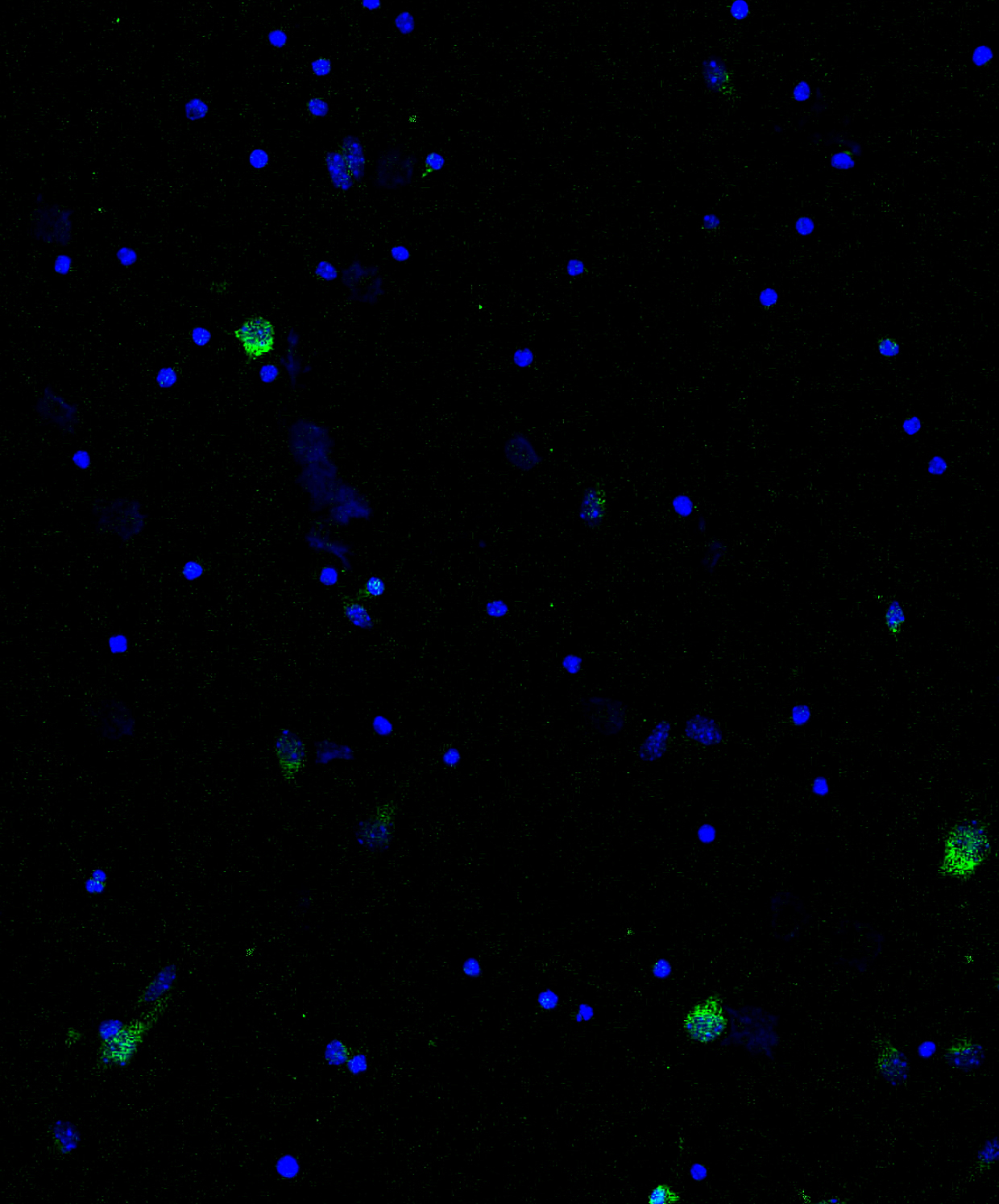

Supplement: Supplementary file 4 — Supplementary Material 4 [file 41598_2026_36354_MOESM4_ESM.zip › Full uncropped Gels and Blots image(s)/Fig.6/Fig.6A/Aging+DMB.jpg]

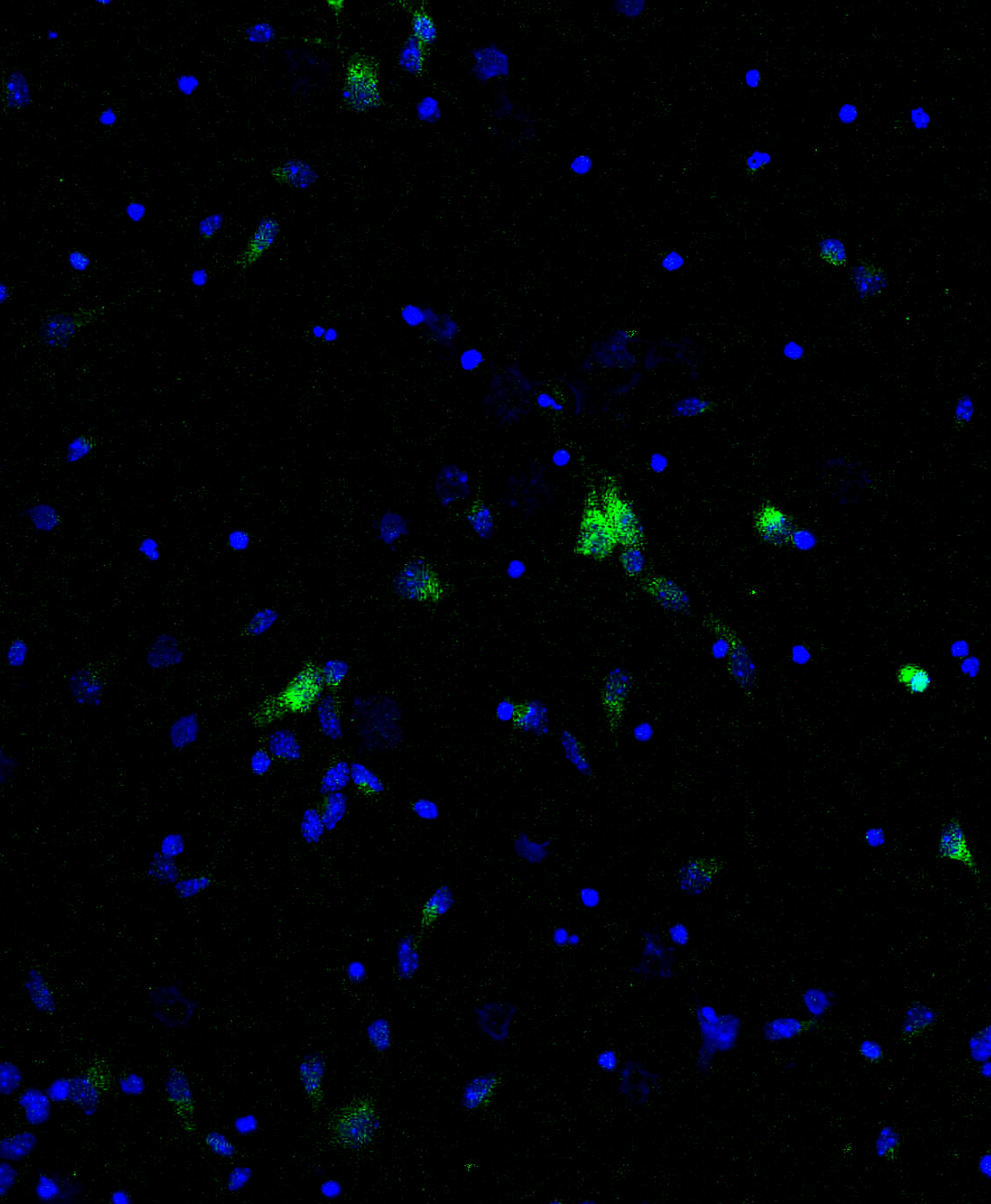

Supplement: Supplementary file 4 — Supplementary Material 4 [file 41598_2026_36354_MOESM4_ESM.zip › Full uncropped Gels and Blots image(s)/Fig.6/Fig.6A/Aging+NS.jpg]

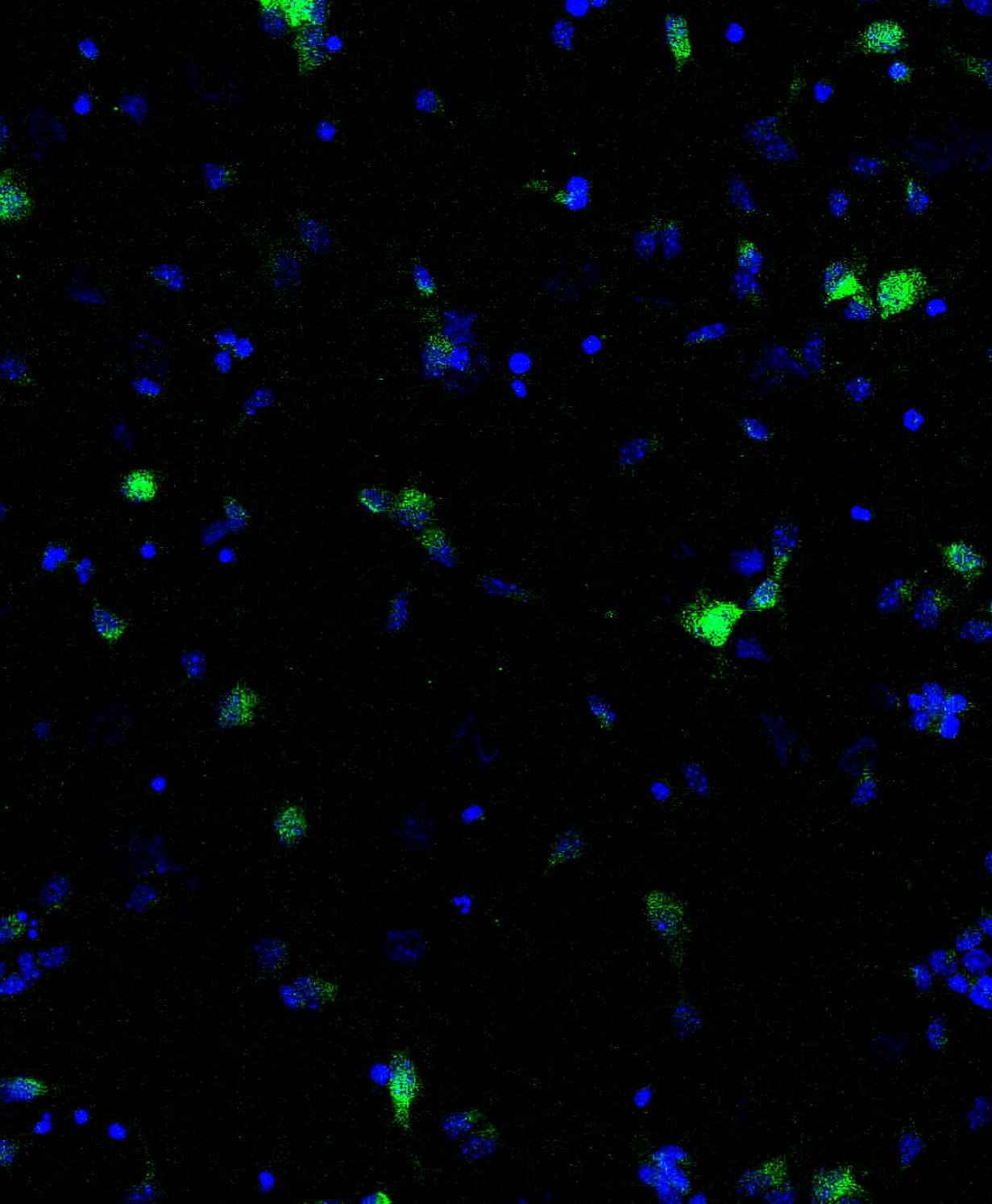

Supplement: Supplementary file 4 — Supplementary Material 4 [file 41598_2026_36354_MOESM4_ESM.zip › Full uncropped Gels and Blots image(s)/Fig.6/Fig.6A/Aging+TMAO.jpg]

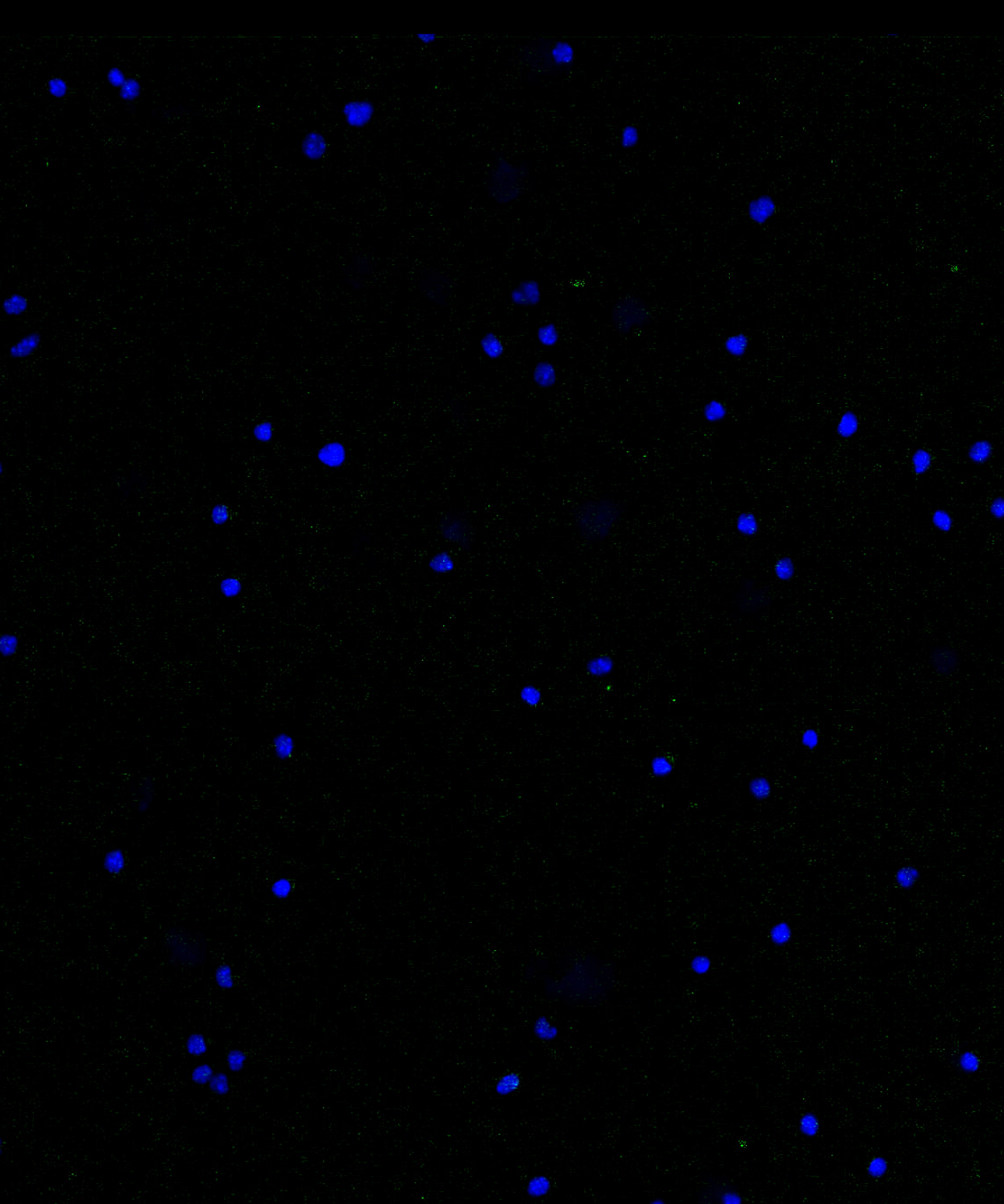

Supplement: Supplementary file 4 — Supplementary Material 4 [file 41598_2026_36354_MOESM4_ESM.zip › Full uncropped Gels and Blots image(s)/Fig.6/Fig.6A/Control.jpg]

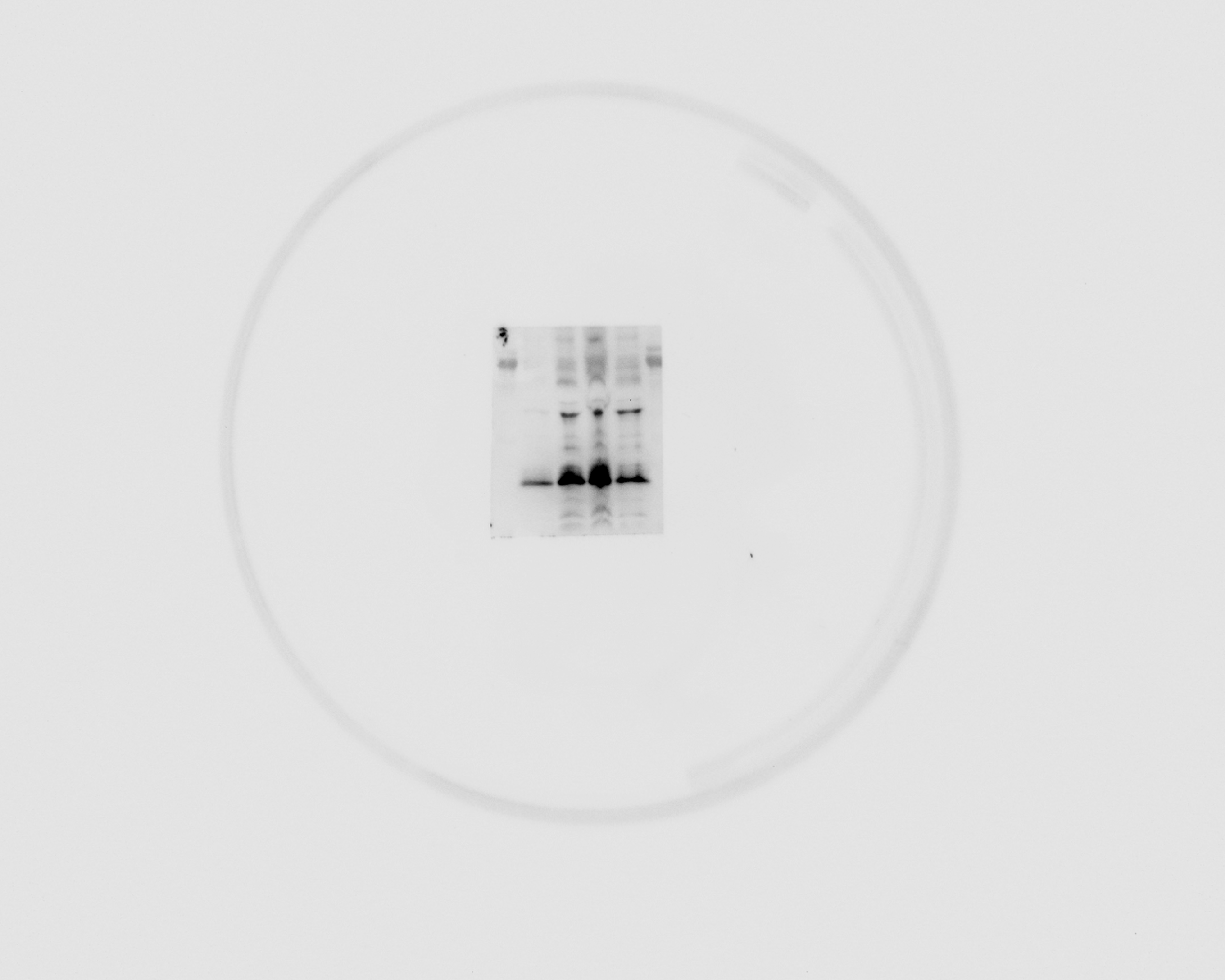

Supplement: Supplementary file 4 — Supplementary Material 4 [file 41598_2026_36354_MOESM4_ESM.zip › Full uncropped Gels and Blots image(s)/Fig.6/Fig.6C/ASC-1.tif]

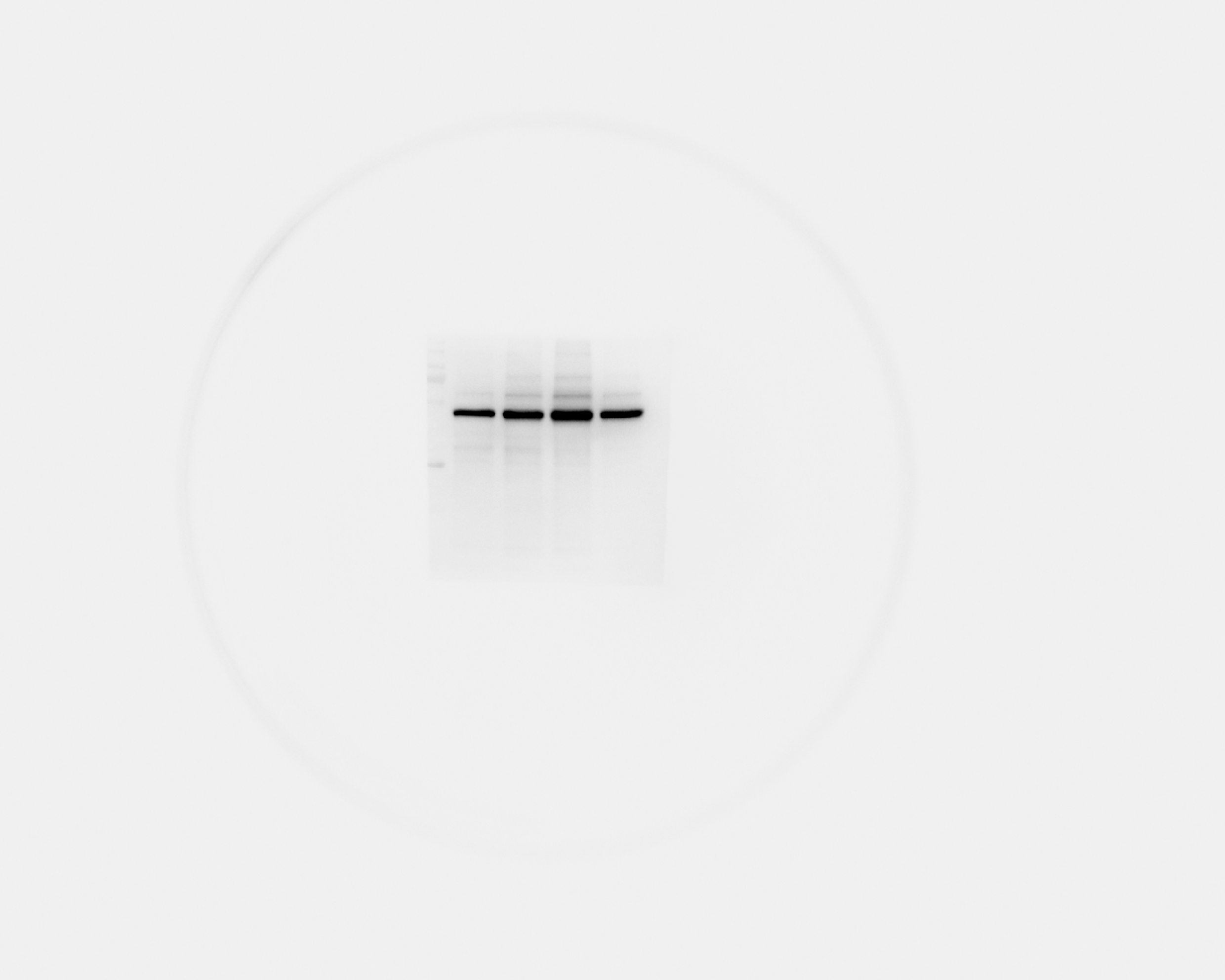

Supplement: Supplementary file 4 — Supplementary Material 4 [file 41598_2026_36354_MOESM4_ESM.zip › Full uncropped Gels and Blots image(s)/Fig.6/Fig.6C/Caspase-1.tif]

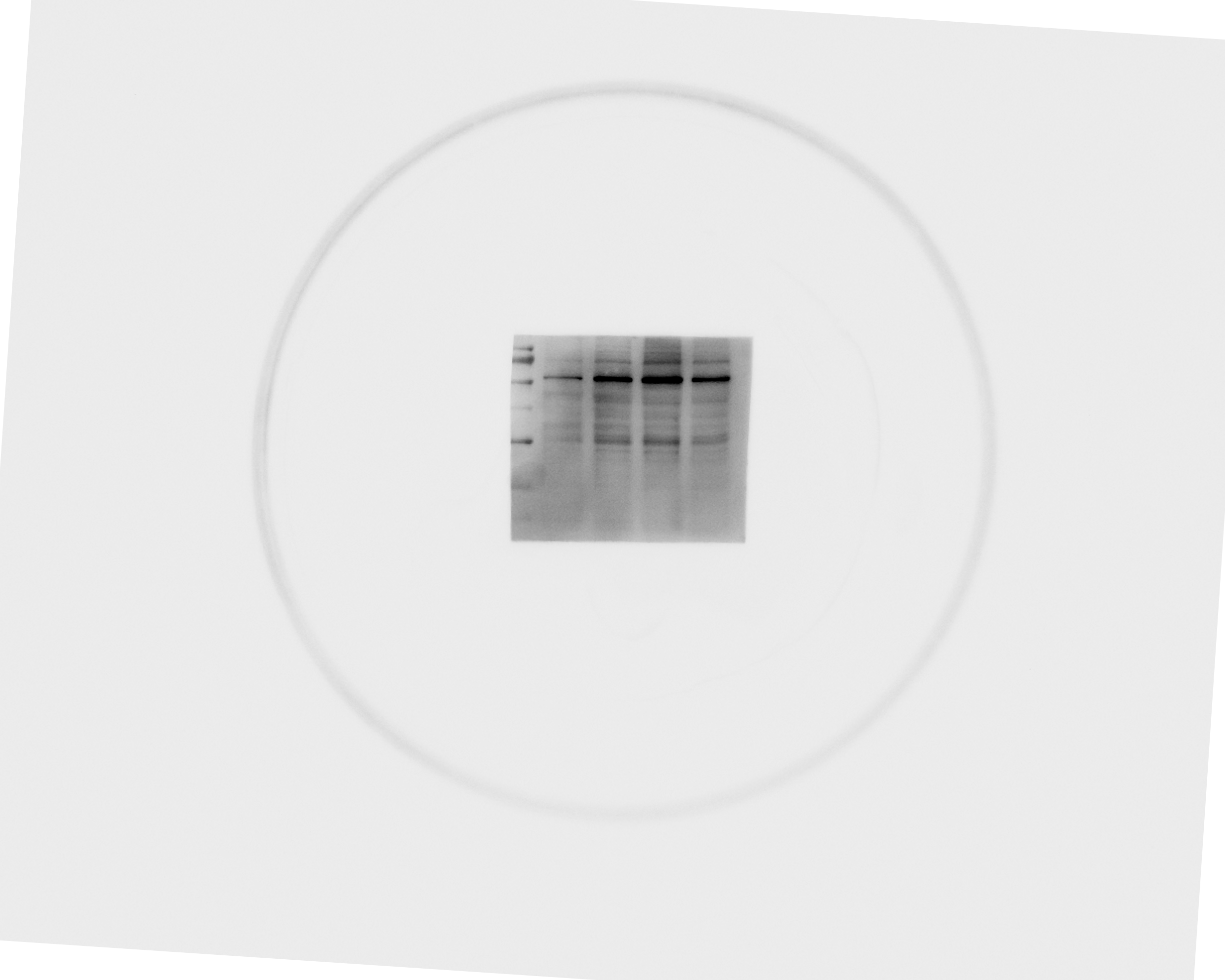

Supplement: Supplementary file 4 — Supplementary Material 4 [file 41598_2026_36354_MOESM4_ESM.zip › Full uncropped Gels and Blots image(s)/Fig.6/Fig.6C/GSDMD-FL.tif]

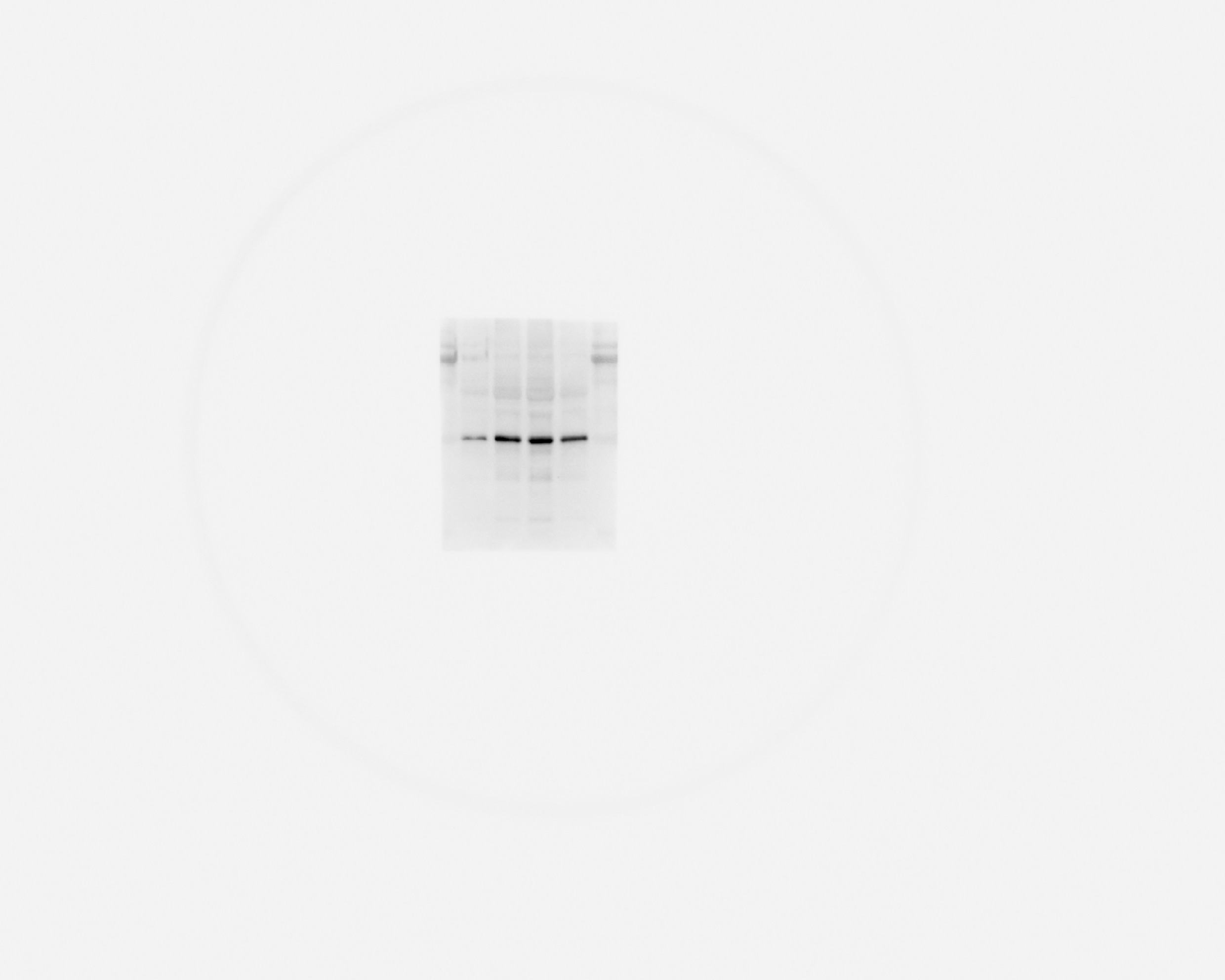

Supplement: Supplementary file 4 — Supplementary Material 4 [file 41598_2026_36354_MOESM4_ESM.zip › Full uncropped Gels and Blots image(s)/Fig.6/Fig.6C/GSDMD-N.tif]

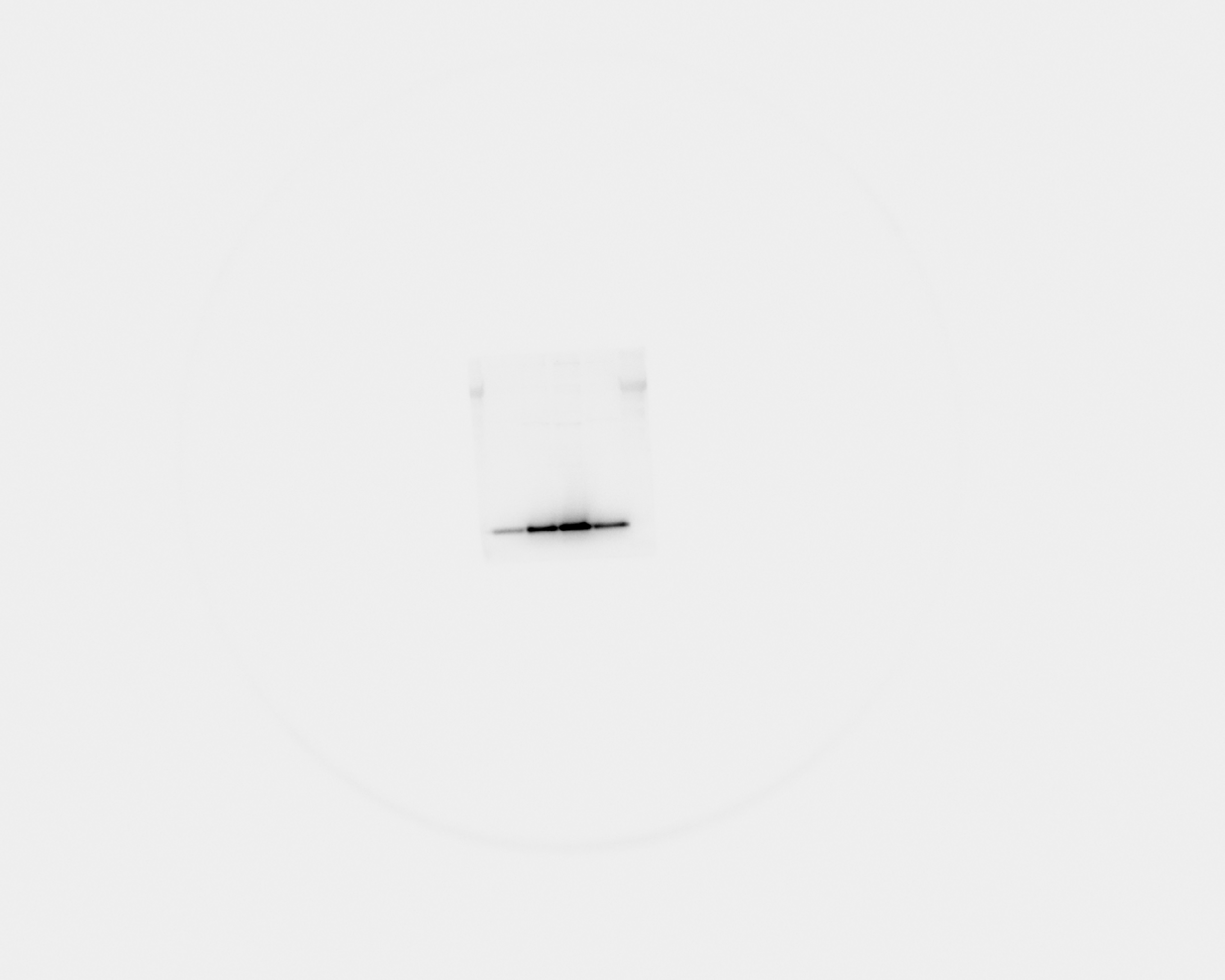

Supplement: Supplementary file 4 — Supplementary Material 4 [file 41598_2026_36354_MOESM4_ESM.zip › Full uncropped Gels and Blots image(s)/Fig.6/Fig.6C/IL-18-1.tif]

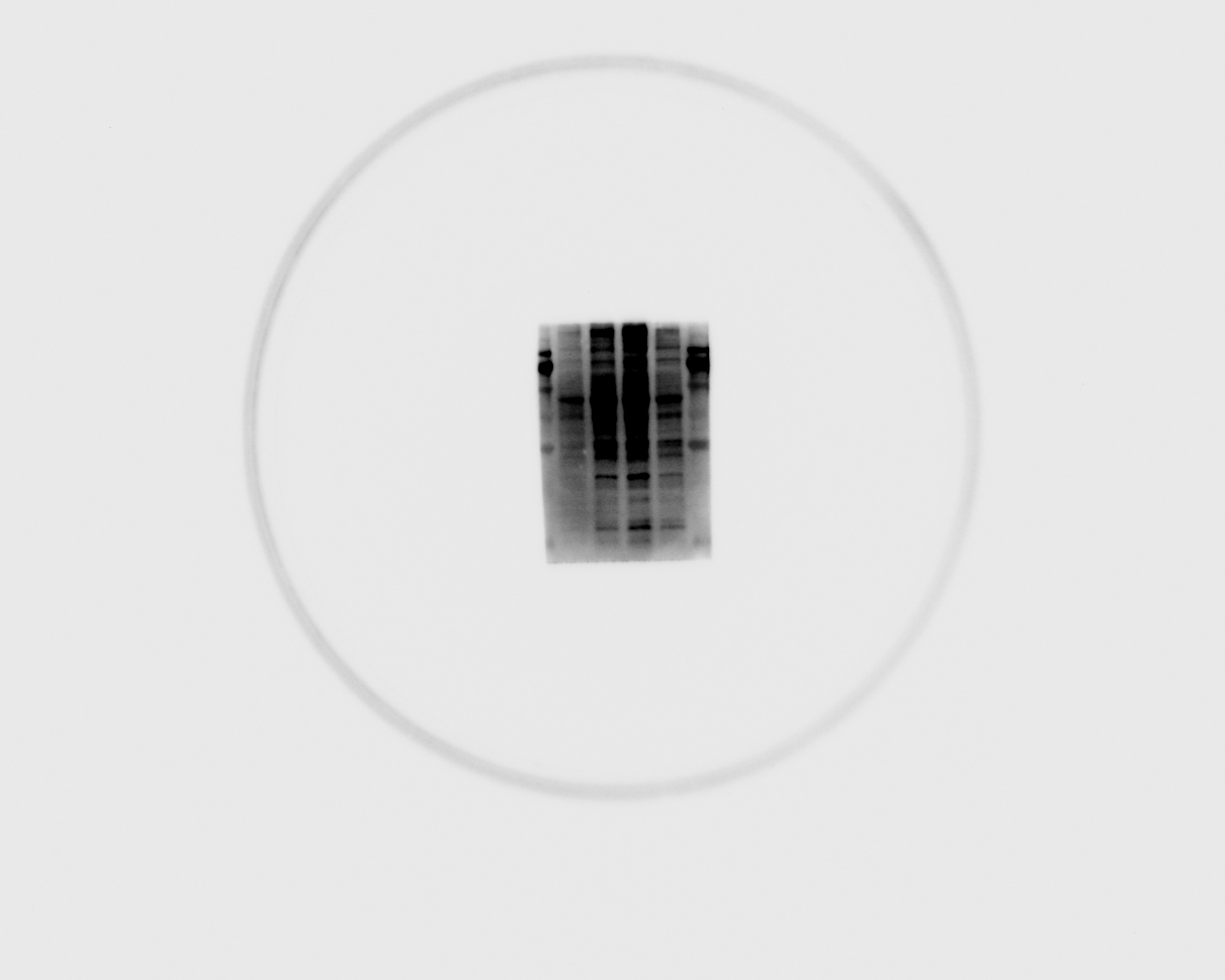

Supplement: Supplementary file 4 — Supplementary Material 4 [file 41598_2026_36354_MOESM4_ESM.zip › Full uncropped Gels and Blots image(s)/Fig.6/Fig.6C/IL1β-1.tif]

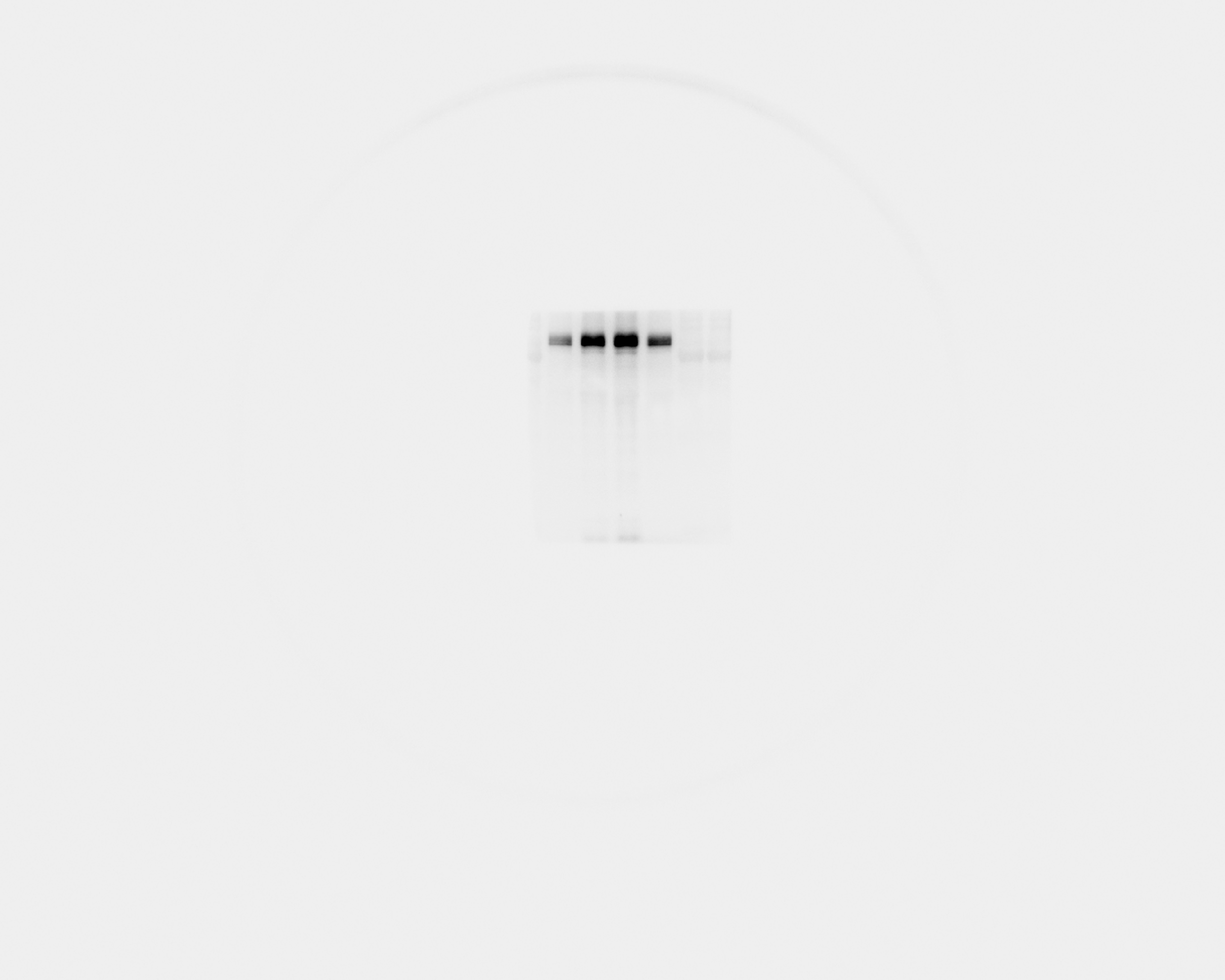

Supplement: Supplementary file 4 — Supplementary Material 4 [file 41598_2026_36354_MOESM4_ESM.zip › Full uncropped Gels and Blots image(s)/Fig.6/Fig.6C/NLRP3-1.tif]

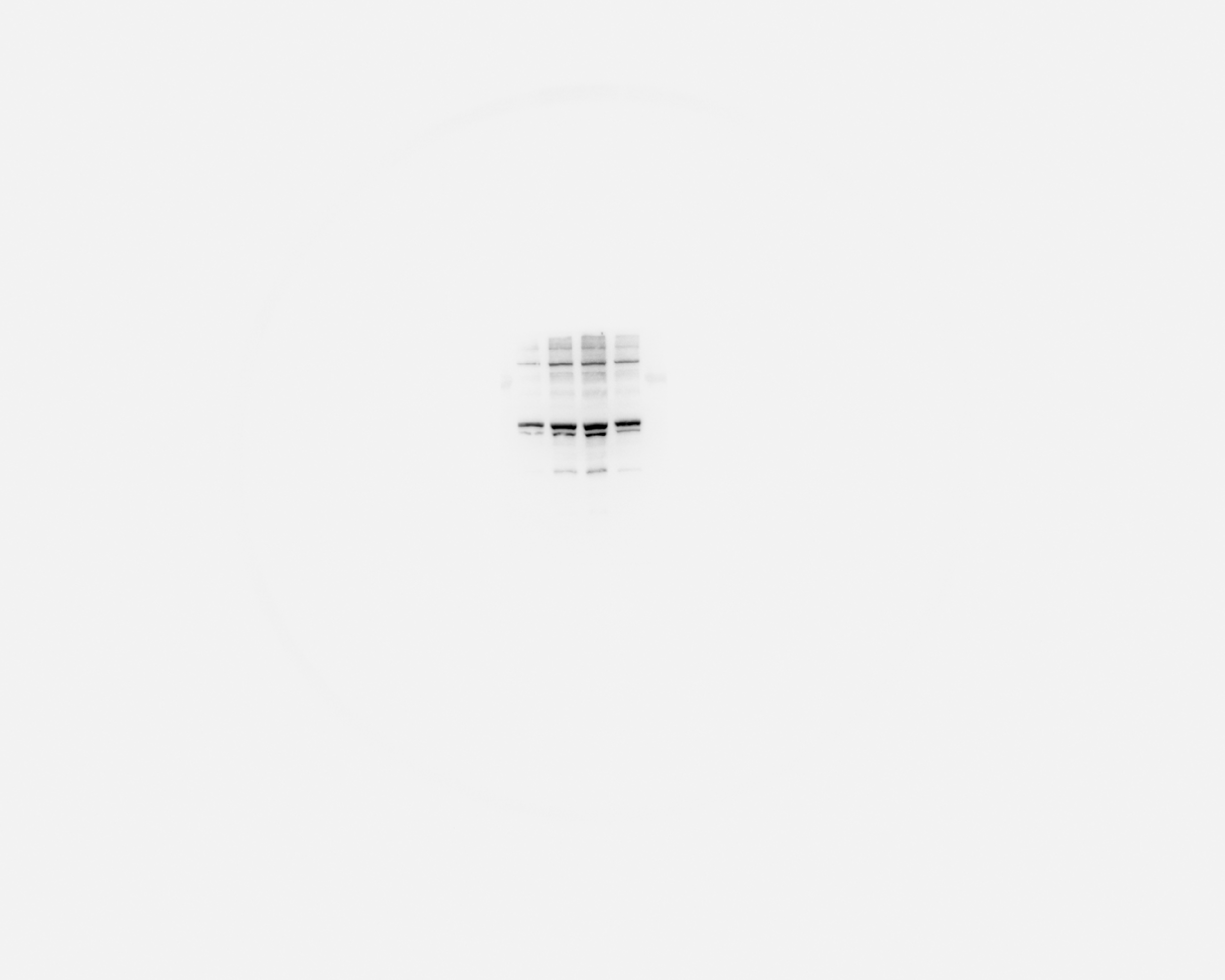

Supplement: Supplementary file 4 — Supplementary Material 4 [file 41598_2026_36354_MOESM4_ESM.zip › Full uncropped Gels and Blots image(s)/Fig.6/Fig.6C/Pro-caspase-1.tif]

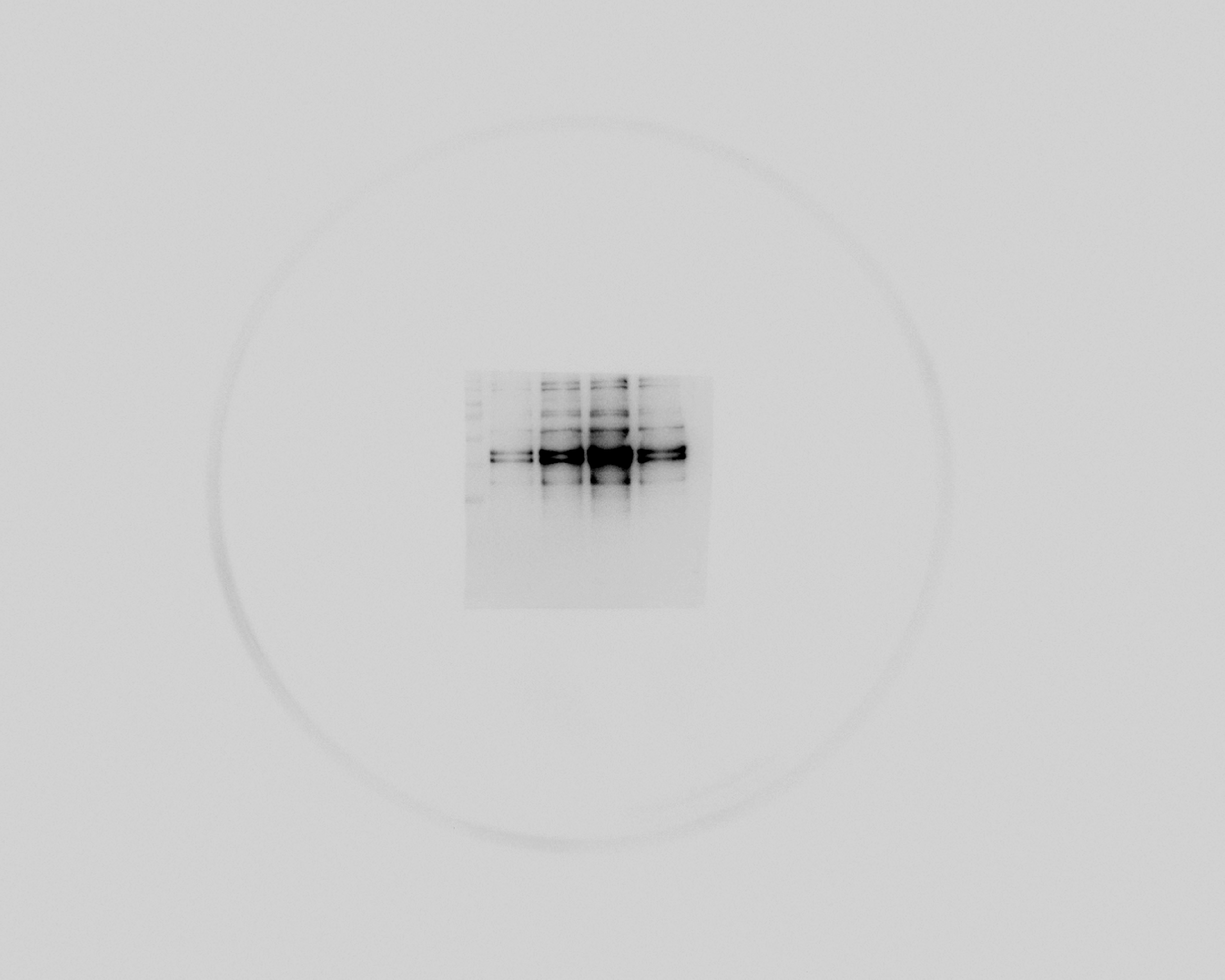

Supplement: Supplementary file 4 — Supplementary Material 4 [file 41598_2026_36354_MOESM4_ESM.zip › Full uncropped Gels and Blots image(s)/Fig.6/Fig.6C/TXNIP.tif]

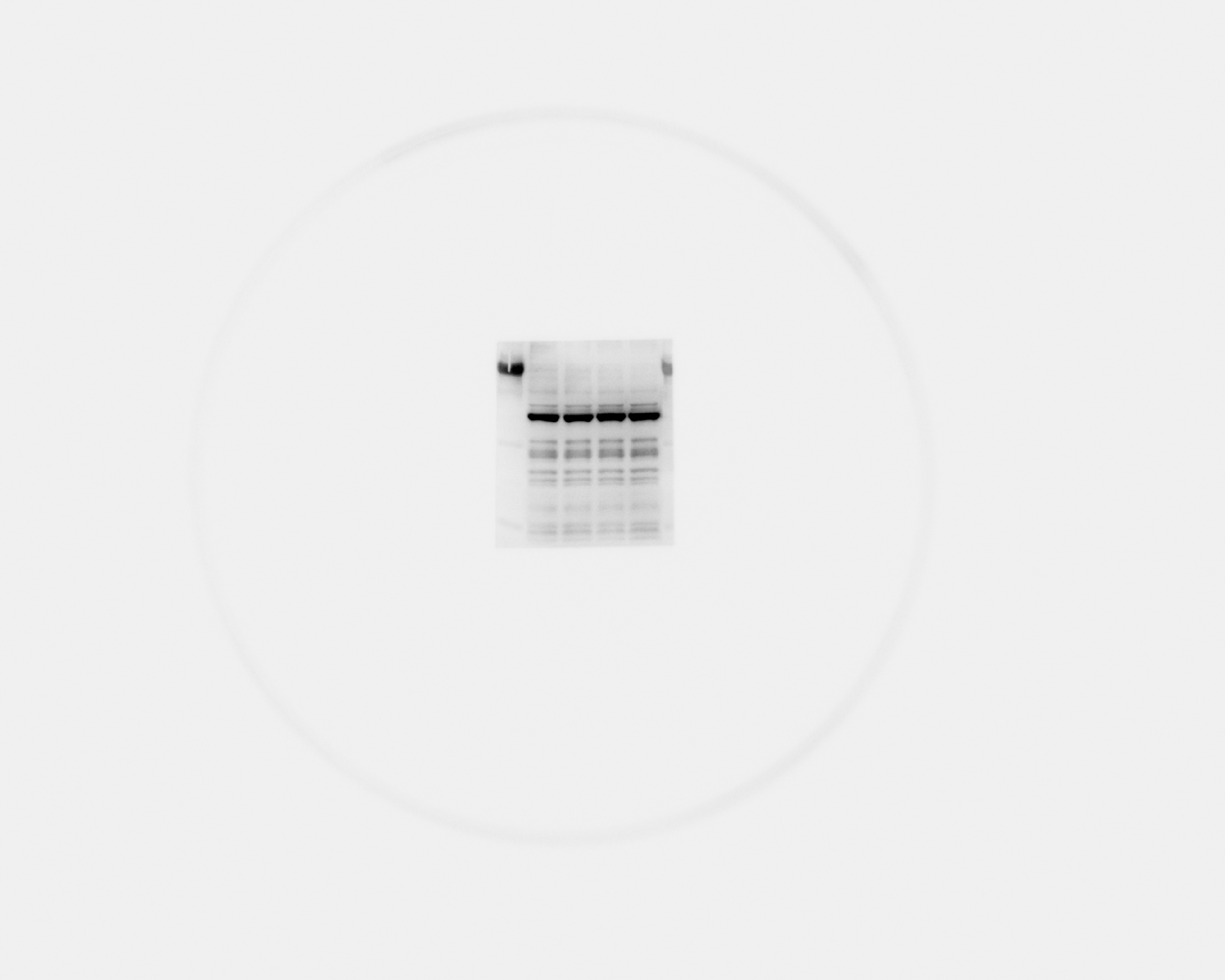

Supplement: Supplementary file 4 — Supplementary Material 4 [file 41598_2026_36354_MOESM4_ESM.zip › Full uncropped Gels and Blots image(s)/Fig.6/Fig.6C/β-actin.tif]

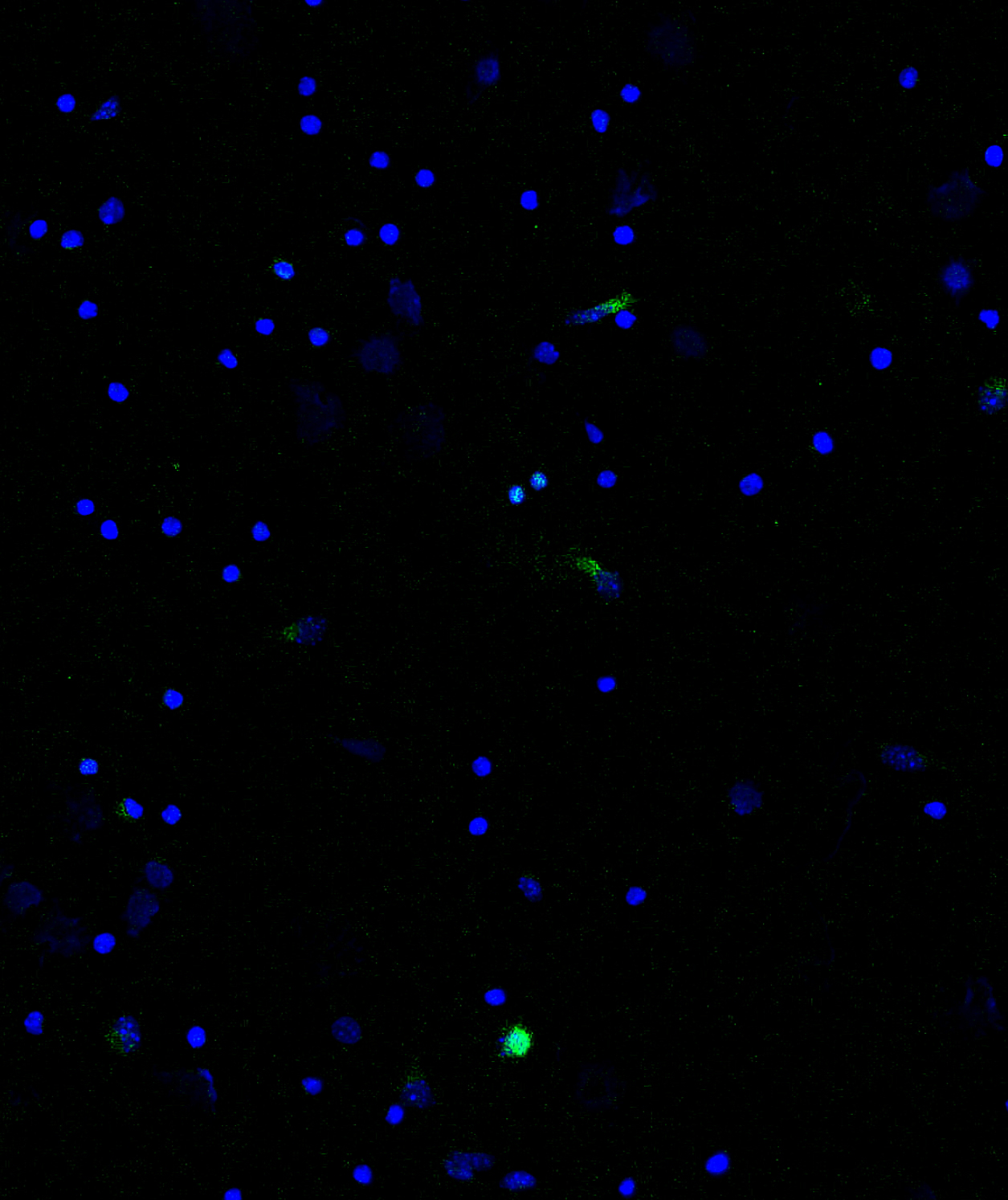

Supplement: Supplementary file 4 — Supplementary Material 4 [file 41598_2026_36354_MOESM4_ESM.zip › Full uncropped Gels and Blots image(s)/Fig.7/Fig.7A/NS+KD.jpg]

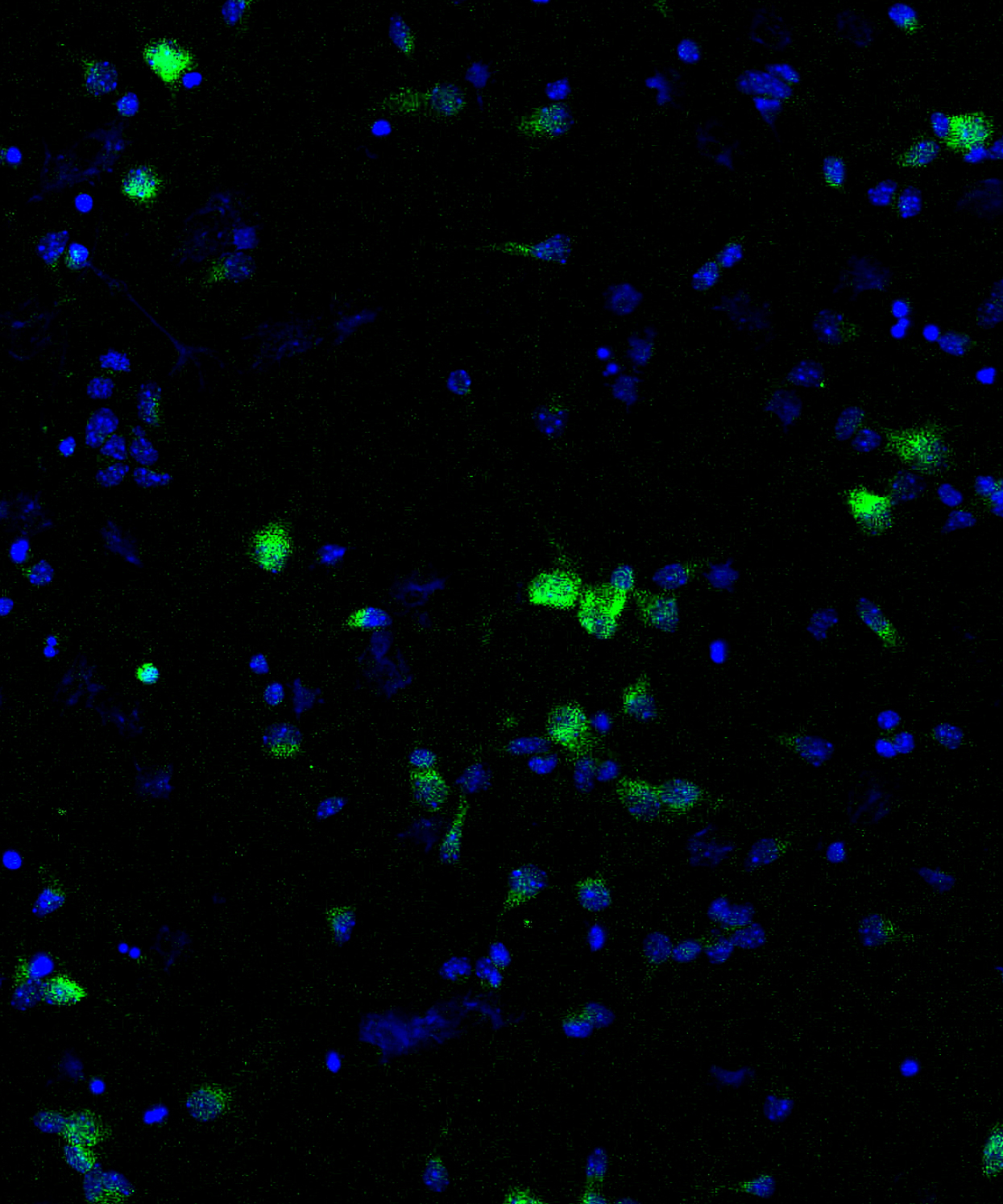

Supplement: Supplementary file 4 — Supplementary Material 4 [file 41598_2026_36354_MOESM4_ESM.zip › Full uncropped Gels and Blots image(s)/Fig.7/Fig.7A/NS+OE.jpg]

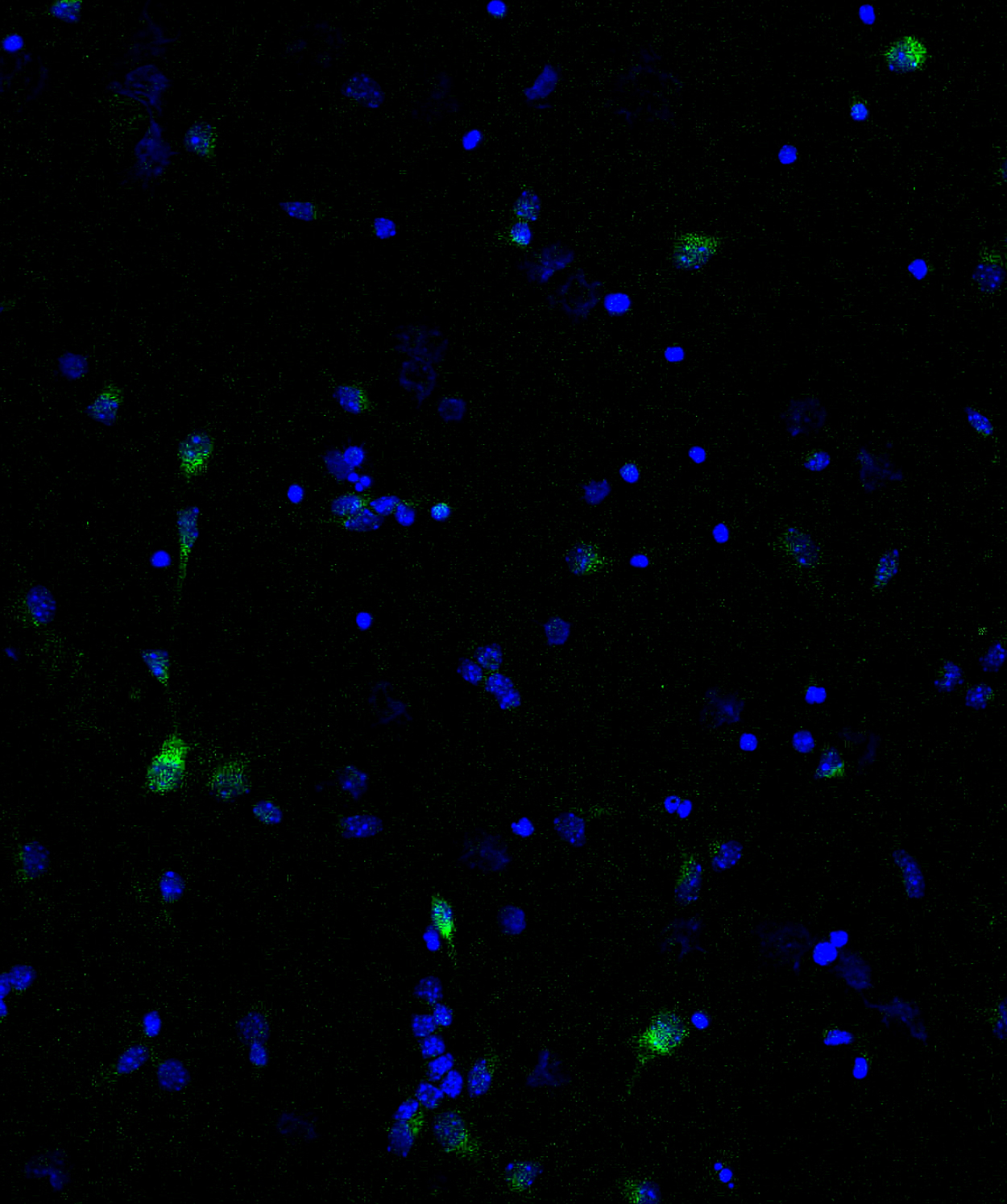

Supplement: Supplementary file 4 — Supplementary Material 4 [file 41598_2026_36354_MOESM4_ESM.zip › Full uncropped Gels and Blots image(s)/Fig.7/Fig.7A/NS.jpg]

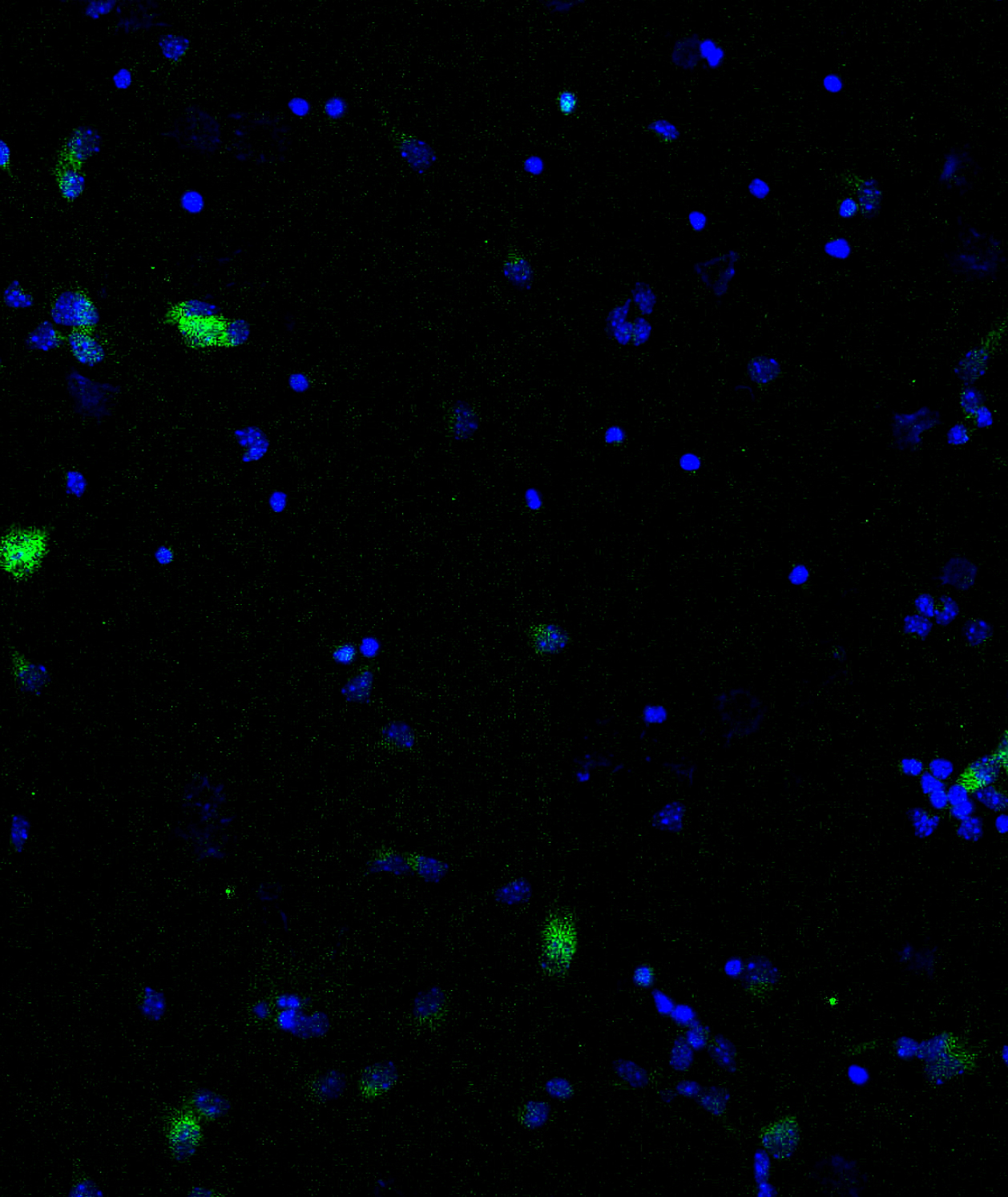

Supplement: Supplementary file 4 — Supplementary Material 4 [file 41598_2026_36354_MOESM4_ESM.zip › Full uncropped Gels and Blots image(s)/Fig.7/Fig.7A/TMAO+KD.jpg]

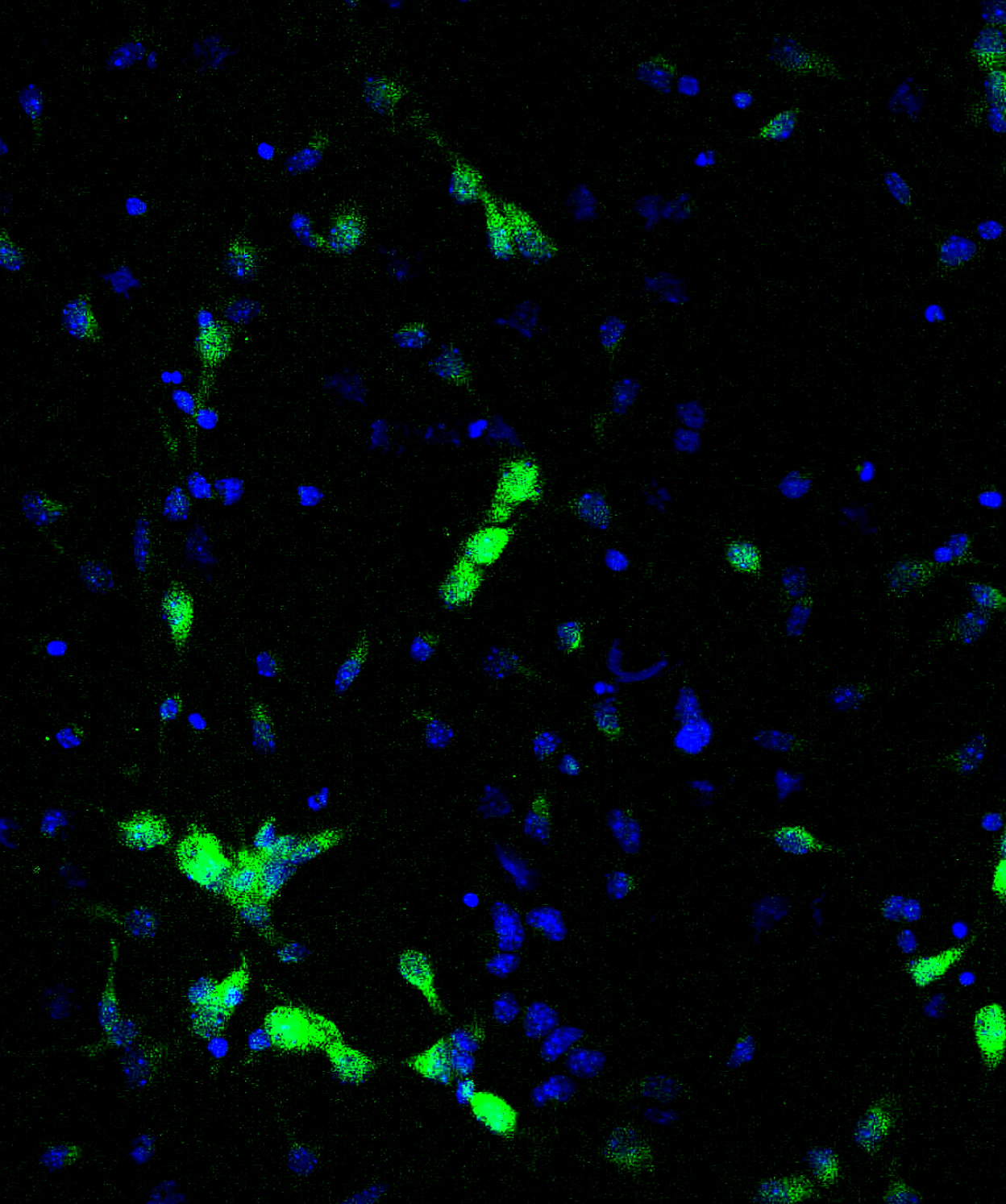

Supplement: Supplementary file 4 — Supplementary Material 4 [file 41598_2026_36354_MOESM4_ESM.zip › Full uncropped Gels and Blots image(s)/Fig.7/Fig.7A/TMAO+OE.jpg]

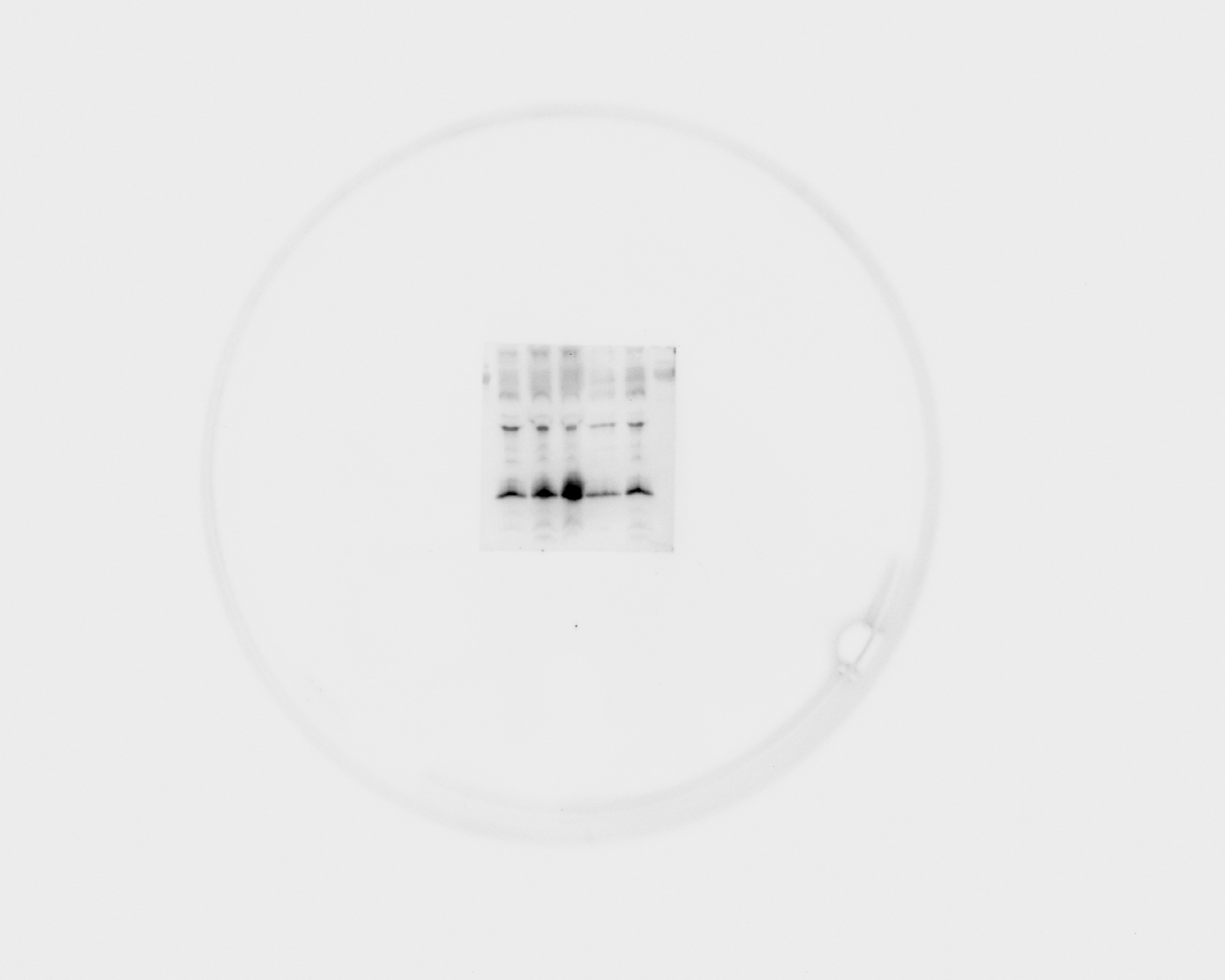

Supplement: Supplementary file 4 — Supplementary Material 4 [file 41598_2026_36354_MOESM4_ESM.zip › Full uncropped Gels and Blots image(s)/Fig.7/Fig.7C/ASC.tif]

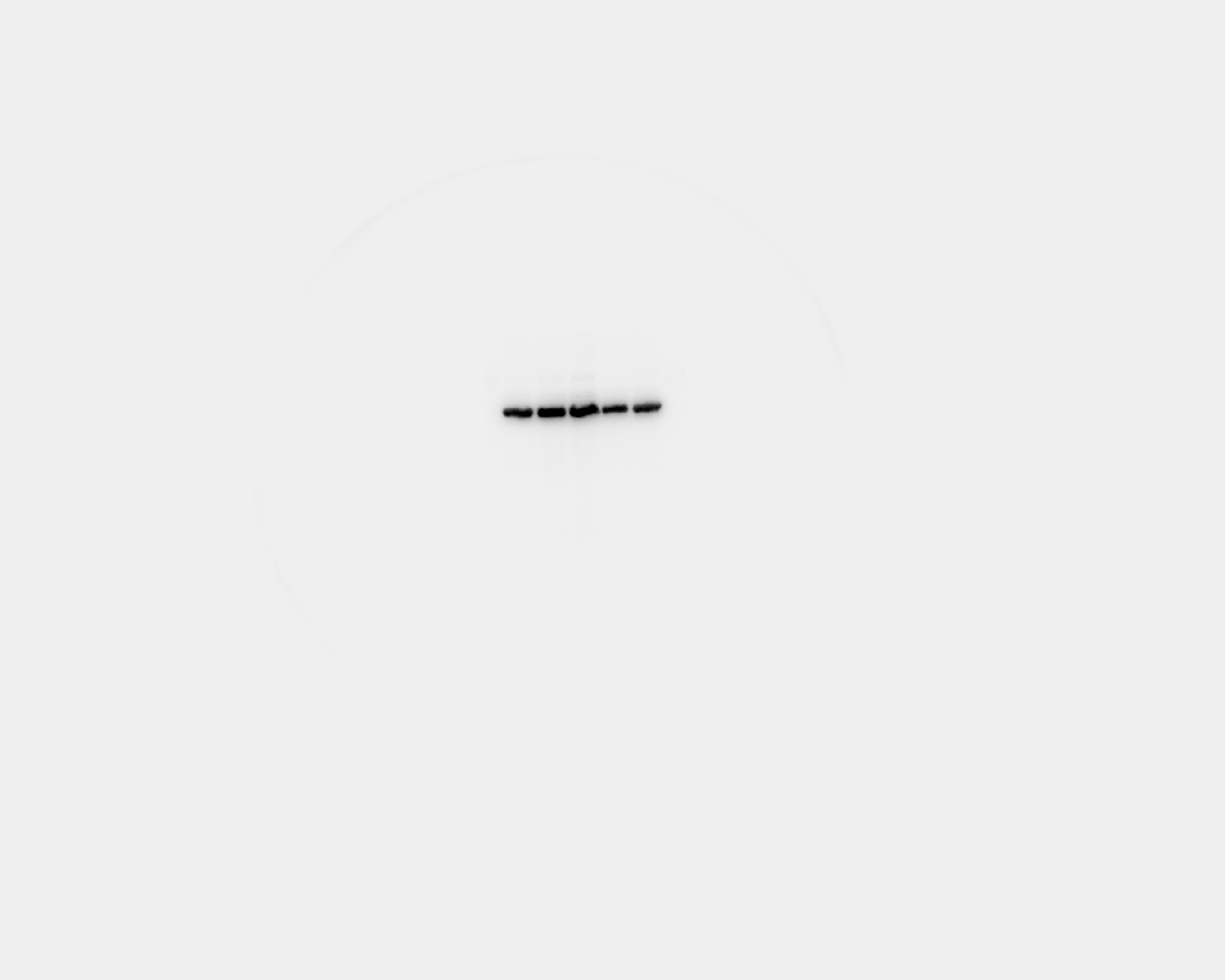

Supplement: Supplementary file 4 — Supplementary Material 4 [file 41598_2026_36354_MOESM4_ESM.zip › Full uncropped Gels and Blots image(s)/Fig.7/Fig.7C/Caspase-1.tif]

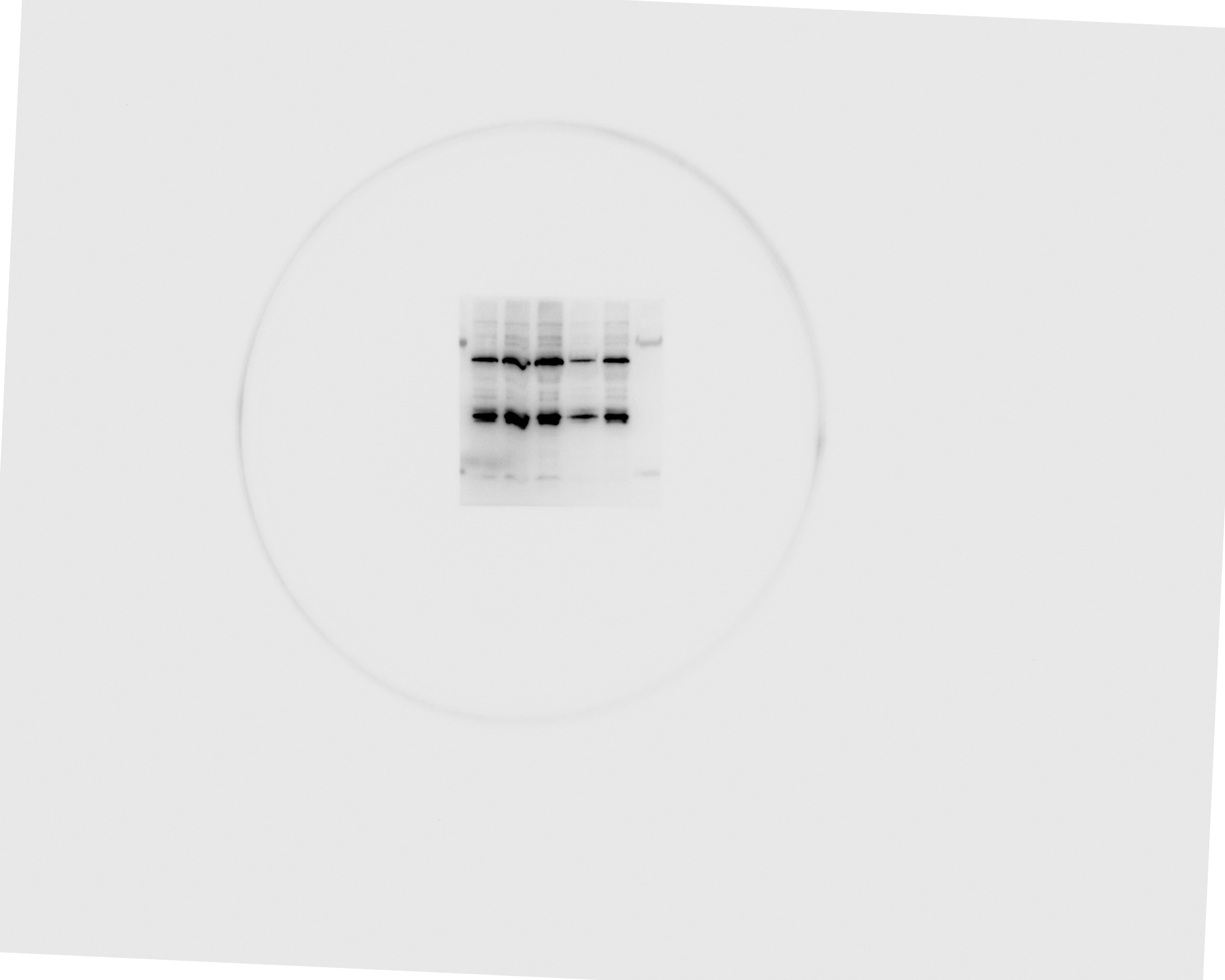

Supplement: Supplementary file 4 — Supplementary Material 4 [file 41598_2026_36354_MOESM4_ESM.zip › Full uncropped Gels and Blots image(s)/Fig.7/Fig.7C/GSDMD-FL.tif]

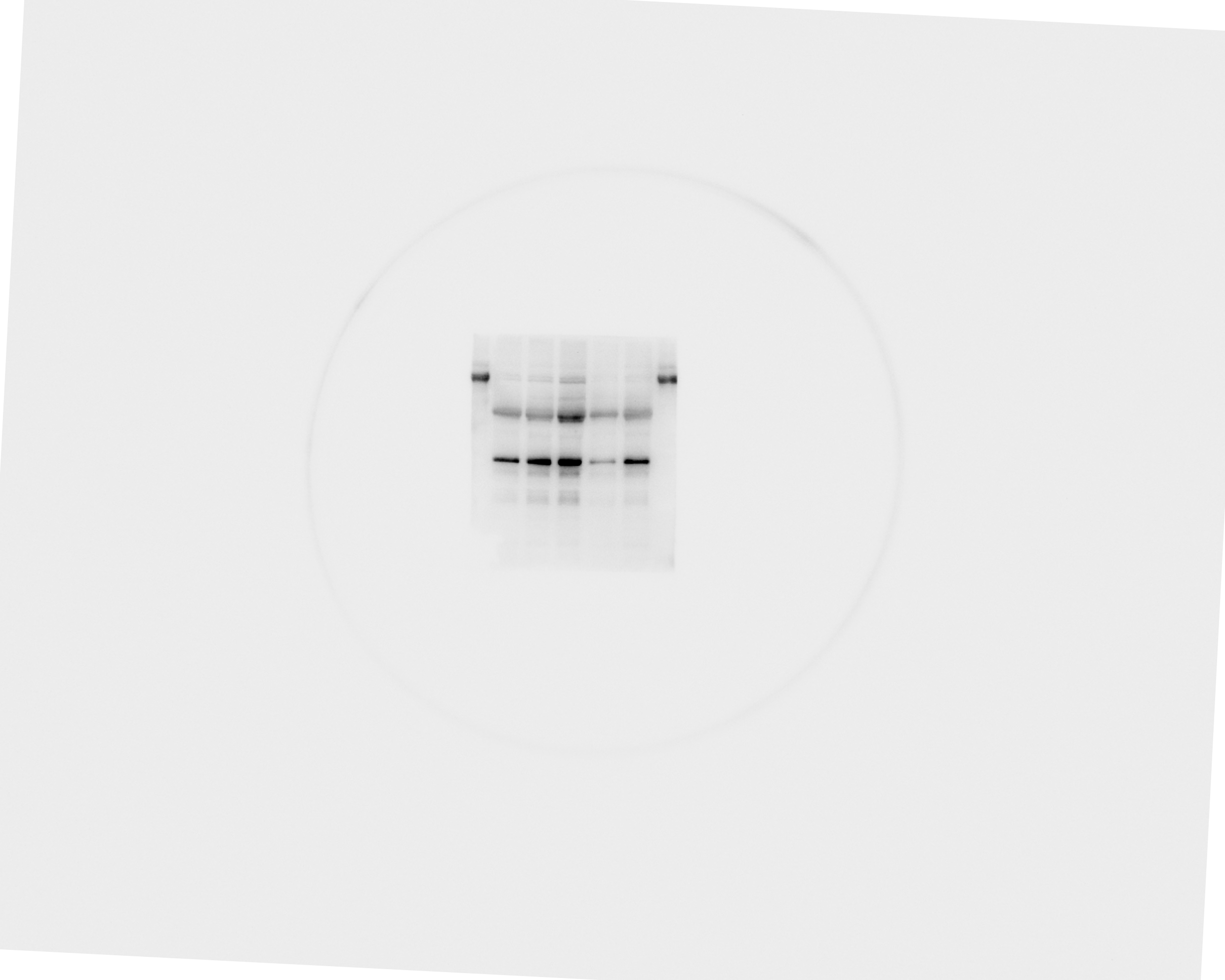

Supplement: Supplementary file 4 — Supplementary Material 4 [file 41598_2026_36354_MOESM4_ESM.zip › Full uncropped Gels and Blots image(s)/Fig.7/Fig.7C/GSDMD-N.tif]

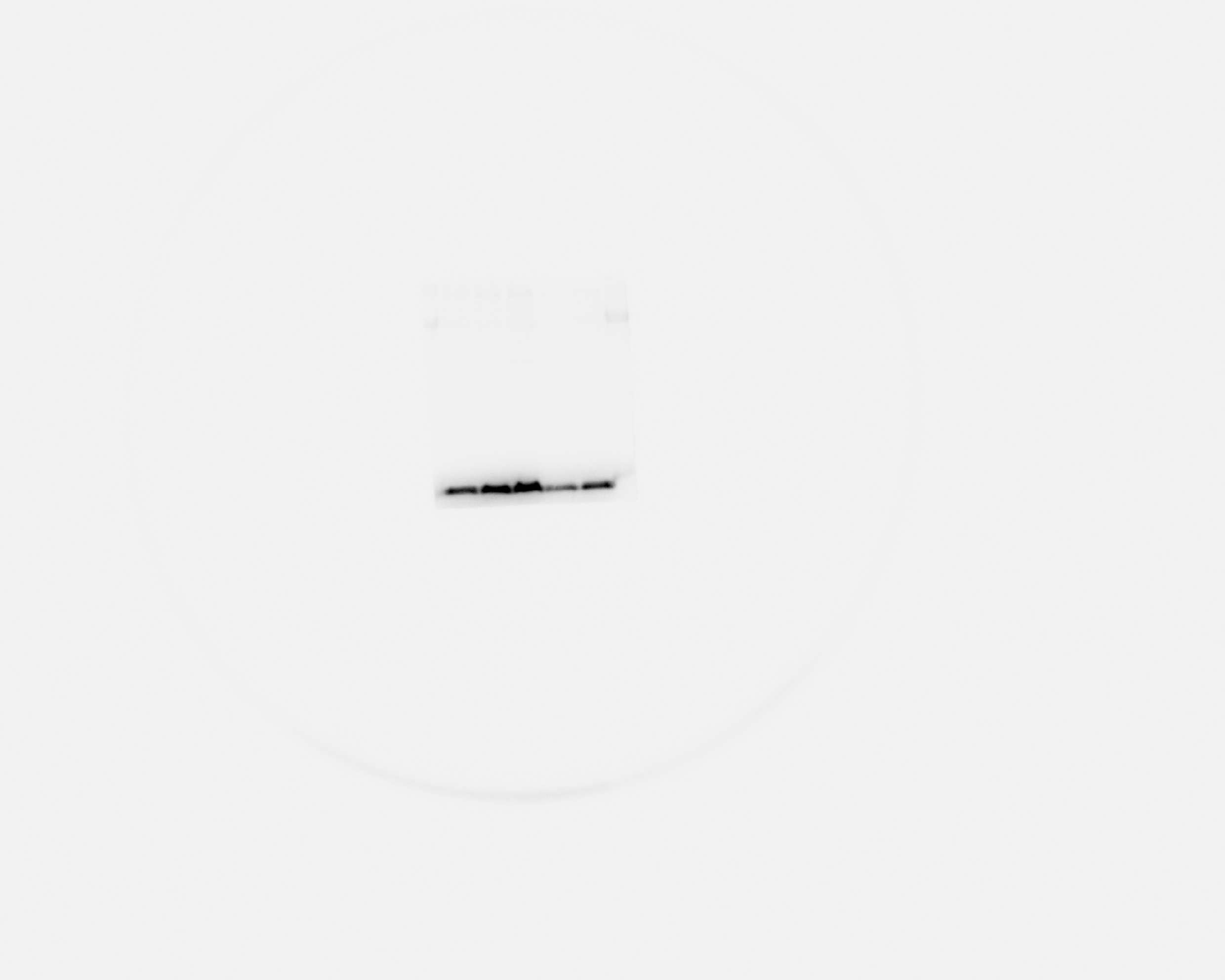

Supplement: Supplementary file 4 — Supplementary Material 4 [file 41598_2026_36354_MOESM4_ESM.zip › Full uncropped Gels and Blots image(s)/Fig.7/Fig.7C/IL-18.tif]

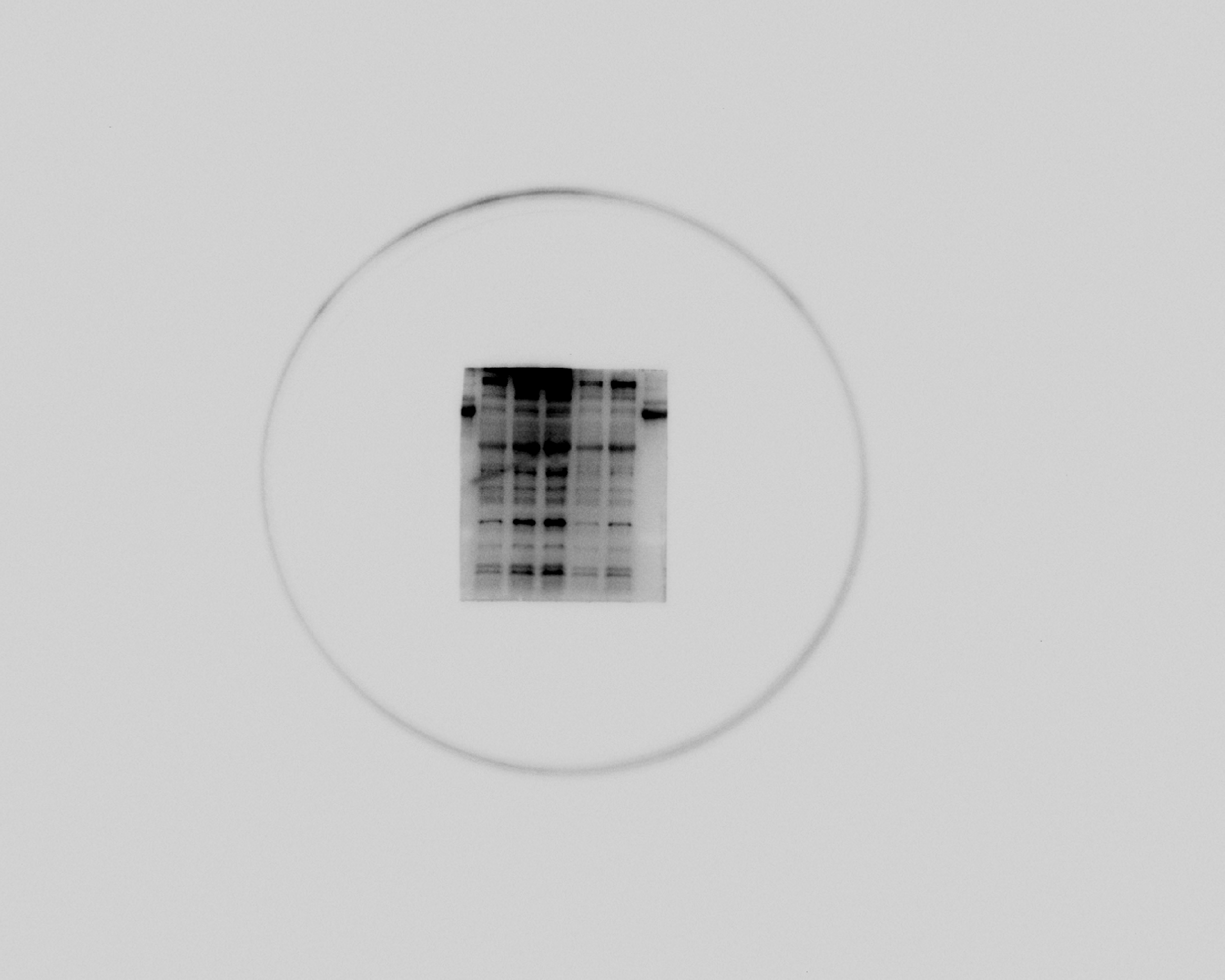

Supplement: Supplementary file 4 — Supplementary Material 4 [file 41598_2026_36354_MOESM4_ESM.zip › Full uncropped Gels and Blots image(s)/Fig.7/Fig.7C/IL-1β.tif]

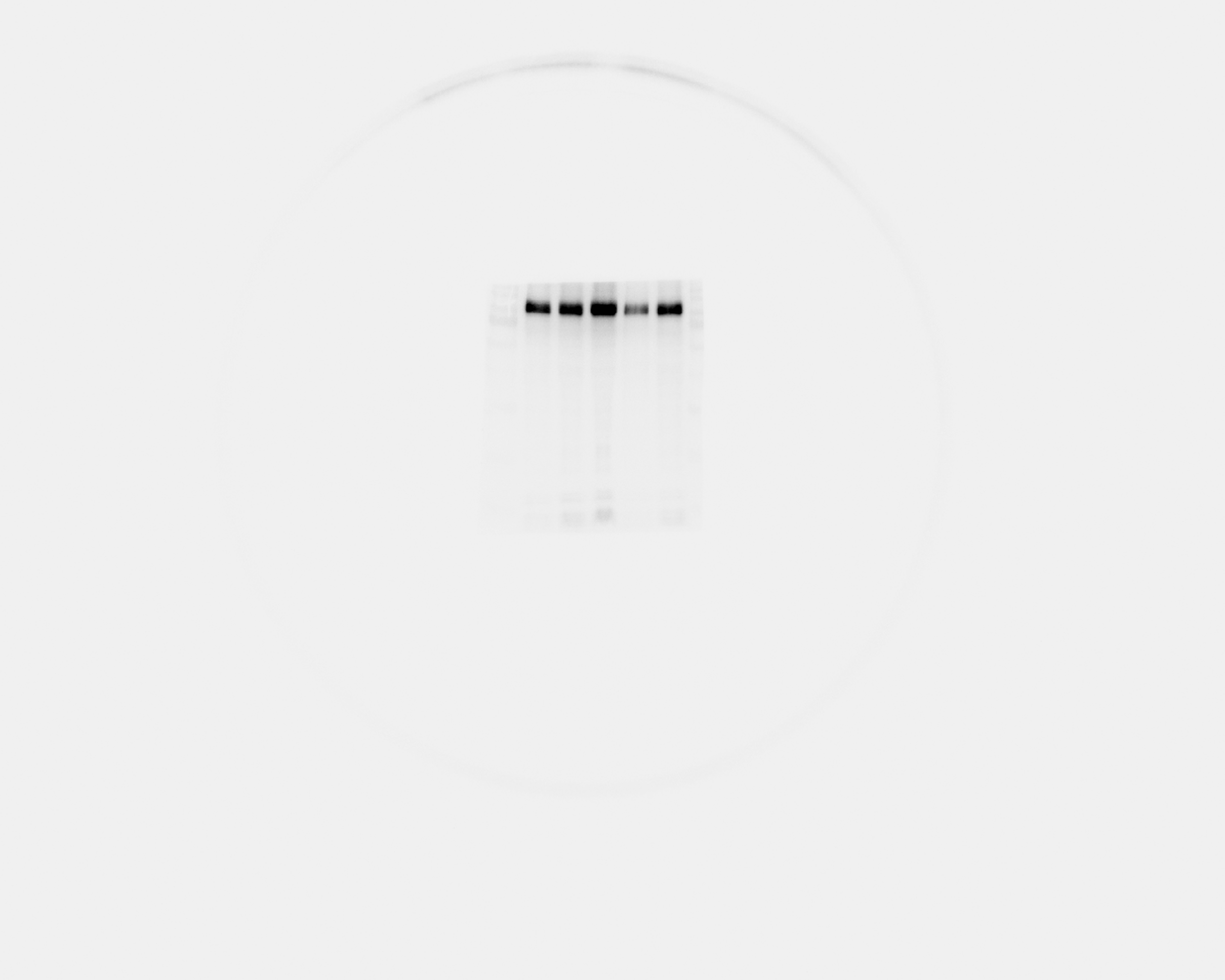

Supplement: Supplementary file 4 — Supplementary Material 4 [file 41598_2026_36354_MOESM4_ESM.zip › Full uncropped Gels and Blots image(s)/Fig.7/Fig.7C/NLRP3.tif]

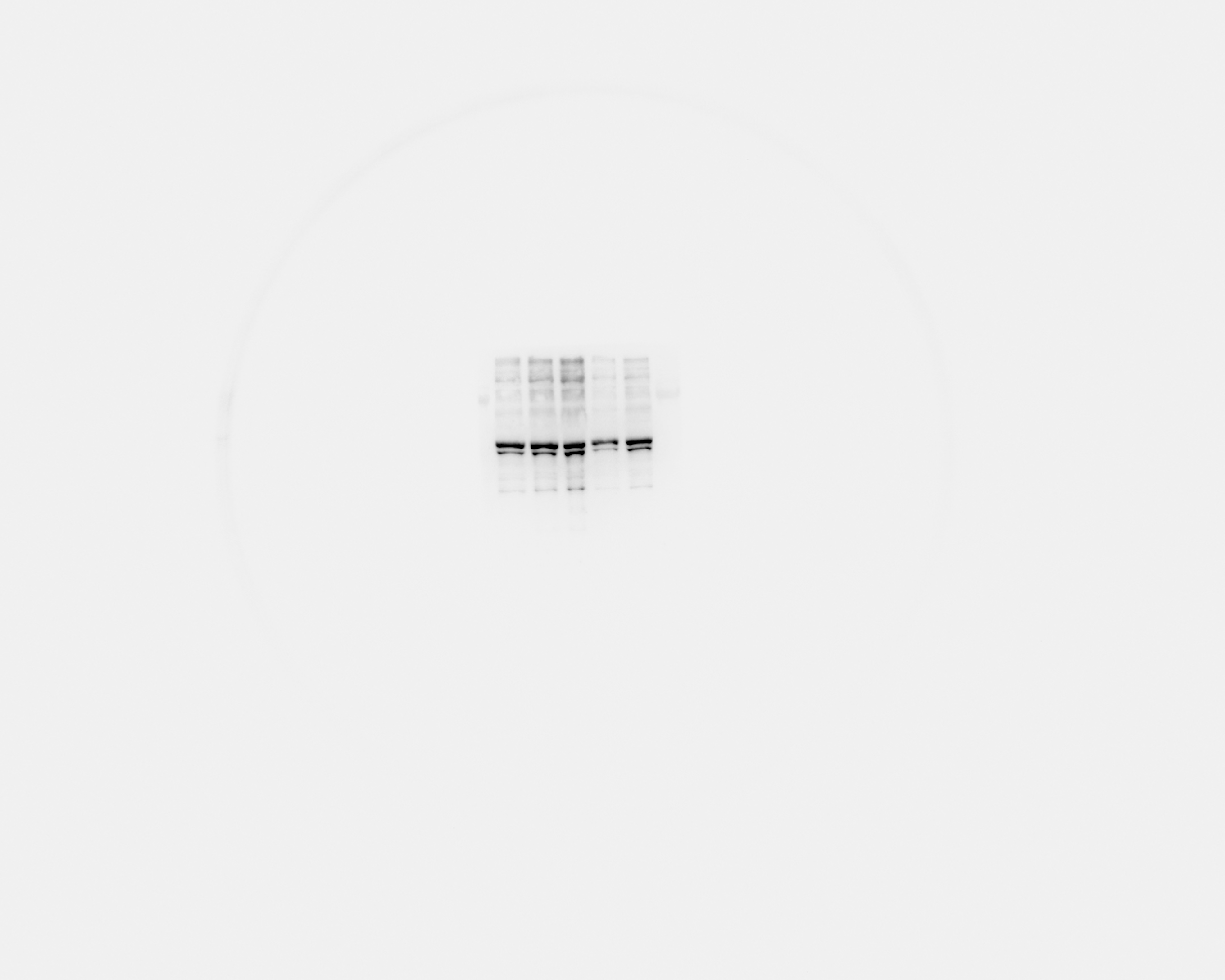

Supplement: Supplementary file 4 — Supplementary Material 4 [file 41598_2026_36354_MOESM4_ESM.zip › Full uncropped Gels and Blots image(s)/Fig.7/Fig.7C/Pro-caspase-1.tif]

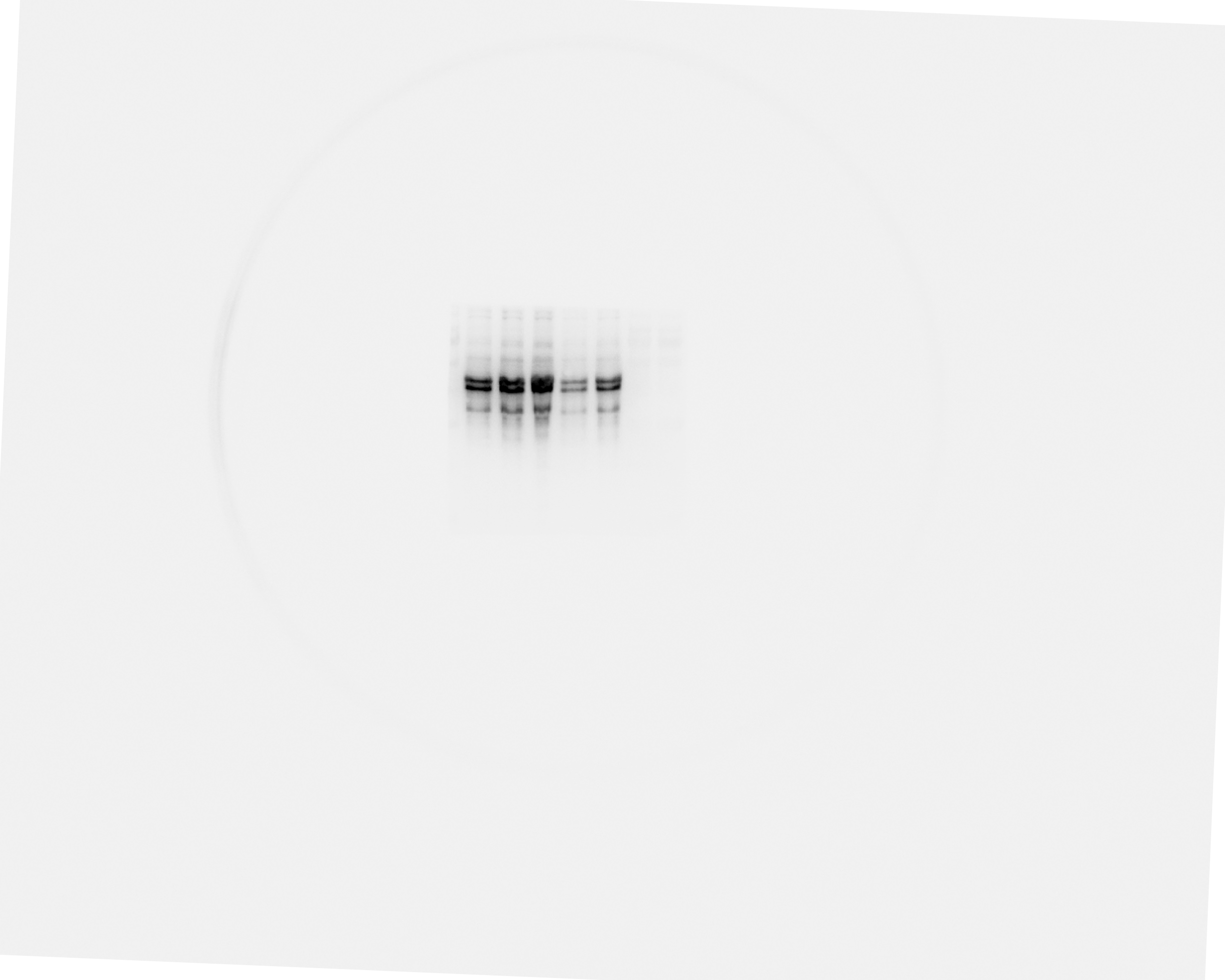

Supplement: Supplementary file 4 — Supplementary Material 4 [file 41598_2026_36354_MOESM4_ESM.zip › Full uncropped Gels and Blots image(s)/Fig.7/Fig.7C/TXNIP-2.tif]

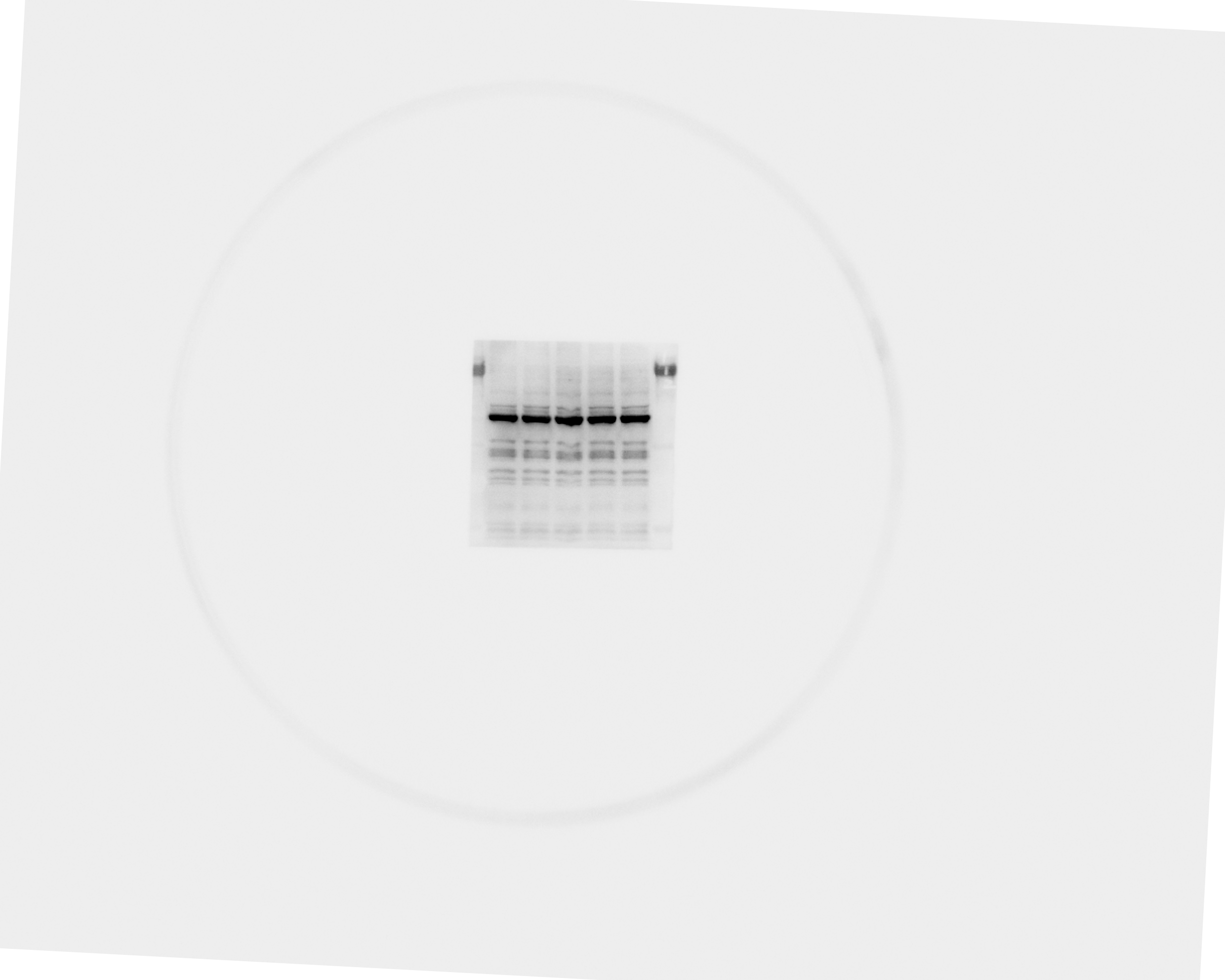

Supplement: Supplementary file 4 — Supplementary Material 4 [file 41598_2026_36354_MOESM4_ESM.zip › Full uncropped Gels and Blots image(s)/Fig.7/Fig.7C/β-actin.tif]

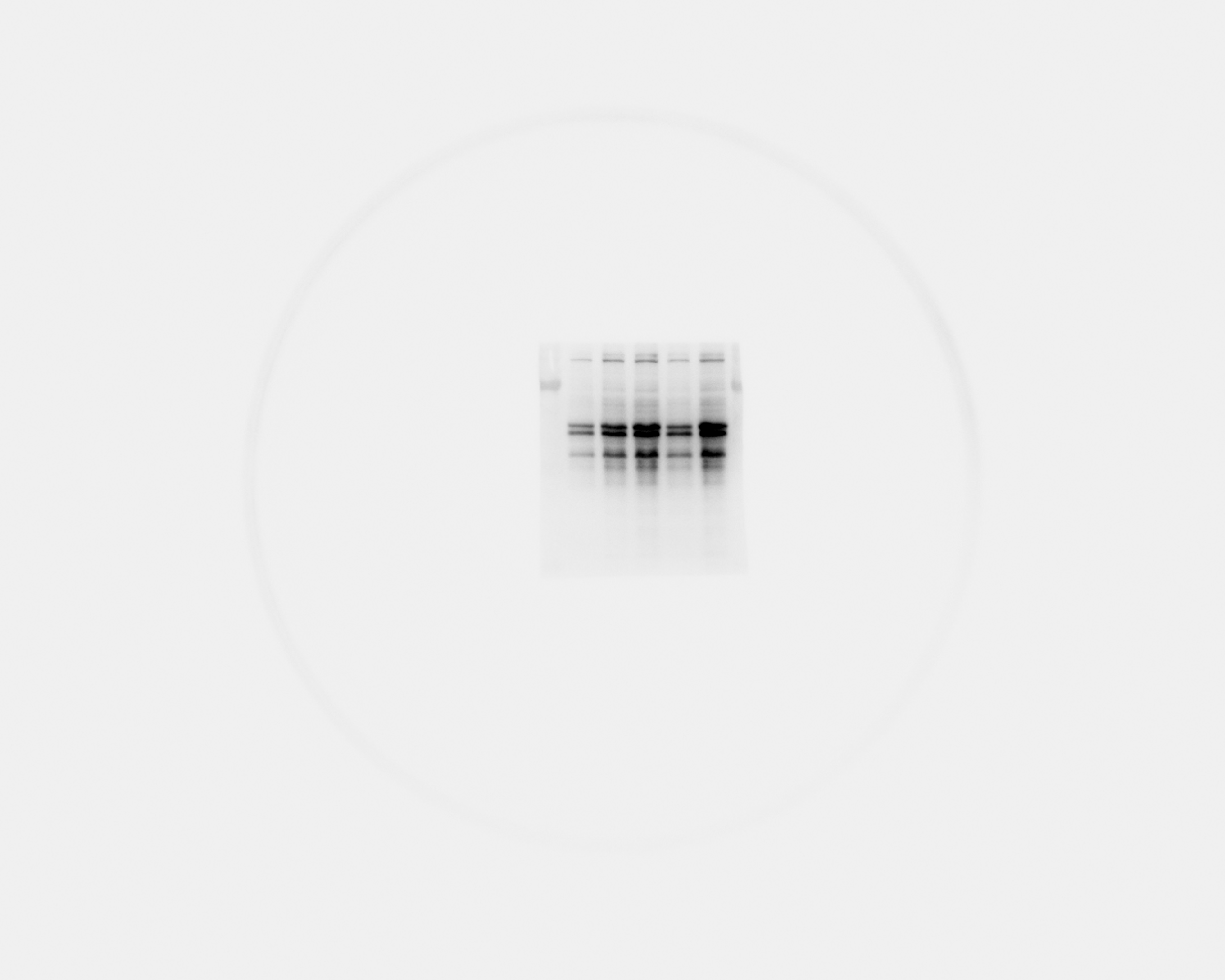

Supplement: Supplementary file 4 — Supplementary Material 4 [file 41598_2026_36354_MOESM4_ESM.zip › Full uncropped Gels and Blots image(s)/Fig.8/Fig.8A TXNIP-1.tif]

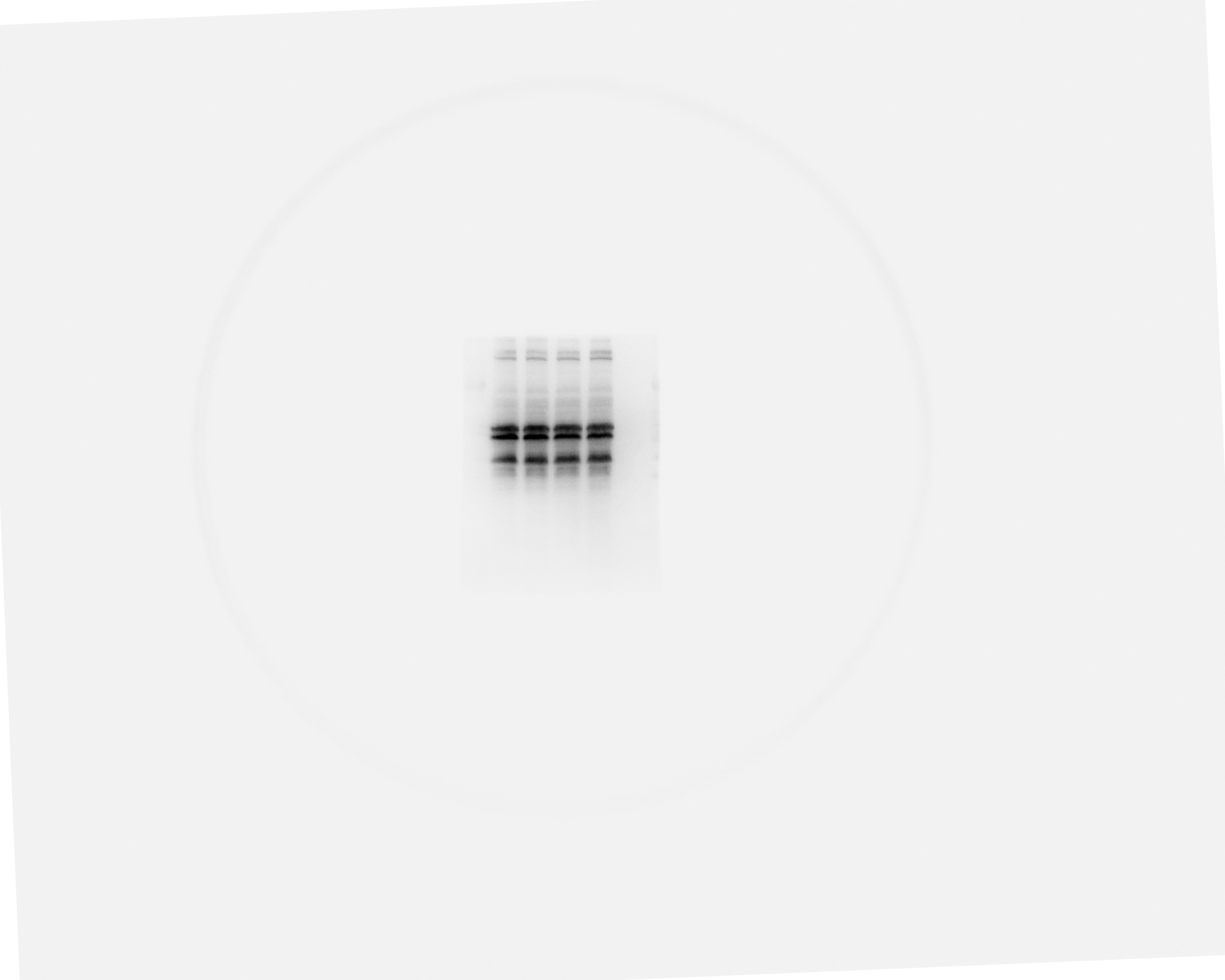

Supplement: Supplementary file 4 — Supplementary Material 4 [file 41598_2026_36354_MOESM4_ESM.zip › Full uncropped Gels and Blots image(s)/Fig.8/Fig.8A TXNIP-2.tif]

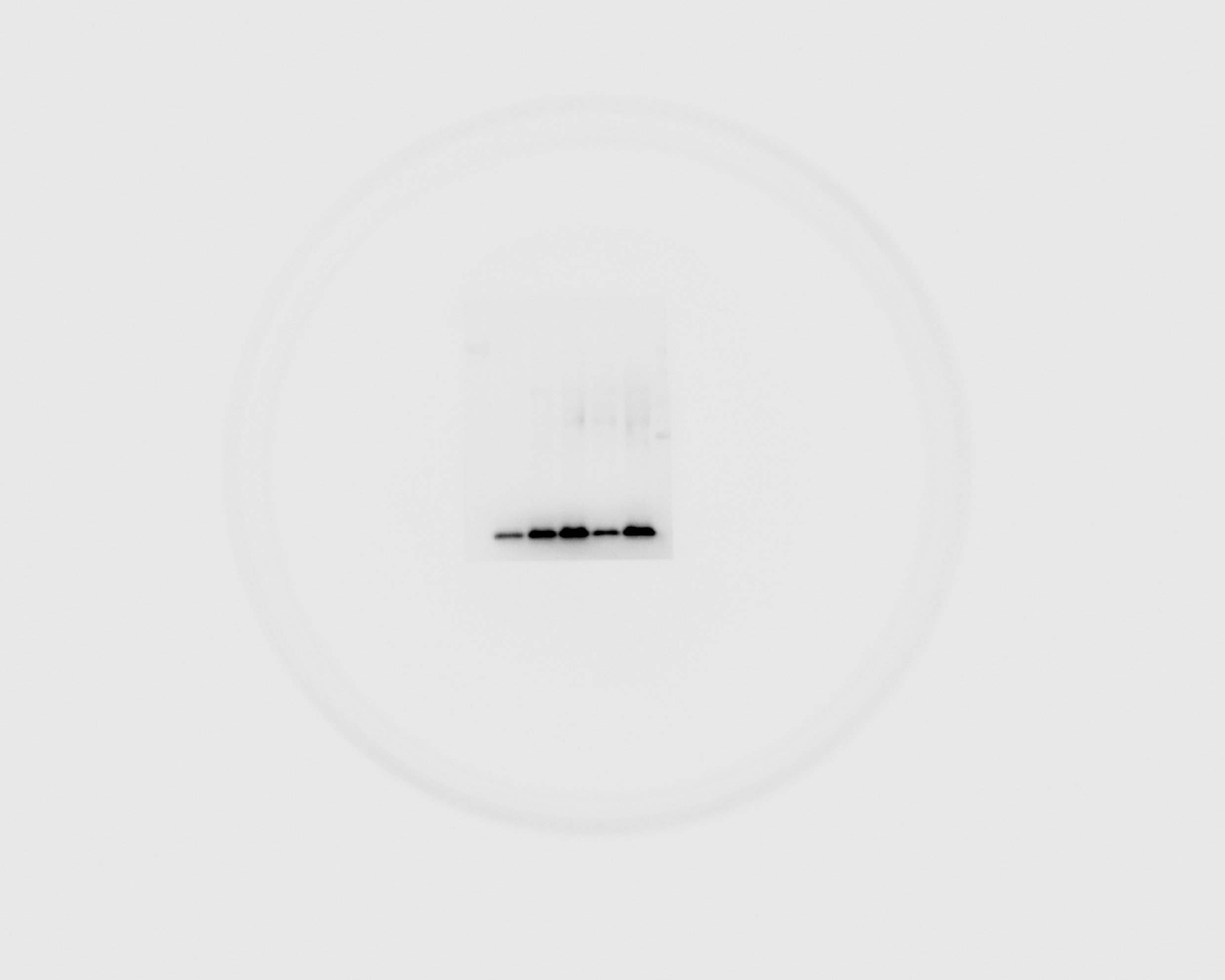

Supplement: Supplementary file 4 — Supplementary Material 4 [file 41598_2026_36354_MOESM4_ESM.zip › Full uncropped Gels and Blots image(s)/Fig.8/Fig.8A Trx1-1 (1).tif]

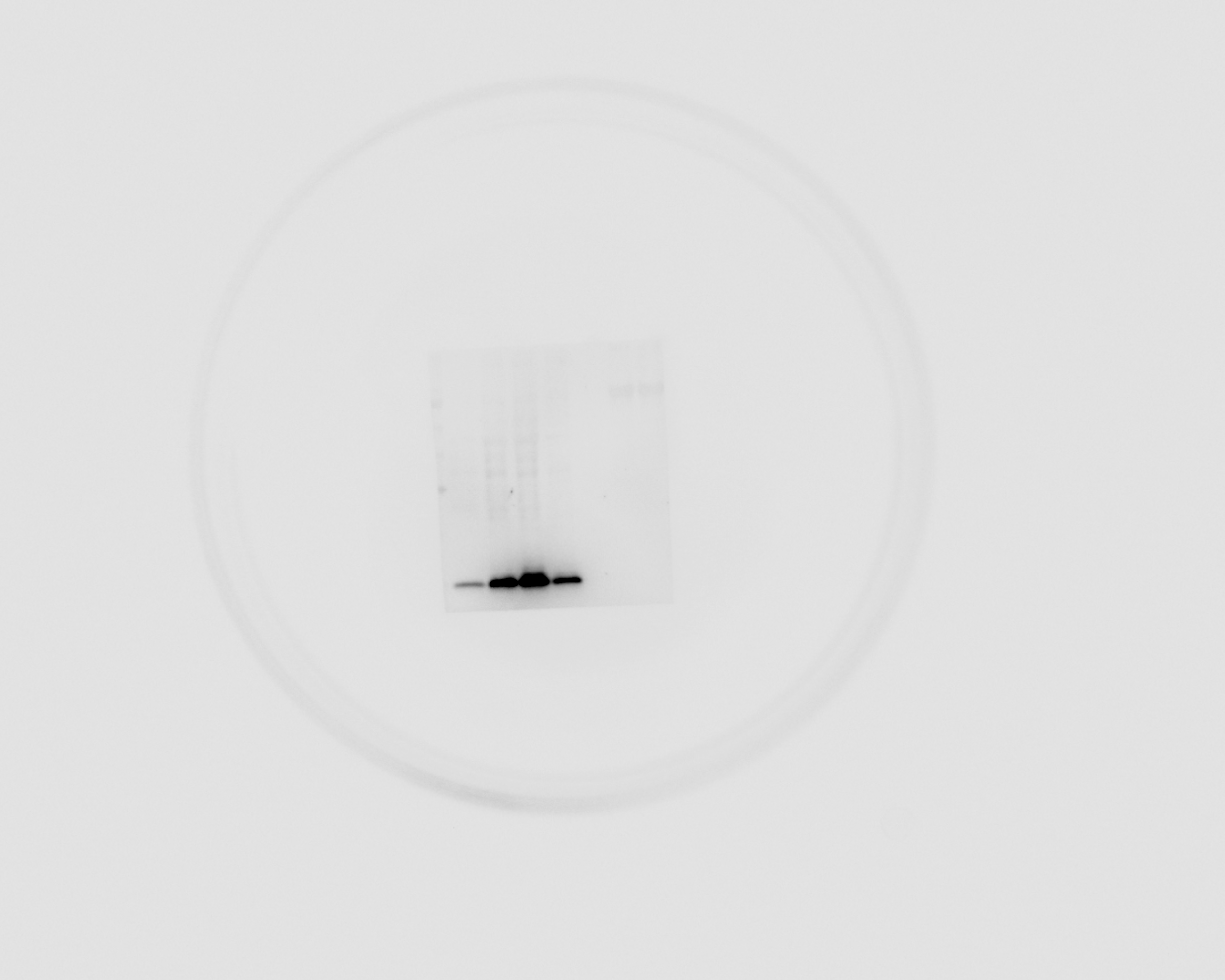

Supplement: Supplementary file 4 — Supplementary Material 4 [file 41598_2026_36354_MOESM4_ESM.zip › Full uncropped Gels and Blots image(s)/Fig.8/Fig.8A Trx1-2.tif]

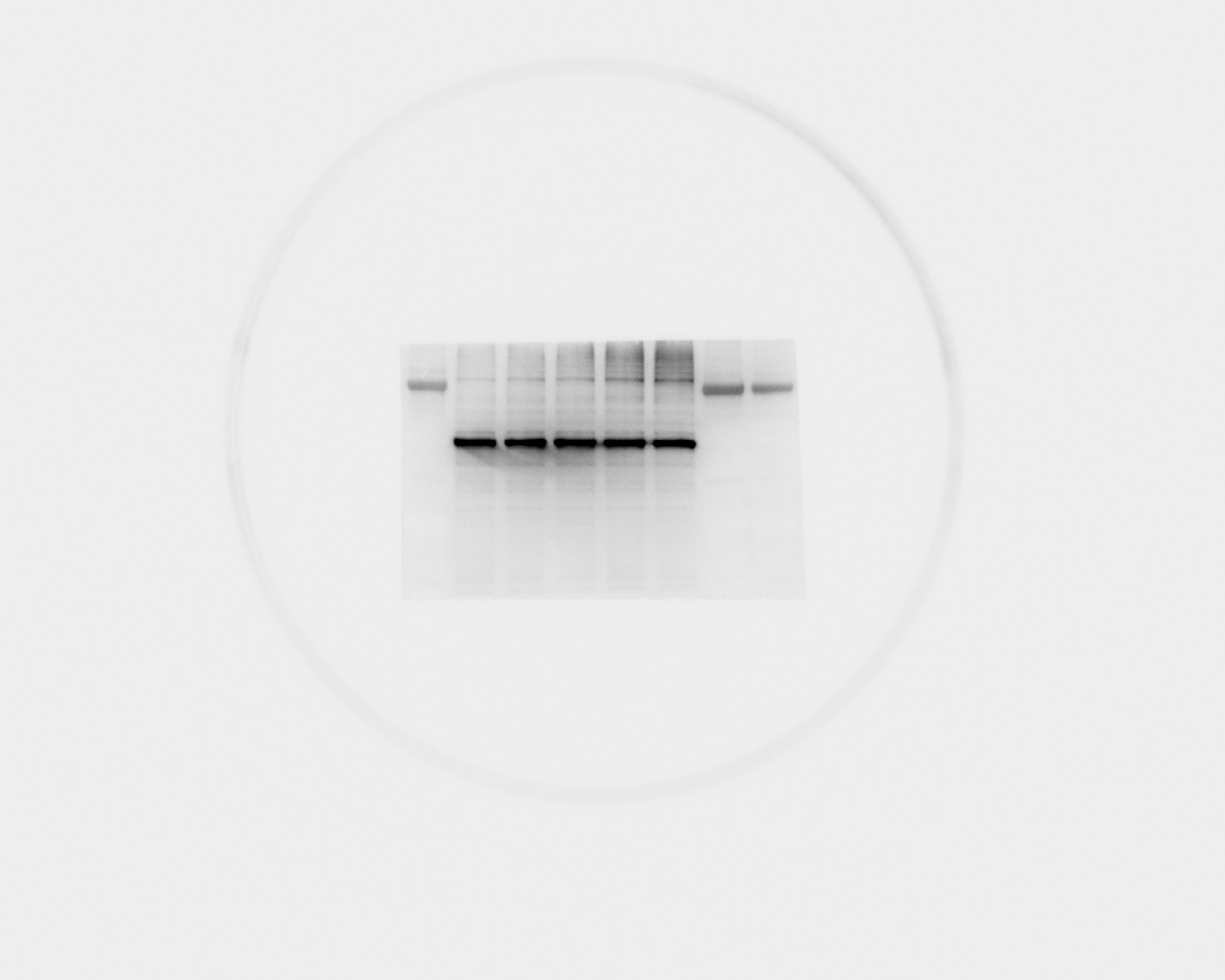

Supplement: Supplementary file 4 — Supplementary Material 4 [file 41598_2026_36354_MOESM4_ESM.zip › Full uncropped Gels and Blots image(s)/Fig.8/Fig.8A β-actin-1.tif]

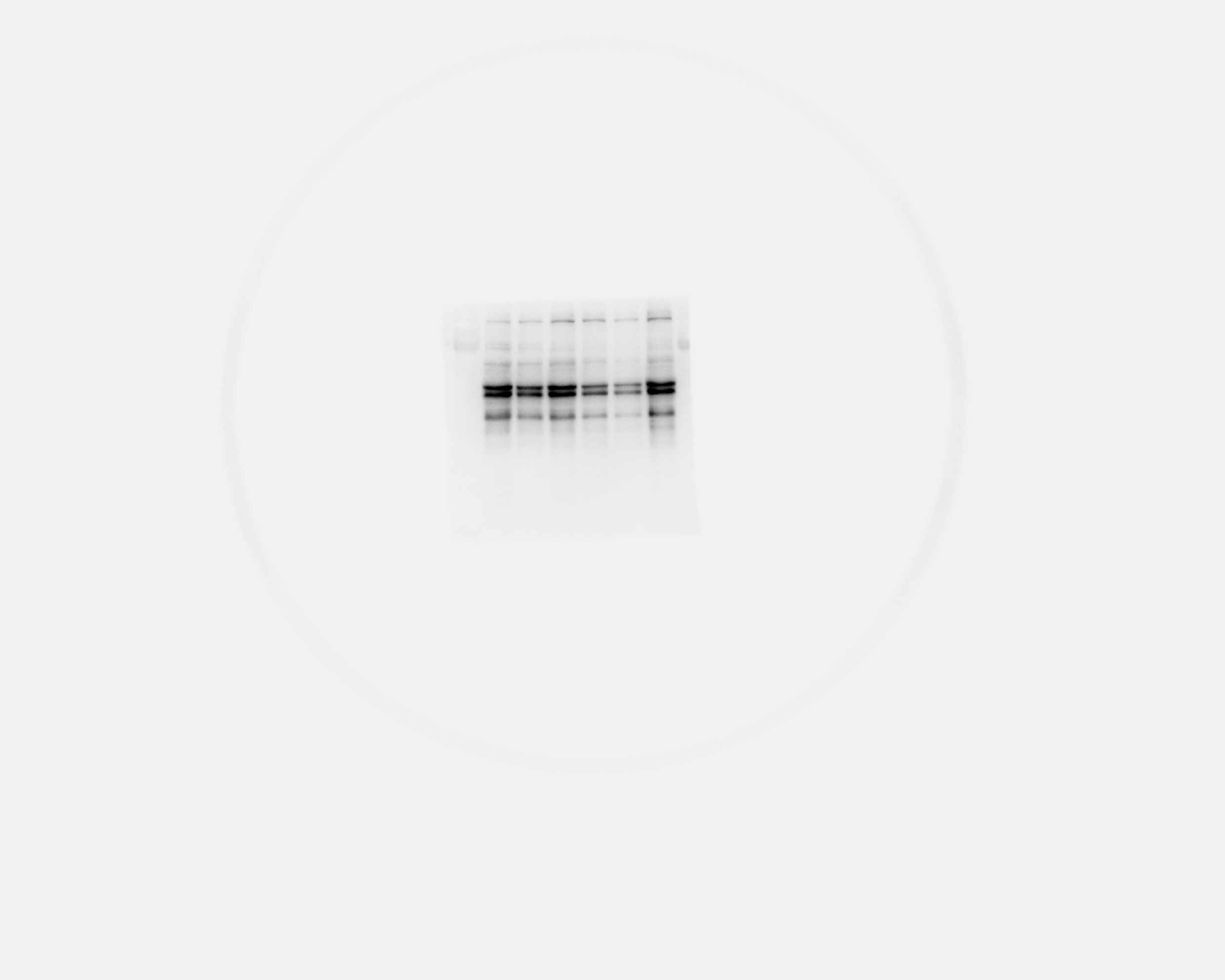

Supplement: Supplementary file 4 — Supplementary Material 4 [file 41598_2026_36354_MOESM4_ESM.zip › Full uncropped Gels and Blots image(s)/Fig.8/Fig.8C TXNIP-1.tif]

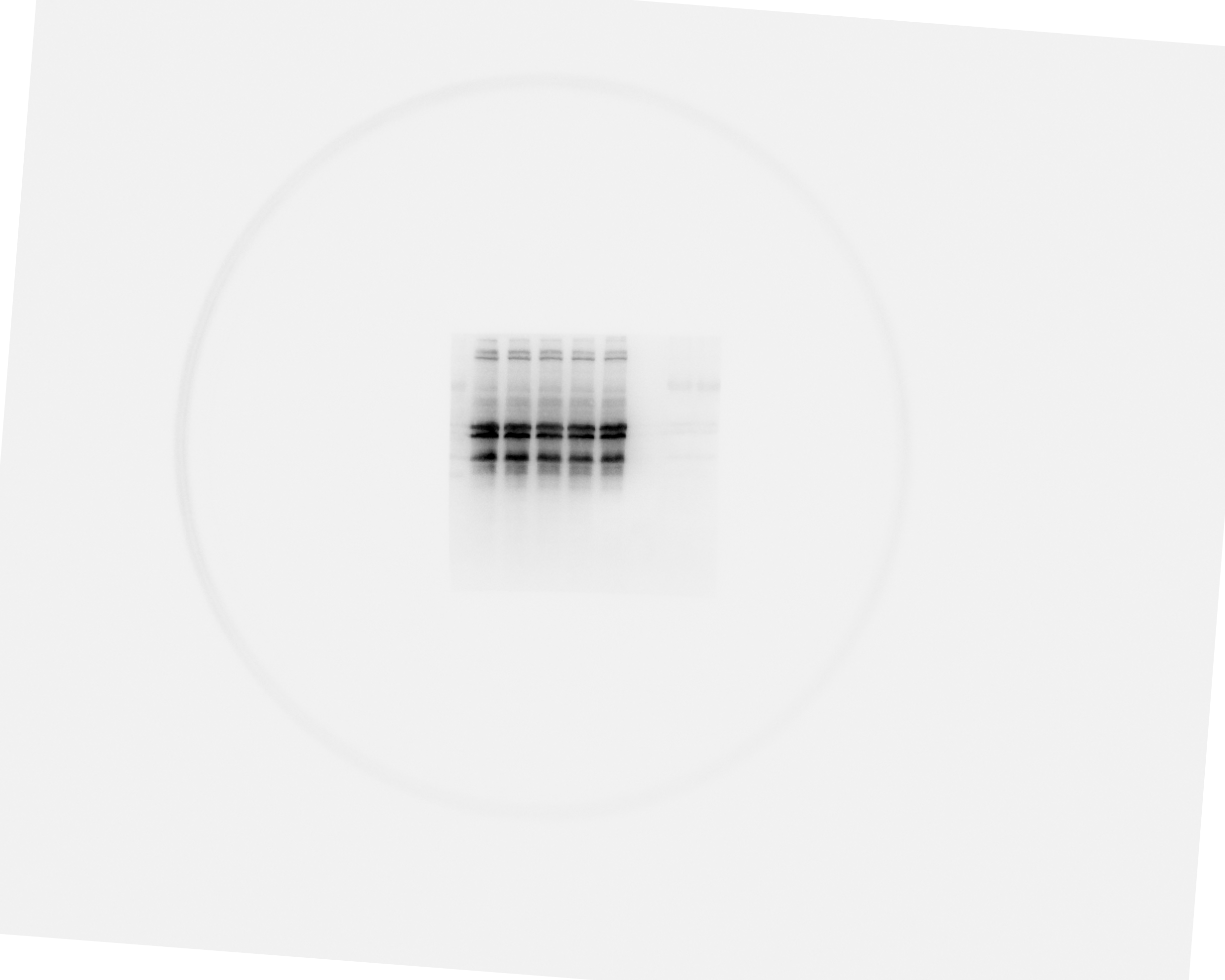

Supplement: Supplementary file 4 — Supplementary Material 4 [file 41598_2026_36354_MOESM4_ESM.zip › Full uncropped Gels and Blots image(s)/Fig.8/Fig.8C TXNIP-2.tif]

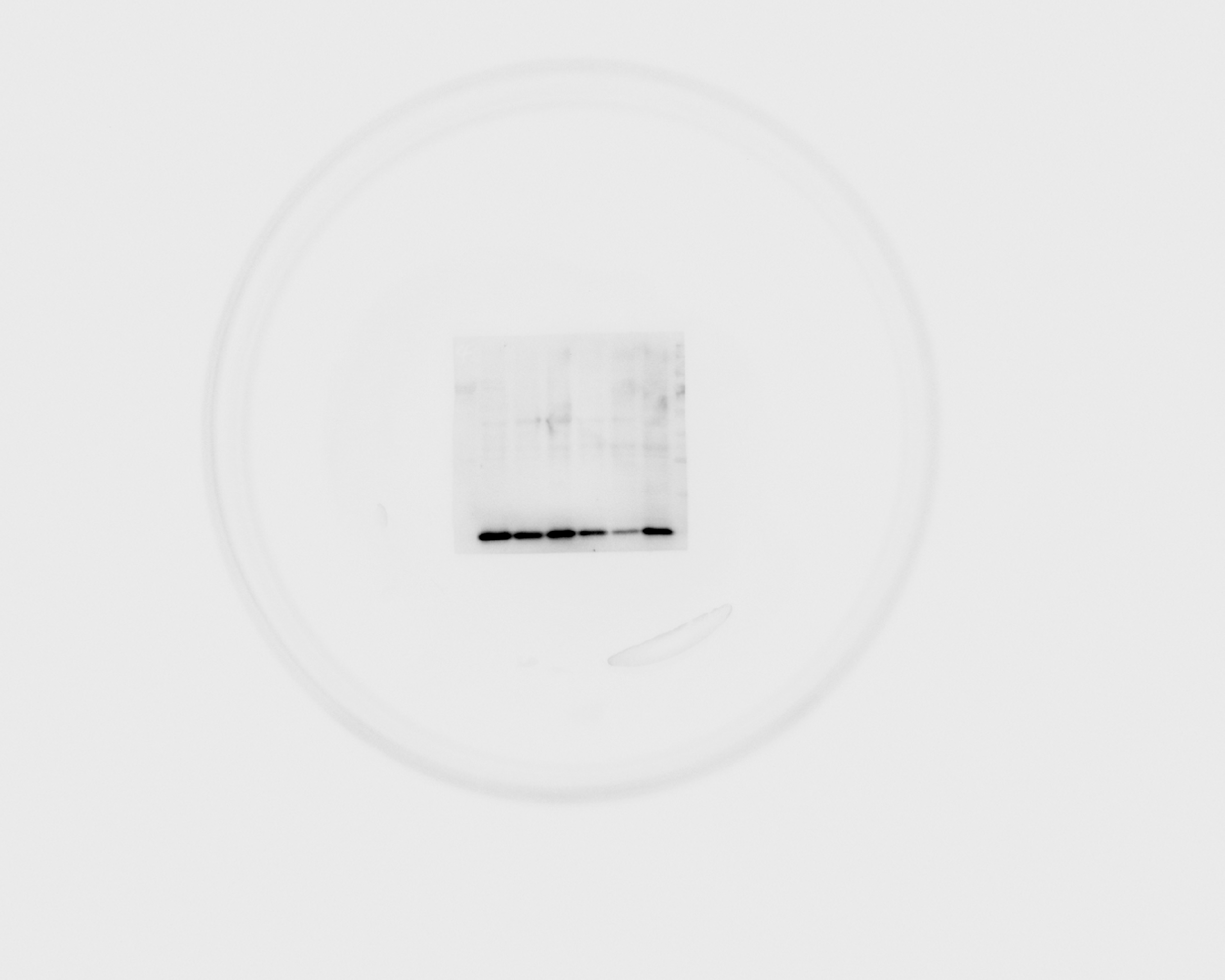

Supplement: Supplementary file 4 — Supplementary Material 4 [file 41598_2026_36354_MOESM4_ESM.zip › Full uncropped Gels and Blots image(s)/Fig.8/Fig.8C Trx1-1.tif]

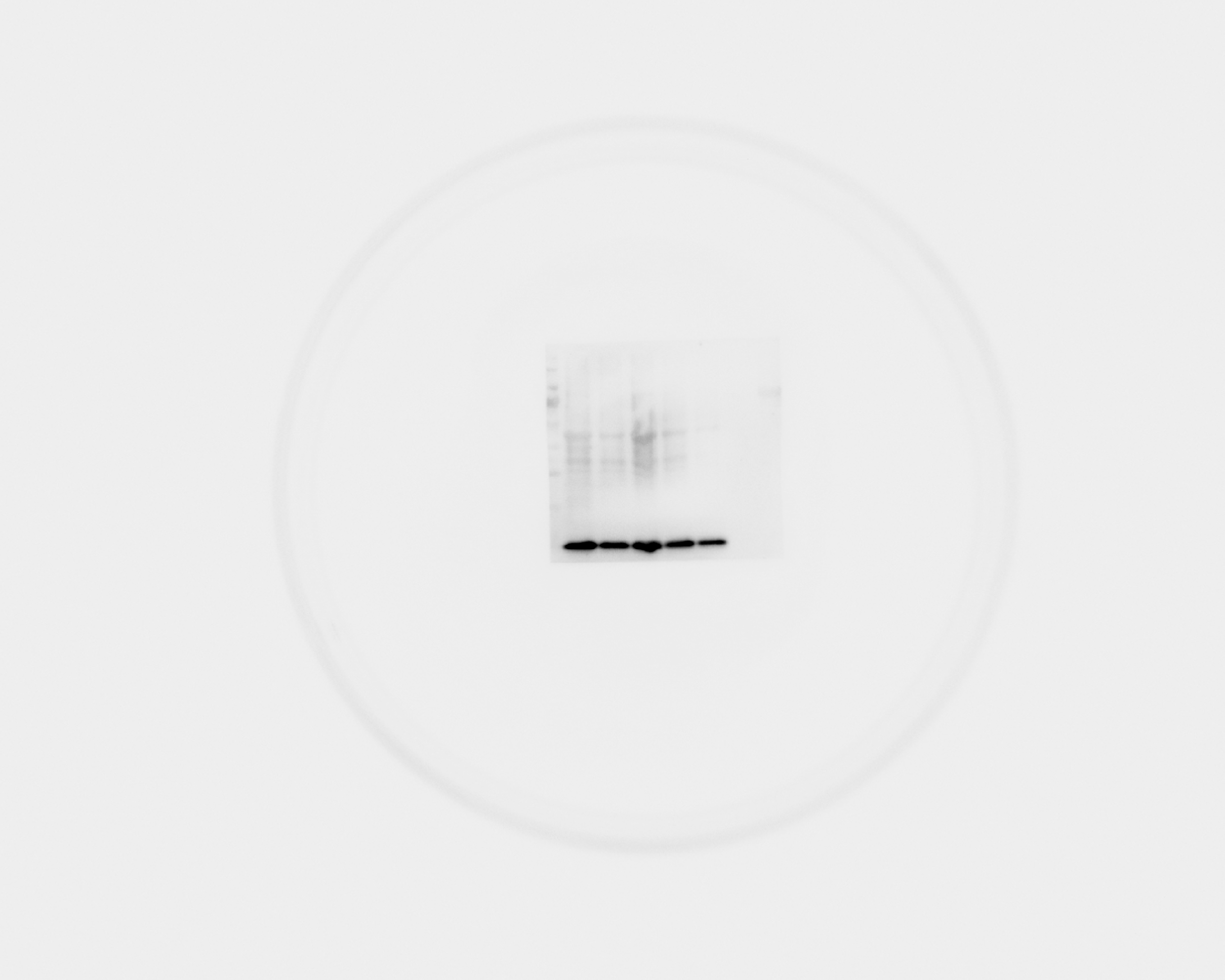

Supplement: Supplementary file 4 — Supplementary Material 4 [file 41598_2026_36354_MOESM4_ESM.zip › Full uncropped Gels and Blots image(s)/Fig.8/Fig.8C Trx1-2.tif]

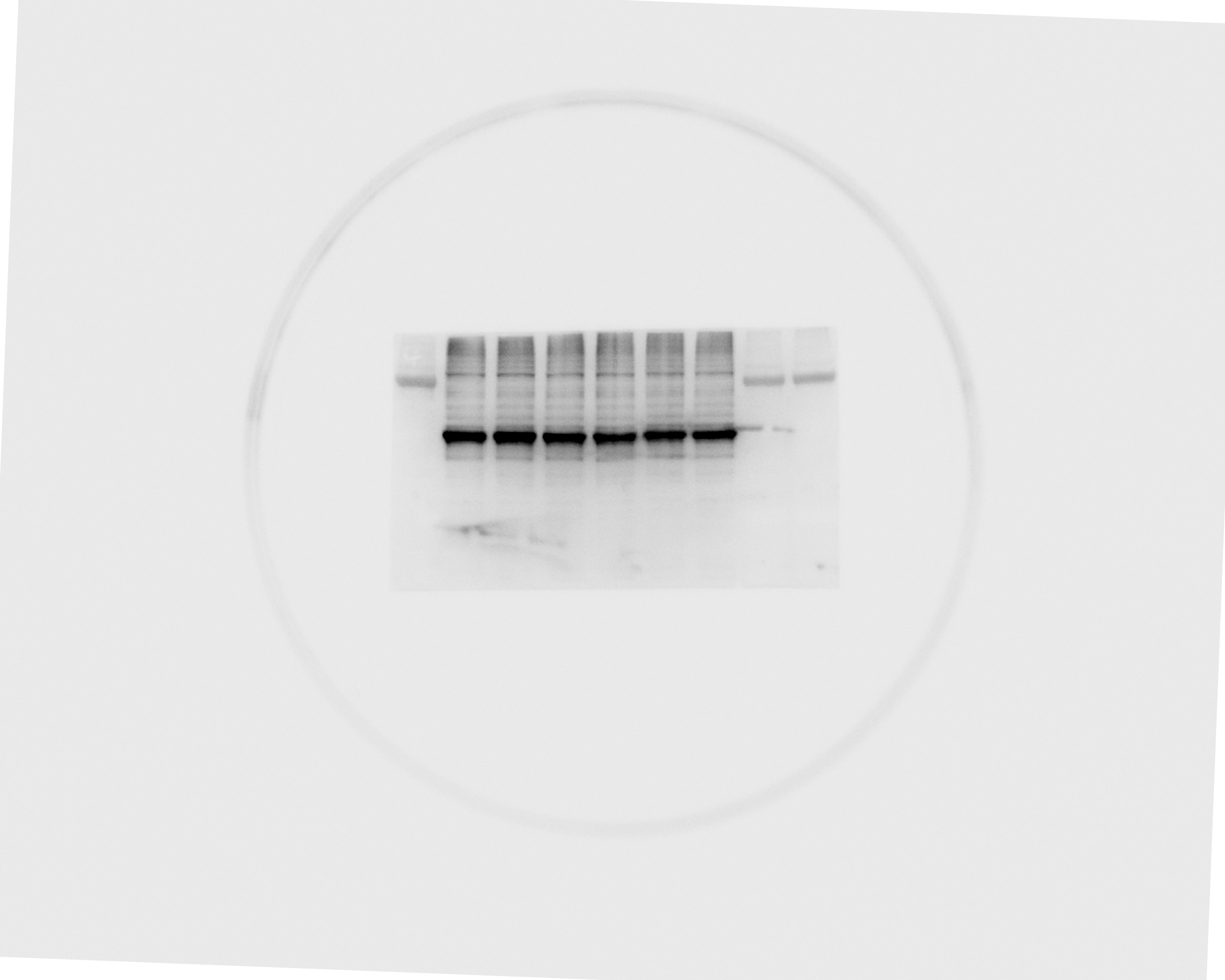

Supplement: Supplementary file 4 — Supplementary Material 4 [file 41598_2026_36354_MOESM4_ESM.zip › Full uncropped Gels and Blots image(s)/Fig.8/Fig.8C β-actin-2.tif]
